# Effectiveness of dietary, physical activity, and lifestyle interventions on weight and cardiometabolic outcomes in adults with overweight or obesity: an umbrella review of 112 systematic reviews and meta-analyses from 50 countries

**DOI:** 10.1016/j.obpill.2026.100324

**Published:** 2026-09-01

**Authors:** Mahesh Kumar Mummadi, Subbarao M. Gavaravarapu, Samarasimha N. Reddy, Karthikeyan Ramanujam, Ritika Prakash, Sudhakar Reddy Vadde, Raghavendra Pandurangi, Priyanka G. Bansal, Ranadip Chowdhury, Bharati Kulkarni

**Affiliations:** a Indian Council of Medical Research – National Institute of Nutrition (ICMR-NIN), Hyderabad, Telangana, 500007, India; b Academy of Scientific and Innovative Research (AcSIR), Ghaziabad, 201002, India; c Indian Council of Medical Research (ICMR), New Delhi, 110029, India; d Society for Applied Studies (SAS), New Delhi, 110016, India

**Keywords:** Obesity, Overweight, Umbrella review, Dietary intervention, Physical activity, Lifestyle intervention

## Abstract

**Background:**

Overweight and obesity affect an estimated 2.5 billion adults worldwide. Reviews of single treatment modalities are abundant, but clinicians lack one appraised comparison of nutrition therapy, physical activity, and behavioural modification across outcomes and treatment durations.

**Methods:**

This was an umbrella review and meta-analysis of systematic reviews and meta-analyses of randomized controlled trials in adults aged 18 years or older with overweight or obesity. PubMed was searched (2015–2025) with backward and forward citation tracking. Quality was appraised with AMSTAR 2 and certainty of evidence with GRADE. Primary outcomes were mean change in weight and body mass index (BMI) by intervention type and follow-up duration (<3, 3–6, 6–12, and >12 mo); secondary outcomes were glycaemic, lipid, and blood pressure parameters. Estimates were pooled using random-effects models.

**Results:**

Of 1725 records, 112 reviews (424 comparisons; 2332 unique randomized trials) were included. All intervention types produced significant but modest weight reduction. Combined diet and physical activity yielded the largest weight reduction beyond 12 mo (mean difference −3.68 kg; 95% CI: −5.47, −1.89; single contributing review), the largest BMI reduction at <3 mo (−1.22 kg/m^2^; 95% CI: −2.31, −0.13), and the greatest improvements in fasting glucose and insulin resistance. Pooled fasting glucose and lipid estimates combine effects reported on differing scales and are exploratory and descriptive only, not absolute mg/dL values. Physical activity produced the largest systolic blood pressure reduction (6–12 mo: −6.10 mm Hg; 95% CI: −8.07, −4.13). Lipid effects were inconsistent, heterogeneity was high (I^2^ commonly 70%–99%), and AMSTAR 2 confidence was low or critically low in 69% of reviews.

**Conclusion:**

Most non-pharmacological approaches reduced weight and BMI, and multicomponent programs combining diet, physical activity, and behavioural support gave the greatest cardiometabolic benefit. Because this benefit was partly independent of the weight lost, such programs remain worthwhile for patients who do not reach conventional 5%–10% weight-loss targets.

**Registration:**

PROSPERO CRD420251245029 (https://www.crd.york.ac.uk/prospero/display_record.php?RecordID=1245029).

## Highlights

• Most non-pharmacological interventions modestly reduced weight and BMI.
• Combined diet plus activity gave the largest long-term weight loss.
• Exercise gave the largest fall in systolic blood pressure at 6–12 months.
• Cardiometabolic gains were partly independent of weight loss magnitude.
• **For practice:** Even 1–2 kg lowers diabetes, hypertension and cardiovascular risk — worthwhile when 5–10% weight-loss targets are out of reach.

## Graphical abstract

**Figure:**
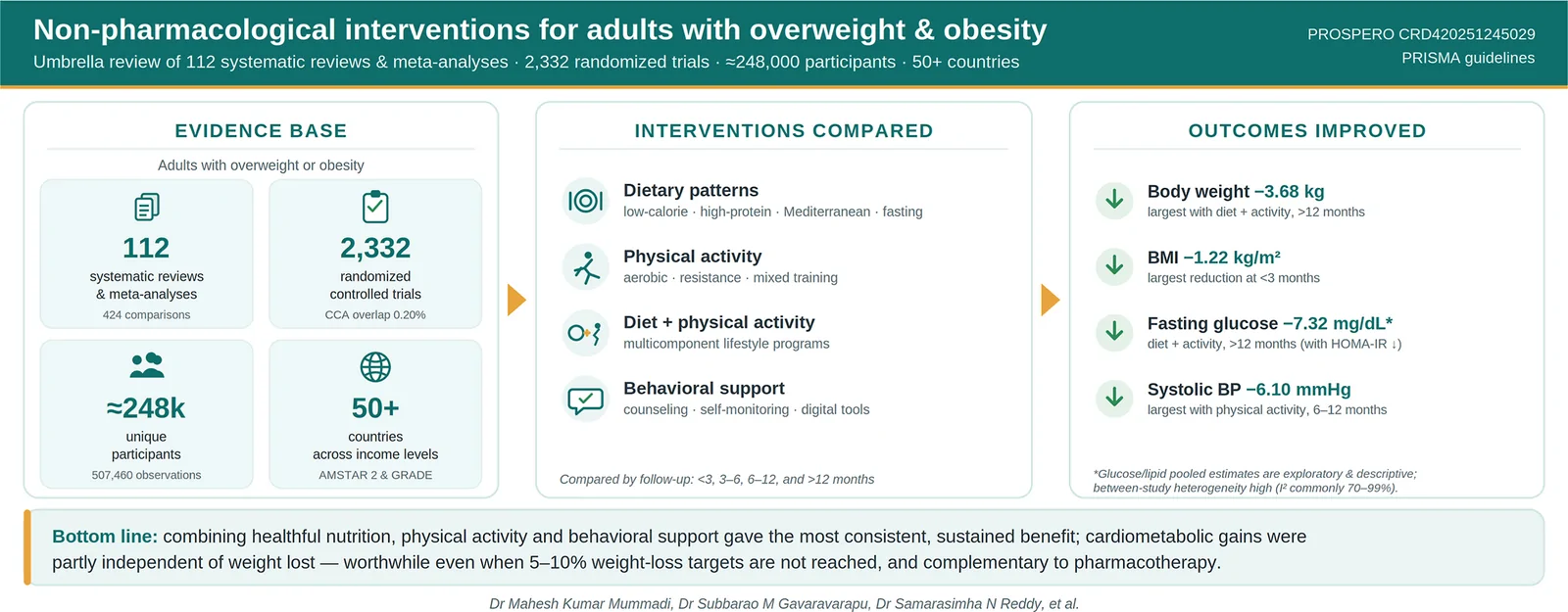

## Abbreviations

AMSTAR 2: A MeaSurement Tool to Assess systematic Reviews, version 2
BMI: body mass index
CCA: corrected covered area
FBG: fasting blood glucose
GRADE: Grading of Recommendations Assessment, Development and Evaluation
HbA1c: glycated hemoglobin
HDL: high-density lipoprotein
HOMA-IR: homeostatic model assessment of insulin resistance
LDL: low-density lipoprotein
LMIC: low- and middle-income country
MD: mean difference
PRISMA: Preferred Reporting Items for Systematic Reviews and Meta-Analyses
PROSPERO: International Prospective Register of Systematic Reviews
RCT: randomized controlled trial
REML: restricted maximum likelihood
SR: systematic review
VLCD: very low carbohydrate diet
VLCKD: very low-calorie ketogenic diet
VLED: very low energy diet
WHO: World Health Organization

## 1 Introduction

As countries undergo socioeconomic development and improvements in food security, shifts in dietary patterns and physical activity have increased. These transitions have fostered obesogenic environments, contributing to the growing global burden of overweight and obesity [1]. Obesity, as defined by the World Health Organization (WHO), is an abnormal or excessive accumulation of body fat that presents a risk to health [2]. Overweight and obesity are typically classified using body mass index (BMI), calculated as weight in kilograms divided by height in meters squared (kg/m^2^). A BMI ≥25 kg/m^2^ is considered overweight, and a BMI ≥30 kg/m^2^ is classified as obesity [1].

Obesity has emerged as one of the most pressing public health challenges, affecting populations across all regions and socioeconomic strata [3]. In the absence of effective preventive and policy interventions, projections indicate that the global prevalence of obesity will continue to rise markedly, potentially doubling by 2030 [1]. Once considered a health concern predominantly affecting affluent Western societies, obesity now imposes a disproportionately increasing burden on low- and middle-income countries, where urbanization, nutrition transitions, and sedentary lifestyles have accelerated its rise [4].

Obesity emerges from a complex interplay of biological, physiological, and environmental factors that collectively disrupt energy homeostasis, and its consequences extend far beyond excess weight. With associations spanning more than 50 comorbid conditions from type 2 diabetes, hypertension, and cardiovascular disease to depression, dementia, osteoarthritis, chronic kidney disease, and obstructive sleep apnea [3]. Despite this substantial global burden, evidence to guide scalable non-pharmacological prevention and management strategies remains fragmented. Existing systematic reviews and meta-analyses are limited by heterogeneity in intervention design, outcome selection, and methodological quality, and most address a single modality or report anthropometric outcomes in isolation rather than alongside the glycemic, lipid, and blood-pressure changes that are central to obesity-related morbidity.

Few syntheses critically appraise the credibility and methodological robustness of these reviews with standardized tools, leaving clinicians and policymakers without a consolidated, high-level comparison of the relative effectiveness of different intervention types across populations and settings. We therefore did an umbrella review to synthesize, compare, and critically appraise systematic reviews and meta-analyses of dietary, physical activity, lifestyle, and combined non-pharmacological interventions in adults with overweight or obesity, evaluating their effects on body weight, BMI, and cardiometabolic outcomes across intervention types and follow-up durations.

## 2 Methods

### 2.1 Search strategy and selection criteria

This was an umbrella review and meta-analysis conducted in accordance with PRISMA 2020 (Preferred Reporting Items for Systematic Reviews and Meta-Analyses) [5], and adherence to the PRISMA checklist is reported in Supplemental Table 6. To identify relevant systematic reviews and meta-analyses, we performed a comprehensive search of the PubMed database using a PICO-structured strategy that combined MeSH terms and free-text keywords related to obesity, overweight, dietary patterns, physical activity, lifestyle interventions, and weight management, linked with Boolean operators (Supplemental Table 1). We searched PubMed as the primary index database and supplemented it with systematic backward (reference-list) and forward (citation-tracking) searching of all included reviews, an approach shown to recover records missed by single-database searches; these supplementary routes yielded a further 175 records (10% of the pooled total), indicating they materially broadened coverage. Importantly, the reviews we included had themselves collectively drawn upon at least six biomedical databases (Cochrane, Scopus, Embase, Web of Science, Ovid, and PubMed), which partially offsets any source bias from our single-database search. We restricted our search to English-language articles published between 2015 and 2025 to ensure a contemporary evidence base. Beyond the primary search, we conducted supplementary identification through manual reference checking of all included reviews and forward citation tracking via Google Scholar, applying identical eligibility criteria to records identified through these routes. The protocol was prospectively registered with PROSPERO (CRD420251245029).

We included systematic reviews and meta-analyses of RCTs that enrolled adults aged ≥18 years and evaluated dietary, physical activity, lifestyle, or combined non-pharmacological interventions against usual care or another active non-pharmacological comparator. Primary outcomes of interest were body weight and BMI, secondary outcomes included glycemic markers, lipid profile, and blood pressure. Reviews were excluded if they focused exclusively on children or adolescents, pharmacological or surgical interventions, or disease populations not primarily defined by overweight or obesity. Narrative reviews, scoping reviews, protocols, conference abstracts, editorials, and commentaries were not eligible.

All records were imported into Rayyan [6] for bibliographic management and with duplicate records identified during full text screening manual verification. Two reviewers (SNR and RPK) independently screened titles and abstracts, followed by full-text review of all 1552 retrieved reports. Disagreements at any stage were resolved through discussion and consensus, with a third reviewer (MKM) consulted when needed.

Data were extracted using a standardized, pilot-tested form capturing study metadata (lead author, year, number of included primary studies), participant characteristics (total sample size, age distribution), intervention details (components, duration, frequency, and mode of delivery), quantitative effect estimates (mean difference and 95% CI), and the countries and databases represented in each review.

### 2.2 Quality appraisal and certainty of evidence

Methodological quality was independently assessed using the AMSTAR 2 [7] instrument across its 16 domains, classifying each review as having high, moderate, low, or critically low confidence in its results (Supplemental Table 2). Certainty of evidence for each outcome was evaluated using the GRADE approach [8], beginning at high certainty given the RCT basis of all included reviews and downgrading for risk of bias, inconsistency, indirectness, imprecision, or publication bias as appropriate (Supplemental Table 3).

To enable a coherent comparative analysis, interventions were organized into five thematic clusters: dietary interventions, physical activity, lifestyle-based approaches, combined diet and physical activity, and multi-component interventions encompassing all three elements. To examine whether effects persisted over time, we stratified subgroup analyses by follow-up duration: short-term (<3 months), medium-term (3–6 months), long-term (6–12 months), and extended-term (>12 months).

### 2.3 Intervention classification

**Dietary interventions** were further sub-classified by type. **Low-calorie diets** included VLCKD (very low-calorie ketogenic diet: <50 g carbohydrate/day, approximately 500–800 kcal/day), VLED (very low energy diet), low-carbohydrate diet (carbohydrates ≤40% or 10–25% of total energy) [9], low-fat diet, energy-restricted diet, and VLCD (very low carbohydrate diet: carbohydrates <10% of total energy). **Intermittent fasting**, defined as consuming ≤800 kcal on one to six days per week, encompassed zero-calorie alternate-day fasting, modified alternate-day fasting, the 5:2 regimen, Ramadan fasting, and time-restricted eating (daily eating window <12 h; common patterns included 16:8, 14:10, and 12:12). **High-protein diets** were characterized by protein intake ≥20–30% of total energy, with or without overall caloric restriction. Other dietary interventions covered a range of approaches including whole grains, curcumin supplementation, mixed nuts [10,11], increased water intake, plant-based diets (vegan, vegetarian, and their variants), egg consumption, the paleolithic diet, and pomegranate consumption.

**Physical activity interventions** comprised structured aerobic exercise, resistance training, or combined programs delivered in supervised or unsupervised settings. Typical durations ranged from 30 to 60 min per session for moderate-intensity programs to 45–60 min for higher-intensity or resistance-focused regimens, generally prescribed at 2–3 sessions per week.

**Lifestyle interventions** centered on behavioral and habit-based strategies, including behavioral counselling, self-monitoring, goal setting, health education, and motivational support, with some studies also incorporating stress management and sleep improvement components.

**Combined diet and physical activity** interventions paired calorie restriction or structured meal planning with aerobic and resistance exercise, while **multi-component interventions** additionally integrated behavioral counselling, self-monitoring, and health education into a single program.

### 2.4 Data synthesis and statistical analysis

We quantified the overlap of primary trials across reviews using the corrected covered area (CCA), calculated as CCA = (A − N)/[N × (r − 1)], where A is the total number of trial inclusions, N the number of unique trials, and r the number of reviews [12]. Values were interpreted as slight (≤5%), moderate (6–10%), high (11–15%), or very high (>15%) overlap, and pairwise overlap between review pairs was also computed. The stability of the CCA was checked in a high-reliability subset of reviews (Cochrane reviews and those with complete trial-level extraction), which yielded a comparable estimate (CCA 0.60%) and a stable A/N ratio (1.23), supporting the slight-overlap classification.

For each outcome we pooled the reported effect estimates within intervention category and follow-up stratum using a random-effects model with restricted maximum likelihood (REML) estimation of between-study variance, presenting results as forest plots (Fig. 2, Fig. 3, Fig. 4, Fig. 5, Fig. 6), with full stratum-level estimates provided in Supplemental Fig. 1, and the certainty of evidence for each outcome summarized in Supplemental Table 3. Anthropometric outcomes (weight and BMI) were analyzed as mean differences; for the cardiometabolic outcomes, estimates were analyzed in the metric reported by each contributing review. For fasting blood glucose and the four lipid outcomes, contributing reviews reported effects on differing scales (unstandardized mean differences in mg/dL alongside standardized mean differences); the pooled estimates for these outcomes therefore combine values that are not on a common scale and are presented as exploratory and descriptive only, and should not be interpreted as absolute mg/dL changes. Heterogeneity was quantified with I^2^ (low <25%, moderate 25–75%, high >75%). Subgroup analyses and meta-regression were undertaken only where at least ten estimates were available. Small-study effects and potential reporting bias were assessed, where at least ten estimates contributed to an outcome, using Egger's regression test and the trim-and-fill method, which imputes potentially missing studies to restore funnel-plot symmetry and recalculates the pooled estimate. We did three prespecified sensitivity analyses: exclusion of small reviews (≤100 participants), a leave-one-out approach, and re-estimation under a fixed-effects model. Analyses were done in Stata 18.0 (StataCorp, College Station, TX, USA).

**Fig. 1:**
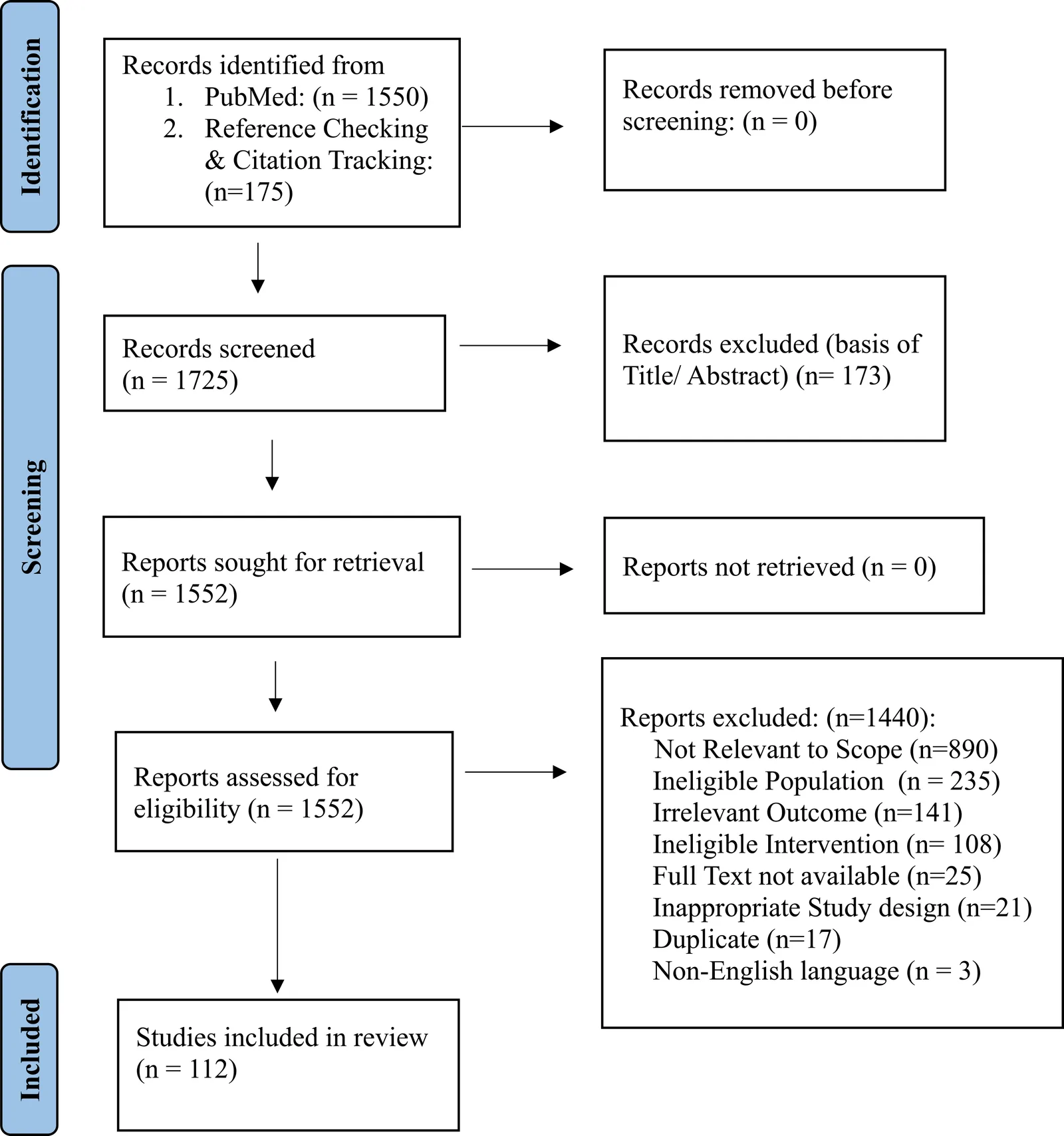
Preferred Reporting Items for Systematic Reviews and Meta-Analyses (PRISMA) 2020 flow diagram of study identification, screening, and inclusion. Records were identified from PubMed and supplemented by backward reference checking and forward citation tracking.

**Fig. 2:**
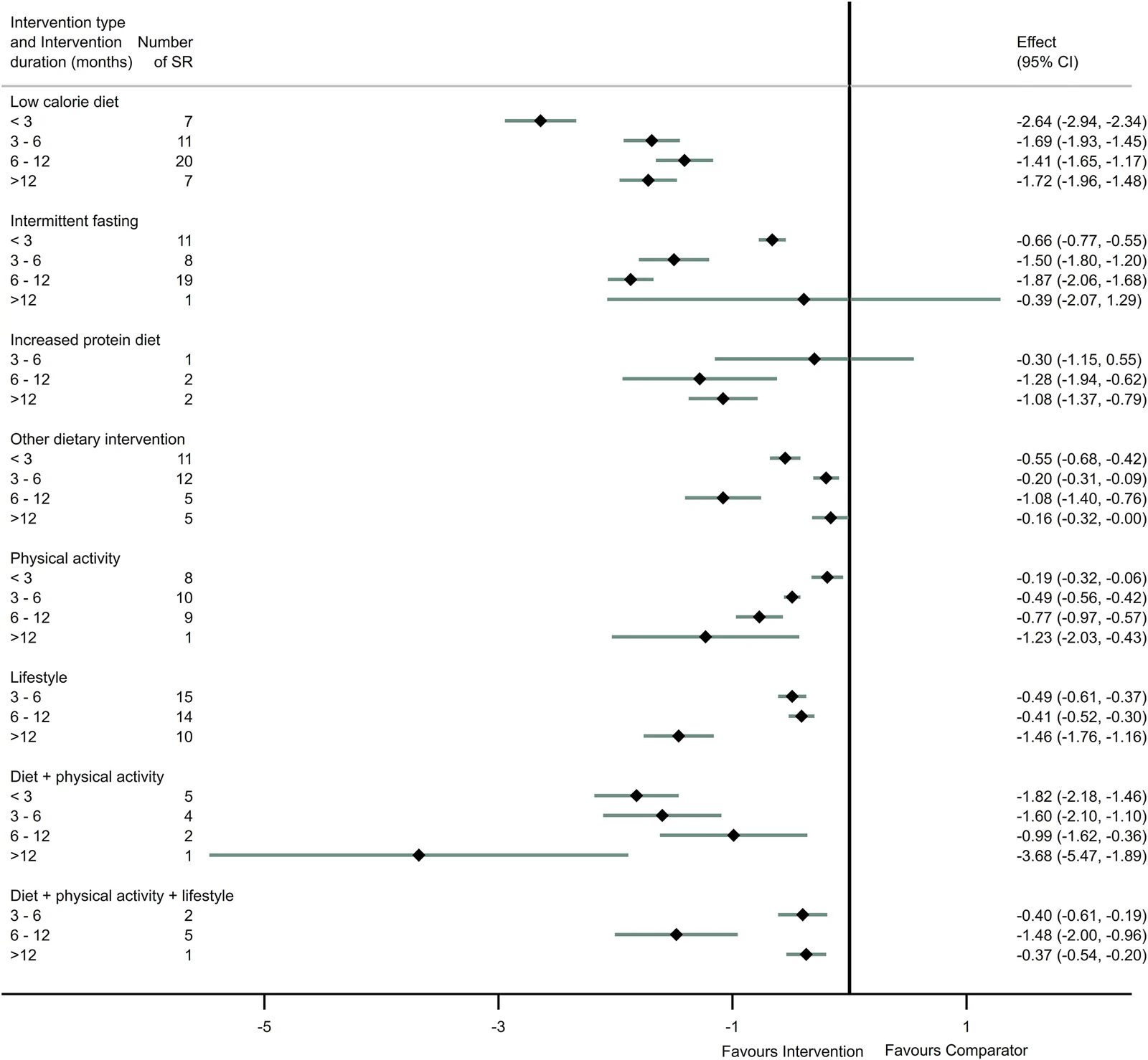
Forest plots showing pooled mean weight change (kg) by intervention type and duration.

**Fig. 3:**
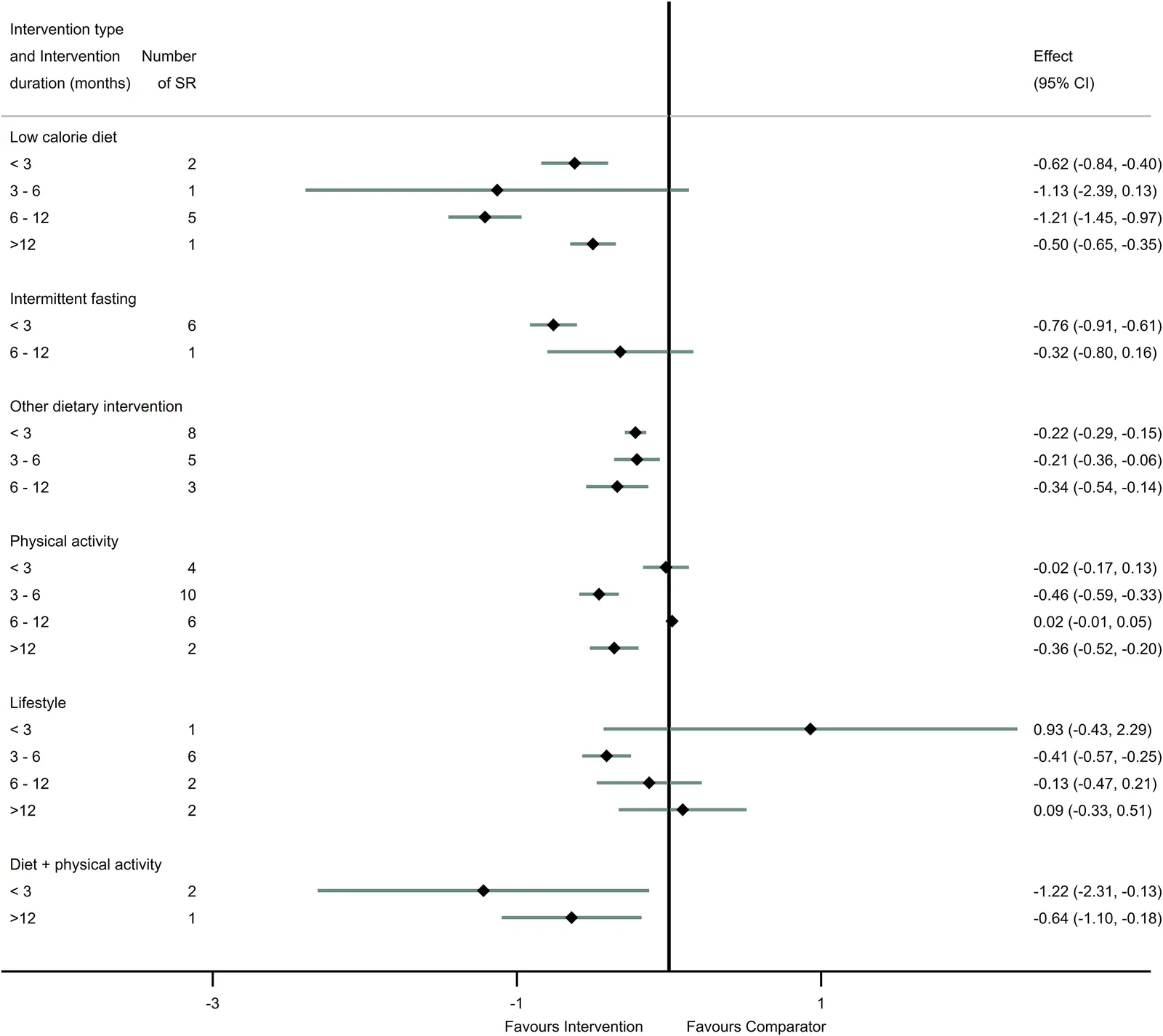
Forest plots showing pooled mean body mass index (BMI) change (kg/m^2^) by intervention type and duration.

**Fig. 4:**
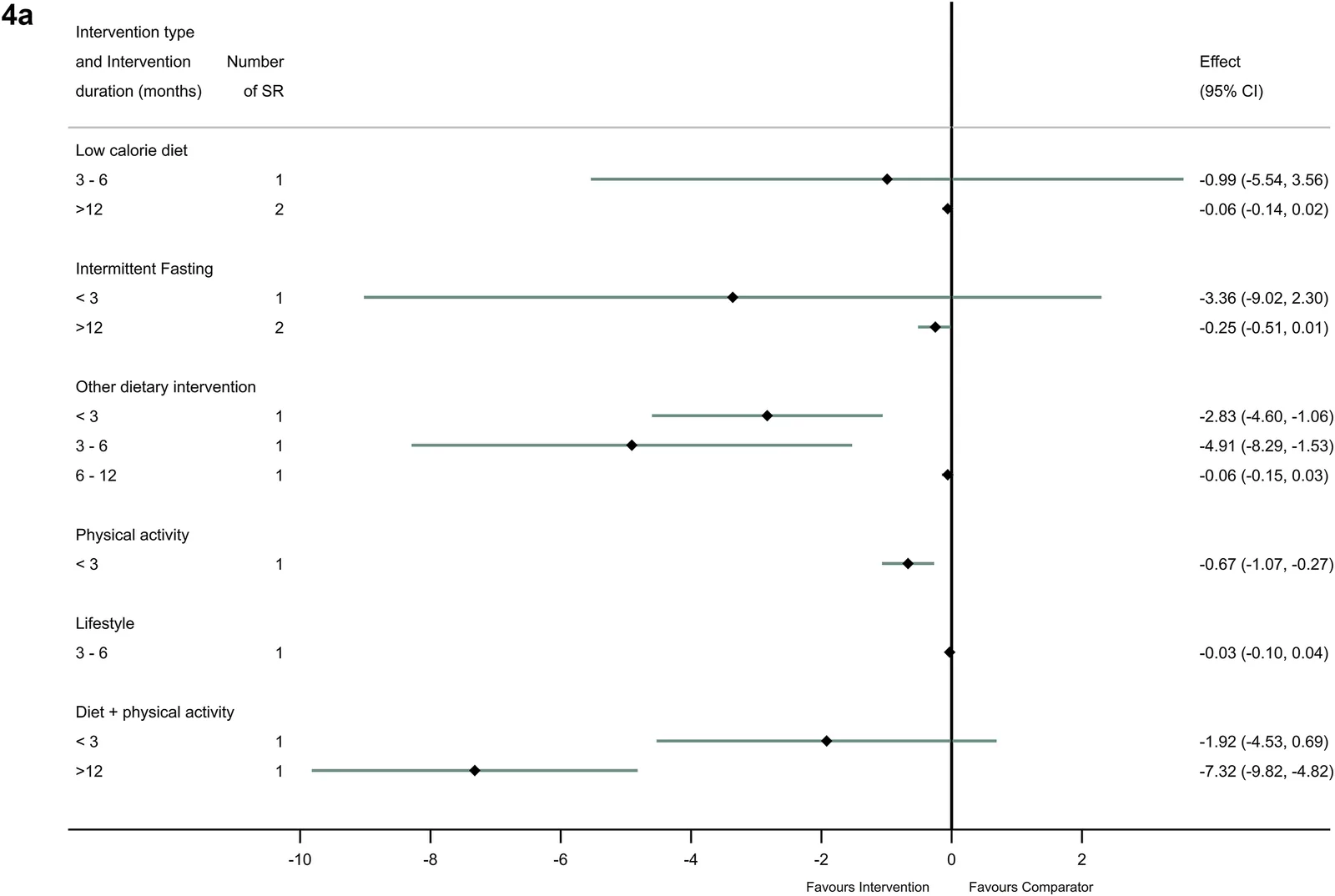

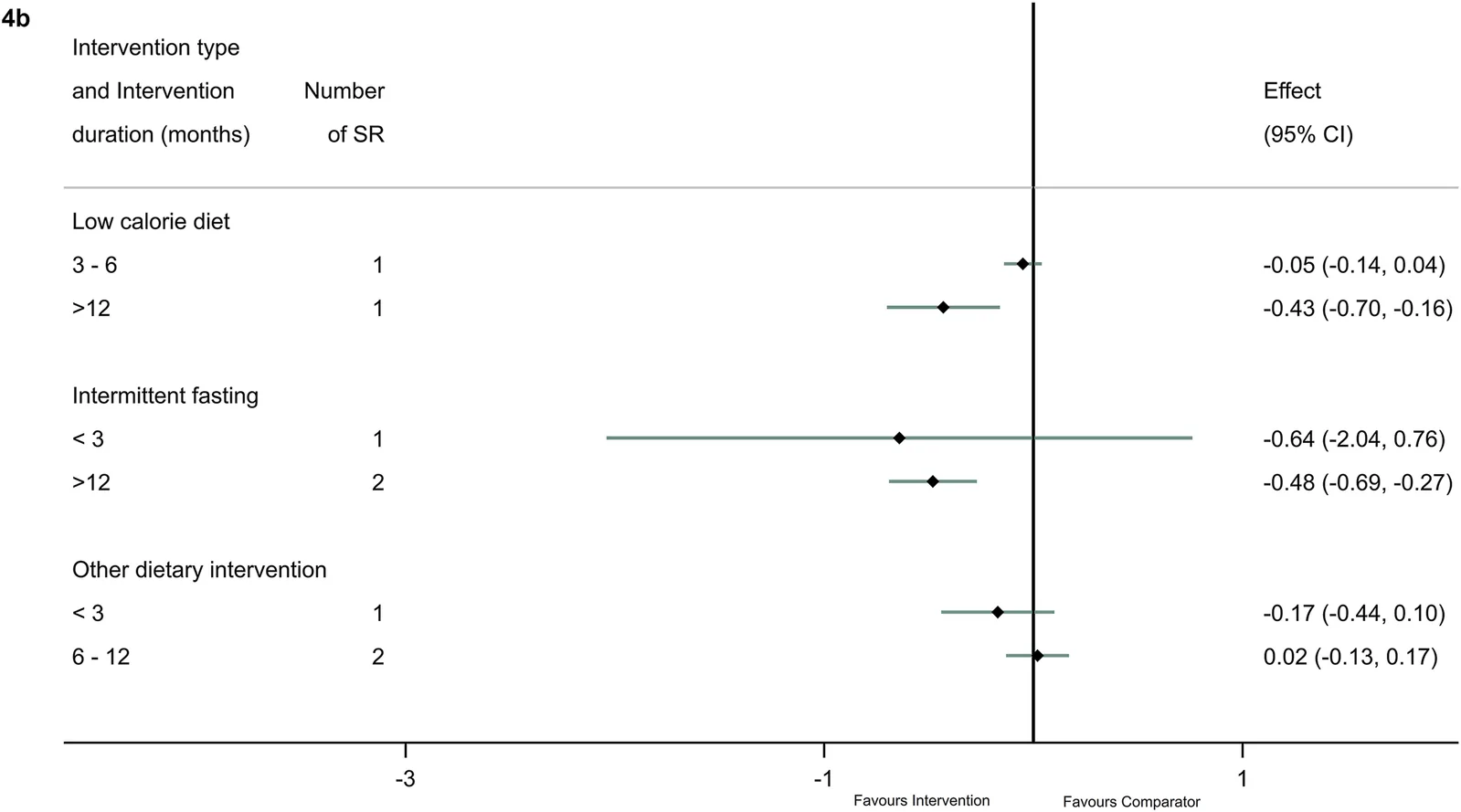

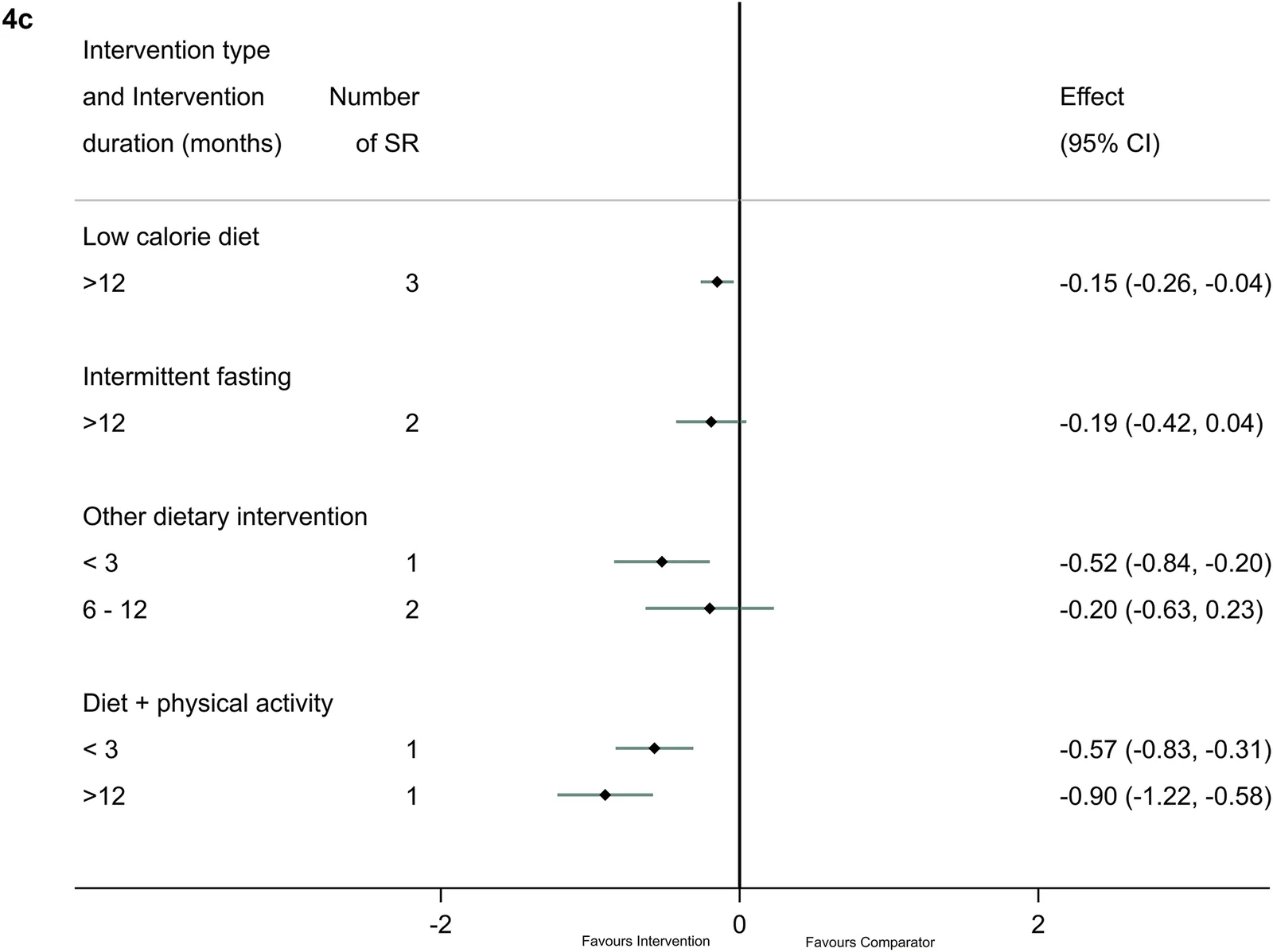
Forest plots showing pooled mean change in glycemic parameters-fasting blood glucose (mg/dL, milligrams per deciliter) (4a), glycated hemoglobin (HbA1c) (%) (4b), and homeostatic model assessment of insulin resistance (HOMA-IR) (4c) by intervention type and duration

**Fig. 5:**
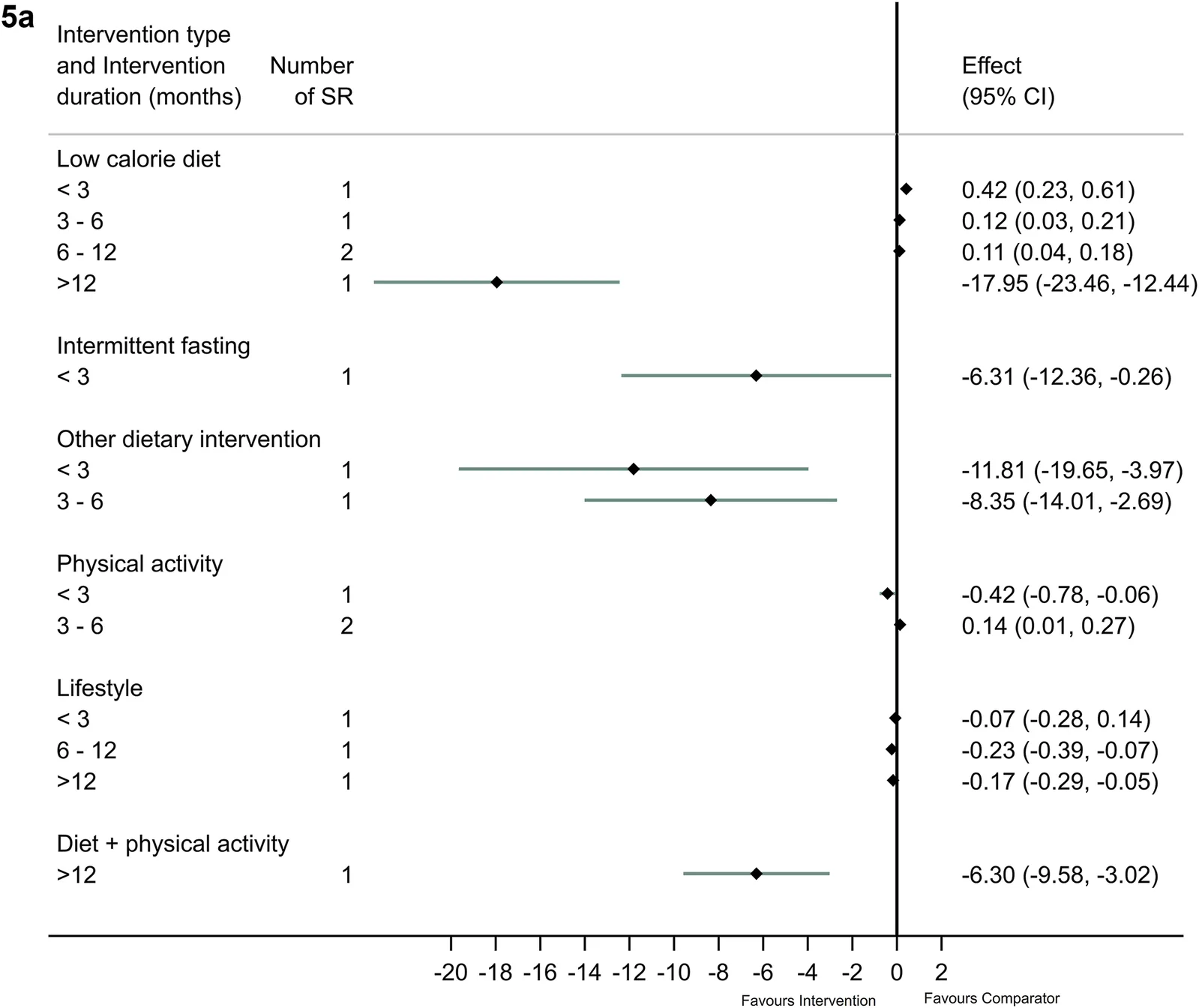

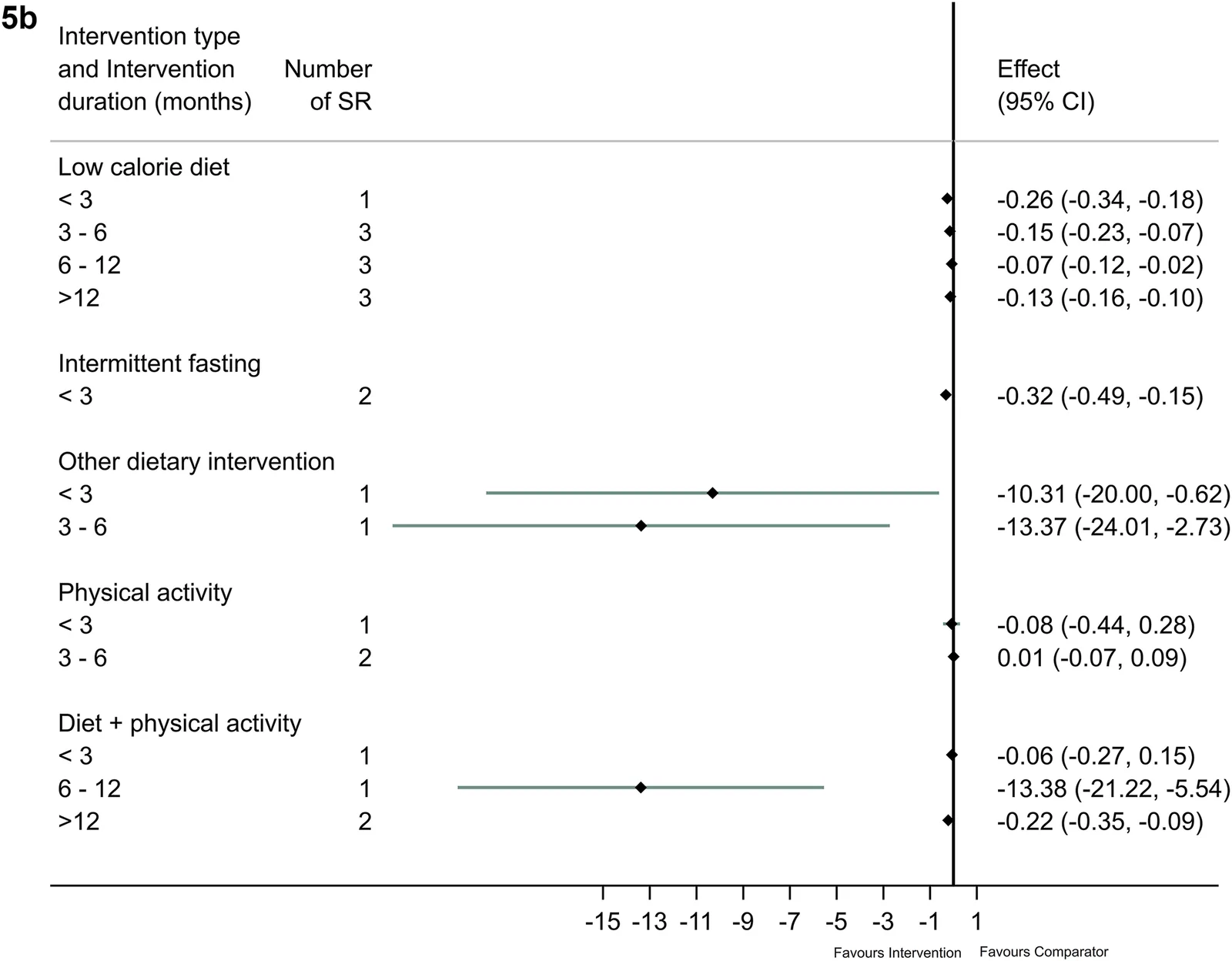

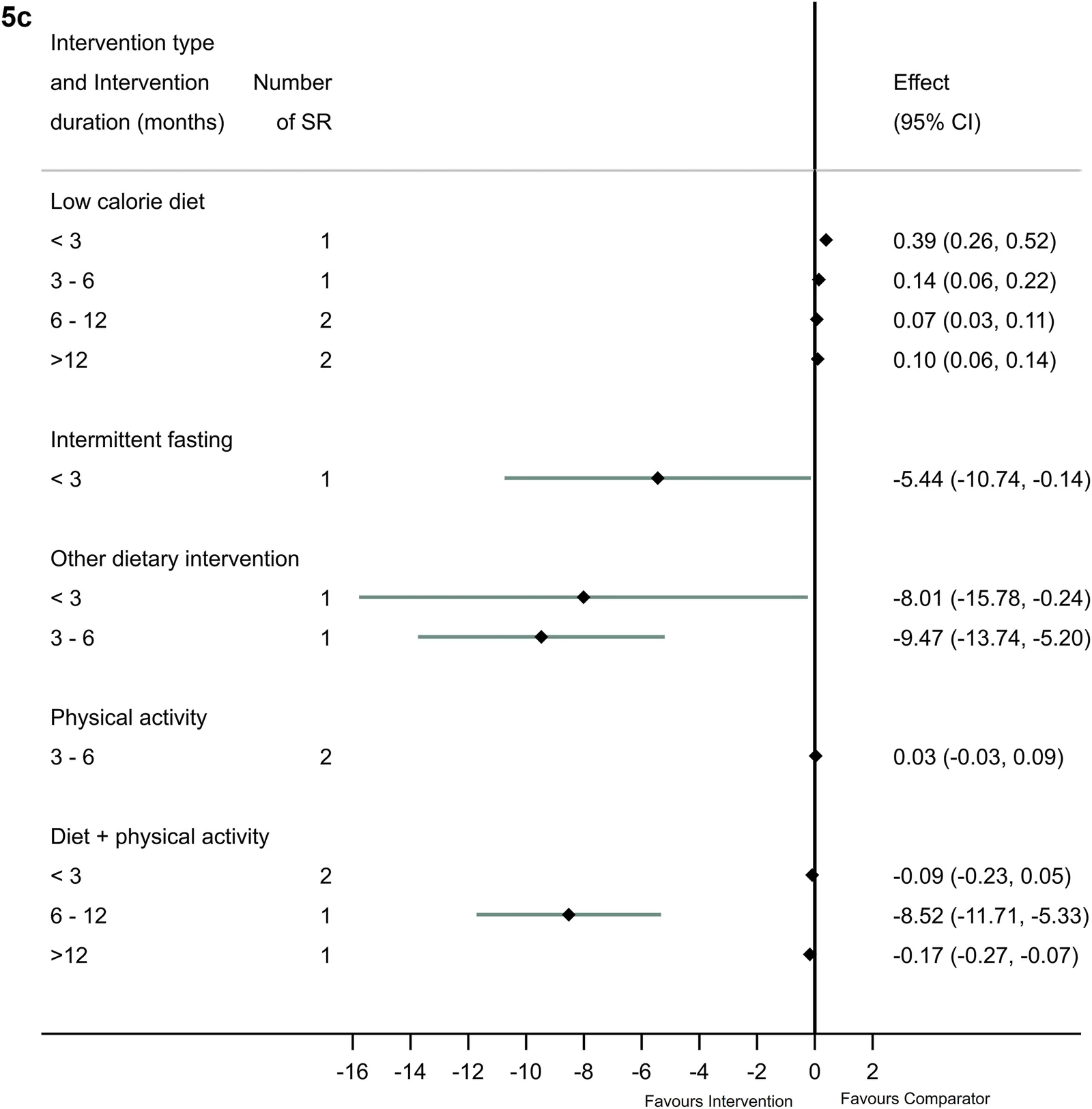

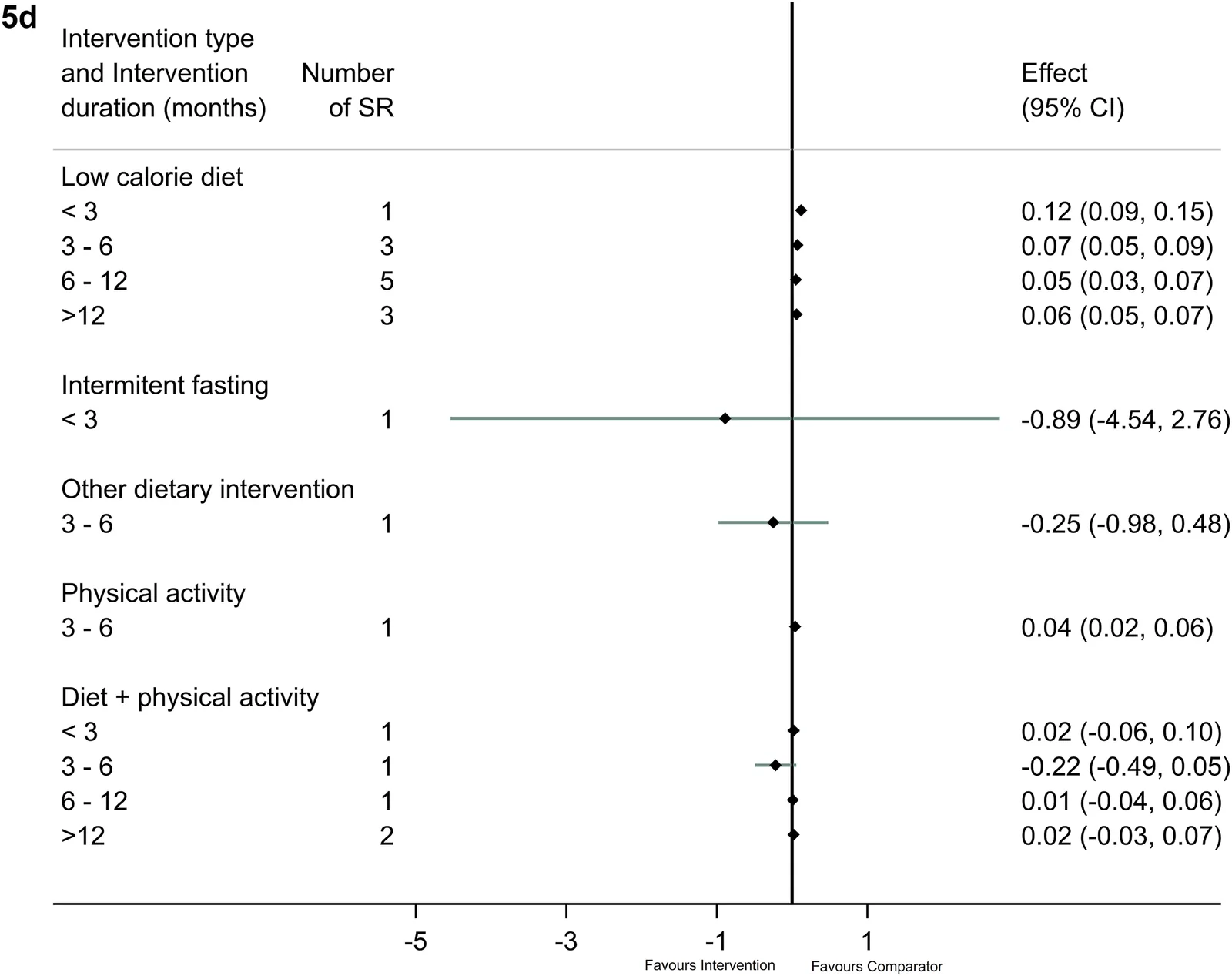
Forest plots showing pooled mean change in lipid parameters-total cholesterol (mg/dL, milligrams per deciliter) (5a), triglycerides (mg/dL, milligrams per deciliter) (5b), low-density lipoprotein (LDL) (5c) and high-density lipoprotein (HDL) cholesterol (mg/dL) (5d) by intervention type and duration.

**Fig. 6:**
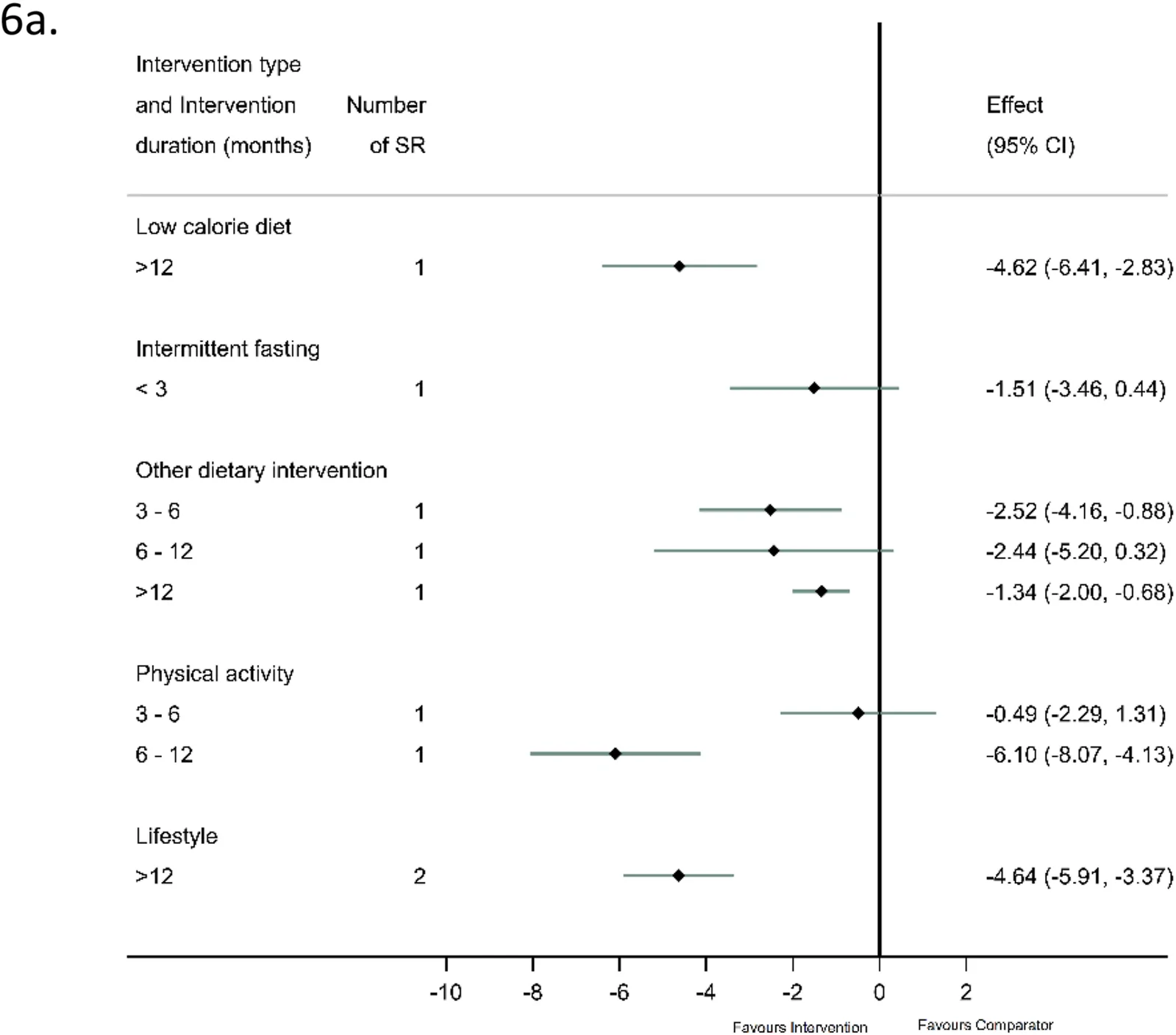

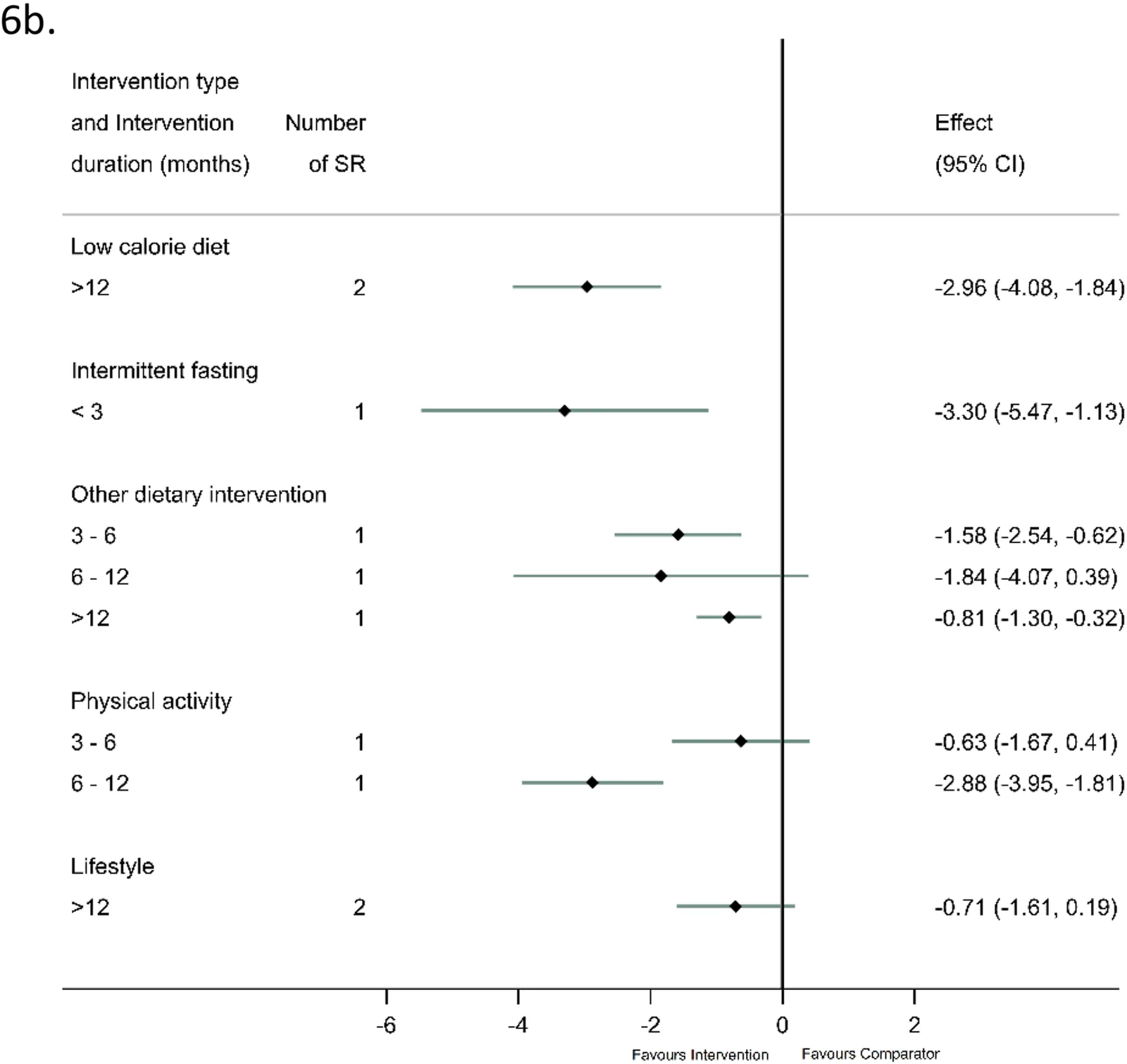
Forest plots showing pooled mean change in blood pressure-systolic (mmHg, millimeters of mercury) (6a) and diastolic (mmHg, millimeters of mercury) (6b) by intervention type and duration.

### 2.5 Ethics and patient and public involvement

This study used only published, publicly available data and required no ethics approval or patient consent. Patients and the public were not involved in the design or conduct of the review.

**Patient and public involvement.** Patients and the public were not directly involved in this umbrella review, as the synthesis drew exclusively on published literature without collecting any primary data.

## 3 Results

The database search identified 1550 records from PubMed, with a further 175 identified through reference checking and forward citation tracking, giving 1725 records after deduplication. Title and abstract screening excluded 173 records, and all 1552 reports sought were retrieved and assessed at full text. Of these, 1440 were excluded (not relevant to scope n = 890; ineligible population n = 235; irrelevant outcome n = 141; ineligible intervention n = 108; full text unavailable n = 25; inappropriate study design n = 21; duplicate n = 17; non-English n = 3); full list of exclusions with reasons in Supplemental Table 5, leaving 112 systematic reviews and meta-analyses for inclusion (Fig. 1; characteristics in Supplemental Table 4).

The 112 reviews contributed 424 intervention comparisons and 507,460 participant-observations, drawn from 2332 unique randomized controlled trials conducted across more than 50 countries, with most evidence originating from high-income settings; the estimated number of unique participants was 248,187. Although 79 of 112 reviews (71%) included trial data from at least one low- or middle-income country, this was driven almost entirely by upper-middle-income settings (China contributing to 46 reviews [41%] and Brazil to 29 [26%]); only 2 reviews (2%) drew on any low-income-country trial, and lower-middle-income countries such as India and Pakistan appeared in just 18 (16%) and 8 (7%) reviews respectively. Overlap between reviews was low (corrected covered area 0.20%; “slight” by Pieper's classification) with 1983 of 2332 trials (85.0%) appearing in only one review and no single trial appearing in more than nine; this low overlap reflects the breadth of scope across six largely distinct intervention paradigms—dietary composition, time-restricted eating, intermittent fasting, physical activity, behavioral programs, and very-low-calorie or meal-replacement diets—each drawing on a substantially separate trial pool, with landmark diet trials (e.g. Foster 2010, Gardner 2007, Sacks 2009) recurring across five to nine reviews where shared literature did exist. Comparisons were distributed across four follow-up strata: 105 at less than 3 months, 130 at 3–6 months, 119 at 6–12 months, and 70 beyond 12 months. Methodological quality was variable; using AMSTAR 2, 24 reviews (21%) were rated high, 11 (10%) moderate, 48 (43%) low, and 29 (26%) critically low confidence (Supplemental Table 2).

The intervention was mainly conducted in USA, Australia, Mediterranean region, India, Norway, Netherlands, Scotland, Thailand, UK, Denmark, Pakistan, Finland, Mexico, China, South Korea, Singapore, Czech Republic, Turkey, Italy, Spain, Sweden, Germany, Japan, Canada, Israel, New Zealand, Barbados, Qatar, South Africa, Brazil, Poland, Portugal, Belgium, Switzerland, Russia, Taiwan, Kenya, Greece, Croatia, Oceania, Iran, Serbia, Malaysia, France, Tunisia, Indonesia, Egypt, Ireland, Bahrain, Jordan, Hong Kong.

Our interventions were categorized based on duration into four groups: <3 months, 3–6 months, 6–12 months, and >12 months. A total of 424 interventions were considered for our final meta-analyses. 105 interventions were classified within the ≤3 months category, 130 interventions in the 3–6 months category, 119 interventions in the 6–12 months category, and 70 interventions in the >12 months category.

All intervention categories reduced body weight, with effects that were generally modest and varied by type and follow-up (Fig. 2; pooled estimates in Table 1). Low-calorie diets produced the largest short-term reduction (−2.64 kg, 95% CI −2.94 to −2.34 at <3 months), attenuating to between −1.41 and −1.72 kg over longer follow-up. Intermittent fasting reduced weight by −0.66 to −1.87 kg up to 12 months but showed no significant effect thereafter. Physical activity produced small reductions that increased with duration (−0.19 kg at <3 months to −1.23 kg beyond 12 months), as did lifestyle interventions (up to −1.46 kg beyond 12 months). High-protein diets showed sustained reductions at 6–12 months (−1.28 kg) and beyond 12 months (−1.08 kg). The largest reduction in any single stratum was for combined diet and physical activity beyond 12 months (−3.68 kg, 95% CI −5.47 to −1.89), although this estimate derived from one review.

**Table 1:** Pooled mean differences (95% CI), number of contributing systematic reviews, and heterogeneity (I^2^) for mean change in weight and BMI, by intervention type and follow-up duration.

Intervention type	Follow-up (months)	No. of SRs	Pooled mean difference (95% CI)	I^2^ (%)
**Mean weight change (kg)**				
Low-calorie diet	<3	7	−2.64 (−2.94 to −2.34) [[13], [14], [15], [16]]	98.9
Low-calorie diet	3–6	11	−1.69 (−1.93 to −1.45) [[13], [14], [15],[17], [18], [19], [20]]	94.9
Low-calorie diet	6–12	20	−1.41 (−1.65 to −1.17) [[13], [14], [15],^18^,[20], [21], [22]]	83.6
Low-calorie diet	>12	7	−1.72 (−1.96 to −1.48) [13,20,23,24]	85.6
Intermittent fasting	<3	11	−0.66 (−0.77 to −0.55) [[25], [26], [27], [28], [29], [30], [31], [32]]	90.0
Intermittent fasting	3–6	8	−1.50 (−1.80 to −1.20) [28,[33], [34], [35]]	76.8
Intermittent fasting	6–12	19	−1.87 (−2.06 to −1.68) [28,33,[36], [37], [38], [39], [40]]	70.2
Intermittent fasting	>12	1	−0.39 (−2.07 to 1.29) [37]	0
High-protein diet	3–6	1	−0.30 (−1.15 to 0.55) [41]	0
High-protein diet	6–12	2	−1.28 (−1.94 to −0.62) [41]	53.0
High-protein diet	>12	2	−1.08 (−1.37 to −0.79) [42,43]	93.0
Other dietary	<3	11	−0.55 (−0.68 to −0.42) [[44], [45], [46], [47], [48], [49], [50]]	74.7
Other dietary	3–6	12	−0.20 (−0.31 to −0.09) [41,44,47,[51], [52], [53], [54], [55], [56], [57], [58]]	74.3
Other dietary	6–12	5	−1.08 (−1.40 to −0.76) [41,[59], [60], [61]]	28.2
Other dietary	>12	5	−0.16 (−0.32 to 0.00) [10,42,43,62,63]	95.8
Physical activity	<3	8	−0.19 (−0.32 to −0.06) [[64], [65], [66], [67], [68]]	85.4
Physical activity	3–6	10	−0.49 (−0.56 to −0.42) [65,[69], [70], [71], [72]]	89.2
Physical activity	6–12	9	−0.77 (−0.97 to −0.57) [[73], [74], [75], [76]]	61.8
Physical activity	>12	1	−1.23 (−2.03 to −0.43) [77]	0
Lifestyle	3–6	15	−0.49 (−0.61 to −0.37) [[78], [79], [80], [81], [82], [83], [84], [85], [86]]	87.7
Lifestyle	6–12	14	−0.41 (−0.52 to −0.30) [79,80,82,83,85,[87], [88], [89], [90]]	90.0
Lifestyle	>12	10	−1.46 (−1.76 to −1.16) [79,82,88,[91], [92], [93], [94]]	71.1
Diet + physical activity	<3	5	−1.82 (−2.18 to −1.46) [27,95]	85.9
Diet + physical activity	3–6	4	−1.60 (−2.10 to −1.10) [[95], [96], [97]]	91.5
Diet + physical activity	6–12	2	−0.99 (−1.62 to −0.36) [97,98]	96.5
Diet + physical activity	>12	1	−3.68 (−5.47 to −1.89) [99]	0
Diet + physical activity + lifestyle	3–6	2	−0.40 (−0.61 to −0.19) [100]	90.2
Diet + physical activity + lifestyle	6–12	5	−1.48 (−2.00 to −0.96) [101]	0
Diet + physical activity + lifestyle	>12	1	−0.37 (−0.54 to −0.20) [100]	0
**Mean BMI change (kg/m^2^)**				
Low-calorie diet	<3	2	−0.62 (−0.84 to −0.40) [15]	87.0
Low-calorie diet	3–6	1	−1.13 (−2.39 to 0.13) [15]	0
Low-calorie diet	6–12	5	−1.21 (−1.45 to −0.97) [15,22]	76.7
Low-calorie diet	>12	1	−0.50 (−0.65 to −0.35) [19]	0
Intermittent fasting	<3	6	−0.76 (−0.91 to −0.61) [25,26,31]	10.0
Intermittent fasting	6–12	1	−0.32 (−0.80 to 0.16) [39]	0
Other dietary	<3	8	−0.22 (−0.29 to −0.15) [[44], [45], [46], [47], [48]]	79.8
Other dietary	3–6	5	−0.21 (−0.36 to −0.06) [44,47,54,56,57]	34.9
Other dietary	6–12	3	−0.34 (−0.54 to −0.14) [[59], [60], [61]]	0.0
Physical activity	<3	4	−0.02 (−0.17 to 0.13) [65,67,68]	77.7
Physical activity	3–6	10	−0.46 (−0.59 to −0.33) [65,69,72,102]	72.3
Physical activity	6–12	6	0.02 (−0.01 to 0.05) [[73], [74], [75],^103^]	88.1
Physical activity	>12	2	−0.36 (−0.52 to −0.20) [66,77]	2.4
Lifestyle	<3	1	0.93 (−0.43 to 2.29) [83]	0
Lifestyle	3–6	6	−0.41 (−0.57 to −0.25) [78,[81], [82], [83],^86^]	5.0
Lifestyle	6–12	2	−0.13 (−0.47 to 0.21) [82]	51.3
Lifestyle	>12	2	0.09 (−1.10 to 0.18) [104,105]	84.1
Diet + physical activity	<3	2	−1.22 (−2.31 to −0.13) [106]	42.8
Diet + physical activity	>12	1	−0.64 (−1.10 to −0.18) [99]	0

Values are pooled mean differences (95% CI) from a random-effects model with REML estimation. A negative value favors the intervention.

BMI, body mass index; CI, confidence interval; MD, mean difference; REML, restricted maximum likelihood; SR, systematic review.

I^2^ is 0 where only a single review contributes to a stratum. Estimates with high I^2^ should be read as directional rather than precise.

BMI followed a similar pattern across 70 comparisons (Fig. 3). The greatest reduction was with combined diet and physical activity at less than 3 months (−1.22 kg/m^2^, 95% CI −2.31 to −0.13), and low-calorie diets reduced BMI by −0.50 to −1.21 kg/m^2^ across strata; reductions with physical activity and lifestyle interventions were small and frequently non-significant.

Among glycemic outcomes (Fig. 4), the largest improvements in fasting blood glucose and insulin resistance occurred with combined diet and physical activity beyond 12 months (−7.32 mg/dL, 95% CI −9.82 to −4.82; HOMA-IR −0.90, 95% CI −1.22 to −0.58). These pooled fasting blood glucose estimates are exploratory and descriptive only and should not be read as absolute mg/dL changes. Other dietary interventions reduced fasting glucose by −2.83 to −4.91 mg/dL in the short to medium term, and HbA1c reductions were modest, reaching −0.43% with low-calorie diets beyond 12 months. Effects on individual lipid fractions were inconsistent and largely non-significant across triglycerides, LDL, and HDL (Fig. 5). Isolated significant reductions in total cholesterol and triglycerides were observed, the largest being for total cholesterol with low-calorie diets beyond 12 months (−17.95 mg/dL, 95% CI −23.46 to −12.44); however, these derived from single reviews and, because contributing reviews reported these outcomes on differing scales, should be regarded as exploratory and descriptive only and not as absolute mg/dL changes (Table 2). For blood pressure (Fig. 6), physical activity produced the greatest systolic reduction (−6.10 mmHg, 95% CI −8.07 to −4.13 at 6–12 months) and intermittent fasting the greatest diastolic reduction (−3.30 mmHg, 95% CI −5.47 to −1.13 at <3 months).

**Table 2:** Pooled mean differences (95% CI), number of contributing systematic reviews, and heterogeneity (I^2^) for glycemic parameters, lipid parameters, and blood pressure, by intervention type and follow-up duration.

Intervention type	Follow-up (months)	No. of SRs	Pooled mean difference (95% CI)	I^2^ (%)
**Fasting blood glucose (mg/dL)**^a^				
Low-calorie diet	3–6	1	−0.99 (−5.54 to 3.56) [34]	0
Low-calorie diet	>12	2	−0.06 (−0.14 to 0.02) [13,107]	98.5
Intermittent fasting	<3	1	−3.36 (−9.02 to 2.30) [25]	0
Intermittent fasting	>12	2	−0.25 (−0.51 to 0.01) [43]	0
Other dietary	<3	1	−2.83 (−4.60 to −1.08) [46]	0
Other dietary	3–6	1	−4.91 (−8.29 to −1.53) [108]	0
Other dietary	6–12	1	−0.06 (−0.15 to 0.03) [62]	0
Physical activity	<3	1	−0.67 (−1.07 to −0.27) [67]	0
Lifestyle	3–6	1	−0.03 (−0.10 to 0.04) [93]	0
Diet + physical activity	<3	1	−1.92 (−4.53 to 0.69) [109]	0
Diet + physical activity	>12	1	−7.32 (−9.82 to −4.82) [99]	0
**HbA1c (%)**				
Low-calorie diet	3–6	1	−0.05 (−0.14 to 0.04) [34]	0
Low-calorie diet	>12	1	−0.43 (−0.70 to −0.16) [13]	0
Intermittent fasting	<3	1	−0.64 (−2.04 to 0.76) [25]	0
Intermittent fasting	>12	2	−0.48 (−0.69 to −0.27) [43]	61.4
Other dietary	<3	1	−0.17 (−0.44 to 0.10) [46]	0
Other dietary	6–12	2	0.02 (−0.13 to 0.17) [60,110]	55.4
**HOMA-IR**				
Low-calorie diet	>12	3	−0.15 (−0.26 to −0.04) [13,34,107]	79.0
Intermittent fasting	>12	2	−0.19 (−0.42 to 0.04) [43]	81.0
Other dietary	<3	1	−0.52 (−0.84 to −0.20) [46]	0
Other dietary	6–12	2	−0.20 (−0.63 to 0.23) [60,110]	0.0
Diet + physical activity	<3	1	−0.57 (−0.83 to −0.31) [111]	0
Diet + physical activity	>12	1	−0.90 (−1.22 to −0.58) [99]	0
**Total cholesterol (mg/dL)**^a^				
Low-calorie diet	<3	1	0.42 (0.23–0.61) [14]	0
Low-calorie diet	3–6	1	0.12 (0.03–0.21) [14]	0
Low-calorie diet	6–12	2	0.11 (0.04–0.18) [14]	0
Low-calorie diet	>12	1	−17.95 (−23.46 to −12.44) [13]	0
Intermittent fasting	<3	1	−6.31 (−12.36 to −0.26) [25]	0
Other dietary	<3	1	−11.81 (−19.65 to −3.97) [46]	0
Other dietary	3–6	1	−8.35 (−14.01 to −2.69) [108]	0
Physical activity	<3	1	−0.42 (−0.78 to −0.06) [67]	0
Physical activity	3–6	2	0.14 (0.01–0.27) [112,113]	82.5
Lifestyle	<3	1	−0.07 (−0.28 to 0.14) [79]	0
Lifestyle	6–12	1	−0.23 (−0.39 to −0.07) [79]	0
Lifestyle	>12	1	−0.17 (−0.29 to −0.05) [79]	0
Diet + physical activity	>12	1	−6.30 (−9.58 to −3.02) [99]	0
**Triglycerides (mg/dL)**^a^				
Low-calorie diet	<3	1	−0.26 (−0.34 to −0.18) [14]	0
Low-calorie diet	3–6	3	−0.15 (−0.23 to −0.07) [14,18]	95.0
Low-calorie diet	6–12	3	−0.07 (−0.12 to −0.02) [14,17]	93.4
Low-calorie diet	>12	3	−0.13 (−0.16 to −0.10) [13,107]	98.2
Intermittent fasting	<3	2	−0.32 (−0.49 to −0.15) [25,26]	78.5
Other dietary	<3	1	−10.31 (−20.00 to −0.62) [46]	0
Other dietary	3–6	1	−13.37 (−24.01 to −2.73) [108]	0
Physical activity	<3	1	−0.08 (−0.44 to 0.28) [67]	0
Physical activity	3–6	2	0.01 (−0.07 to 0.09) [112,113]	91.5
Diet + physical activity	<3	1	−0.06 (−0.27 to 0.15) [79]	0
Diet + physical activity	6–12	1	−13.38 (−21.22 to −5.54) [109]	0
Diet + physical activity	>12	2	−0.22 (−0.35 to −0.09) [79,99]	99.8
**LDL cholesterol (mg/dL)**^a^				
Low-calorie diet	<3	1	0.39 (0.26–0.52) [14]	0
Low-calorie diet	3–6	1	0.14 (0.06–0.22) [14]	0
Low-calorie diet	6–12	2	0.07 (0.03–0.11) [14]	0
Low-calorie diet	>12	2	0.10 (0.06–0.14) [13]	92.5
Intermittent fasting	<3	1	−5.44 (−10.74 to −0.14) [25]	0
Other dietary	<3	1	−8.01 (−15.78 to −0.24) [46]	0
Other dietary	3–6	1	−9.47 (−13.74 to −5.20) [108]	0
Physical activity	3–6	2	0.03 (−0.03 to 0.09) [112,113]	95.7
Diet + physical activity	<3	2	−0.09 (−0.23 to 0.05) [79,109]	79.8
Diet + physical activity	6–12	1	−8.52 (−11.71 to −5.33) [109]	0
Diet + physical activity	>12	1	−0.17 (−0.27 to −0.07) [79]	0
**HDL cholesterol (mg/dL)**^a^				
Low-calorie diet	<3	1	0.12 (0.09–0.15) [14]	0
Low-calorie diet	3–6	3	0.07 (0.05–0.09) [14,18]	92.3
Low-calorie diet	6–12	5	0.05 (0.03–0.07) [14,18]	90.4
Low-calorie diet	>12	3	0.06 (0.05–0.07) [13,107]	73.7
Intermittent fasting	<3	1	−0.89 (−4.54 to 2.76) [25]	0
Other dietary	3–6	1	−0.25 (−0.98 to 0.48) [108]	0
Physical activity	3–6	1	0.04 (0.02–0.06) [112]	0
Diet + physical activity	<3	1	0.02 (−0.06 to 0.10) [79]	0
Diet + physical activity	3–6	1	−0.22 (−0.49 to 0.05) [113]	0
Diet + physical activity	6–12	1	0.01 (−0.04 to 0.06) [79]	0
Diet + physical activity	>12	2	0.02 (−0.03 to 0.07) [99]	87.3
**Systolic blood pressure (mmHg)**				
Low-calorie diet	>12	1	−4.62 (−6.41 to −2.83) [114]	0
Intermittent fasting	<3	1	−1.51 (−3.46 to 0.44) [25]	0
Other dietary	3–6	1	−2.52 (−4.16 to −0.88) [108]	0
Other dietary	6–12	1	−2.44 (−5.20 to 0.32) [60]	0
Other dietary	>12	1	−1.34 (−2.00 to −0.68) [62]	0
Physical activity	3–6	1	−0.49 (−2.29 to 1.31) [113]	0
Physical activity	6–12	1	−6.10 (−8.07 to −4.13) [115]	0
Lifestyle	>12	2	−4.64 (−5.91 to −3.37) [80]	0
**Diastolic blood pressure (mmHg)**				
Low-calorie diet	>12	2	−2.96 (−4.08 to −1.84) [107,114]	0
Intermittent fasting	<3	1	−3.30 (−5.47 to −1.13) [25]	0
Other dietary	3–6	1	−1.58 (−2.54 to −0.62) [108]	0
Other dietary	6–12	1	−1.84 (−4.07 to 0.39) [60]	0
Other dietary	>12	1	−0.81 (−1.30 to −0.32) [62]	0
Physical activity	3–6	1	−0.63 (−1.67 to 0.41) [112]	0
Physical activity	6–12	1	−2.88 (−3.95 to −1.81) [115]	0
Lifestyle	>12	2	−0.71 (−1.61 to 0.19) [80]	0

Values are pooled mean differences (95% CI) from a random-effects model with REML estimation. A negative value favors the intervention for all outcomes except HDL cholesterol, for which a positive value is favorable.

CI, confidence interval; HOMA-IR, homeostatic model assessment of insulin resistance; HDL, high-density lipoprotein; LDL, low-density lipoprotein; MD, mean difference; REML, restricted maximum likelihood; SR, systematic review.

I^2^ is 0 where only a single review contributes to a stratum. Estimates with high I^2^ should be read as directional.

a *For fasting blood glucose and the four lipid outcomes (total cholesterol, triglycerides, LDL cholesterol, and HDL cholesterol), contributing reviews reported effects on differing scales- standardized mean differences alongside unstandardized mean differences in mg/dL. Pooled estimates for these five outcomes therefore combine values that are not on a common scale; they are presented as exploratory and descriptive only and should not be interpreted as absolute (mg/dL) changes. Where a single review dominates a stratum, the corresponding mg/dL estimate (e.g. total cholesterol beyond* 12mo*) derives from that review alone*.

Statistical heterogeneity was high across most pooled estimates (I^2^ commonly 70–99%), reflecting variation in intervention intensity, delivery, populations, and outcome measurement; pooled estimates should therefore be read as directional rather than precise. Across the three prespecified sensitivity analyses exclusion of small (≤100 participants), leave-one-out, and a fixed-effects model the direction and significance of the primary findings were unchanged.

## 4 Discussion

This umbrella review of 112 systematic reviews and meta-analyses demonstrates that all major non-pharmacological intervention categories produce statistically significant, modest reductions in body weight and BMI in adults with overweight and obesity, with effects generally attenuating over extended follow-up reflecting the persistent challenge of long-term adherence.

Low-calorie diets produced the largest short-term weight reductions (−2.64 kg at ≤3 months), with progressive attenuation attributable to adaptive metabolic responses. Intermittent fasting yielded consistent short-to-medium-term benefits but no significant effect beyond 12 months (−0.39 kg; 95% CI: −2.07, 1.29), consistent with evidence showing no sustained superiority over continuous energy restriction. High-protein diets demonstrated sustained reductions at 6–12 months (−1.28 kg) and beyond 12 months (−1.08 kg), A pattern commonly attributed to the higher thermic effect of protein and its role in preserving lean mass during energy restriction [42]. Other dietary approaches produced smaller, short-lived effects without overall caloric restriction.

Physical activity alone produced modest but significant weight reductions across all durations (−0.19 to −1.23 kg), attenuated by energy compensation behaviors, though its value extends to independent improvements in cardiorespiratory fitness, insulin sensitivity, and cardiovascular risk. Lifestyle interventions showed progressively increasing reductions with longer follow-up (−0.49 kg at 3–6 months; −1.46 kg beyond 12 months), highlighting the scalability of behavioral and mHealth approaches at population level.

Combined diet and physical activity produced the largest weight reduction of any stratum (−3.68 kg; 95% CI: −5.47, −1.89 beyond 12 months), with exercise preserving lean mass and attenuating diet-induced metabolic suppression. Fully multicomponent programs (diet + physical activity + lifestyle) demonstrated moderate 6–12 month reductions (−1.48 kg), with self-monitoring, goal setting, and relapse prevention likely sustaining effects relative to dietary approaches alone.

Combined diet and physical activity yielded the largest sustained improvements in fasting blood glucose (−7.32 mg/dL; an exploratory, descriptive estimate that is not an absolute mg/dL change) and HOMA-IR (−0.90) beyond 12 months. HbA1c reductions were modest but clinically meaningful in prediabetes and early type 2 diabetes contexts. Total cholesterol reduction was greatest with low-calorie diets beyond 12 months (−17.95 mg/dL; an exploratory, descriptive estimate that is not an absolute mg/dL change), though based on a single contributing review. Individual lipid fractions were frequently non-significant, likely reflecting opposing macronutrient-specific effects across heterogeneous protocols. Physical activity produced the greatest systolic blood pressure reduction at 6–12 months (−6.10 mmHg), and intermittent fasting the greatest diastolic reduction at <3 months (−3.30 mmHg).

High heterogeneity (I^2^ commonly 70–99%) reflects genuine variation in intervention intensity, delivery modality, participant characteristics, and outcome methodology; pooled estimates should be interpreted as directional indicators. Sensitivity analyses confirmed directional robustness.

### 4.1 Strengths

Strengths include comprehensive scope (112 reviews, 424 comparisons, and 507,460 participant-observations, corresponding to 2332 unique randomized trials and an estimated 248,187 unique participants across more than 50 countries), prospective PROSPERO registration, PRISMA 2020 adherence, dual AMSTAR 2 and GRADE appraisal, and a low overlap between reviews (corrected covered area 0.20%).

### 4.2 Limitations

Several limitations temper these findings. First, the primary search was restricted to a single database (PubMed), although backward and forward citation tracking, and the fact that the included reviews had themselves drawn on at least six databases, partially offset this. Second, methodological quality was variable, 48 reviews (43%) were rated low and 29 (26%) critically low on AMSTAR 2 and statistical heterogeneity was high across most pooled estimates (I^2^ commonly 70–99%), so pooled values should be read as directional rather than precise. Third, the evidence was geographically clustered in high-income and upper-middle-income settings, with only 2% of reviews drawing on any low-income-country trial data, limiting generalizability to low-and middle-income countries. Fourth, for fasting blood glucose and the four lipid outcomes, contributing reviews reported effects on differing scales (standardized mean differences alongside unstandardized mean differences in mg/dL), the corresponding pooled estimates therefore mix scales and are exploratory and descriptive only, and must not be interpreted as absolute mg/dL changes.

Fifth, meal-replacement interventions, a substantial component of contemporary obesity treatment, were not analyzed as a discrete category. Some meal-replacement and very-low-energy formula trials were captured within the low-calorie and very-low-energy diet clusters. Finally, as an umbrella review, this synthesis inherits the primary-study and review-level biases of the underlying literature and cannot establish causal relationships or definitive comparative superiority between modalities. Priorities for future research include adequately powered, long-term (beyond 12 months) multicomponent randomized trials, greater representation of low- and middle-income countries; dedicated synthesis of meal-replacement and of lifestyle approaches used alongside pharmacotherapy and investigation of the mechanisms underlying effect attenuation and the comparative effectiveness of specific dietary patterns, exercise modalities, and digital behavior-change components.

### 4.3 Clinical and public health implications

Even modest population-level weight reductions of 1–2 kg translate to clinically meaningful decreases in type 2 diabetes, hypertension, and cardiovascular disease incidence. Notably, cardiometabolic benefits are at least partially independent of absolute weight loss magnitude, directly relevant for patients unable to achieve conventional 5–10% weight loss targets.

## 5 Conclusion

Non-pharmacological interventions are often associated with modest reductions in body weight and body mass index and general improvement in cardiometabolic risk markers. Combining healthful nutrition, increased physical activity, and behavioral support appeared to provide the most consistent overall benefit, although high heterogeneity, variable review quality, and exploratory cardiometabolic analyses limit the precision and certainty of these estimates. While the treatment paradigm has shifted toward incorporating pharmacotherapy as a fundamental component of weight management, particularly in an era when more patients need or seek a reduction of 10% or greater to achieve meaningful health improvements, given the multiple comorbidities associated with excess adiposity and the higher average body mass index now seen in practice, these findings continue to acknowledge the important role and potential benefits of lifestyle interventions.

• All major non-pharmacological modalities produced modest reductions in body weight and body mass index, with combined diet, physical activity, and behavioral support giving the most consistent and sustained benefit.
• Cardiometabolic improvements were at least partly independent of the magnitude of weight lost, so lifestyle programs remain worthwhile even when 5–10% weight-loss targets are not reached, and can complement pharmacotherapy.
• High heterogeneity, variable review quality, and under-representation of low- and middle-income countries limit certainty; adequately powered, long-term multicomponent trials in these settings are a priority for future research.

## Ethics approval and consent

This umbrella review used only published, publicly available data from previously reported studies; no ethics committee approval or participant consent was required. Patients and the public were not involved in the design, conduct, or reporting of this review.

## CRediT authorship contribution statement

**Mahesh Kumar Mummadi:** Conceptualization, Methodology, Investigation, Formal analysis, Writing – original draft, Writing – review & editing. **Subbarao M. Gavaravarapu:** Investigation, Writing – original draft, Writing – review & editing. **Samarasimha N. Reddy:** Conceptualization, Methodology, Supervision, Investigation, Writing – original draft, Writing – review & editing, Data curation, Project administration. **Karthikeyan Ramanujam:** Formal analysis, Writing – review & editing. **Ritika Prakash:** Investigation, Formal analysis, Writing – original draft. **Sudhakar Reddy Vadde:** Writing – review & editing. **Raghavendra Pandurangi:** Writing – review & editing. **Priyanka G. Bansal:** Writing – review & editing. **Ranadip Chowdhury:** Writing – review & editing. **Bharati Kulkarni:** Writing – review & editing, Supervision. Dr Samarasimha N. Reddy directly accessed and verified the underlying data and had primary responsibility for the final content. All authors read and approved the final manuscript.

## Data availability

All data analyzed were extracted from the published systematic reviews listed in the reference list. The extraction dataset, the corrected covered area matrix, the code book, and the analysis code are available from the corresponding author on reasonable request from the date of publication.

## Transparency statement

The corresponding author affirms that this manuscript is an honest, accurate, and transparent account of the study being reported; that no important aspects of the study have been omitted; and that any discrepancies from the study as registered (PROSPERO CRD420251245029) have been explained.

## Declaration of generative AI and AI-assisted technologies in the writing process

During the preparation of this work the authors used Claude (Anthropic) in order to support writing, editing, and formatting tasks, including word-count reduction, grammar checking, and adherence to journal formatting guidelines. After using this tool, the authors reviewed and edited the content as needed and take full responsibility for the content of the publication. All scientific content, data interpretation, and conclusions were generated, verified, and approved exclusively by the authors.

## Funding

This study received no specific grant from any funding agency in the public, commercial, or not-for-profit sectors.

## Declaration of competing interest

The authors declare that they have no known competing financial interests or personal relationships that could have appeared to influence the work reported in this paper.
