# Supplementary material for: Effectiveness of dietary, physical activity, and lifestyle interventions on weight and cardiometabolic outcomes in adults with overweight or obesity: an umbrella review of 112 systematic reviews and meta-analyses from 50 countries
Source: Obes Pillars. 2026 Sep 1;20:100324. doi: 10.1016/j.obpill.2026.100324 (PMC13571594; doi:10.1016/j.obpill.2026.100324)
Supplement: Supplementary file 1 [file mmc1.docx]

**Supplemental Figure 1:**

**Forest Plots**

**Mean Weight Change (in kgs) associated with different intervention types**

**Mean Change in BMI (IN Kg/m^2^) associated with the different intervention types**

**Mean Fasting Blood Glucose Change (mg/dl) associated with the different intervention types**

**Mean Change in HbA1c (%) associated with the different intervention types**

**Mean Change in HOMA-IR associated with the different intervention types**

**Mean Change in total cholesterol (mg/dl) associated with the different intervention types**

**Mean Change in triglycerides (mg/dl) associated with the different intervention types**

**Mean Change in HDL (mg/dl) associated with the different intervention types**

**Mean Change in LDL (mg/dl) associated with the different intervention types**

**Mean Change in Systolic BP (mmHg) associated with the different intervention types**

**Mean Change in Diastolic BP (mmHg) associated with the different intervention types**
