# Supplementary material for: Effectiveness of dietary, physical activity, and lifestyle interventions on weight and cardiometabolic outcomes in adults with overweight or obesity: an umbrella review of 112 systematic reviews and meta-analyses from 50 countries
Source: Obes Pillars. 2026 Sep 1;20:100324. doi: 10.1016/j.obpill.2026.100324 (PMC13571594; doi:10.1016/j.obpill.2026.100324)
Supplement: Supplementary file 2 [file mmc2.docx]

**Supplemental Table 1: PUBMED search strategy**

| Search number | Query and Filters | Results |
| --- | --- | --- |
| 1 | (adult* OR "young adults" OR "middle-aged" OR elderly OR "older adults") AND (obesity OR overweight OR "adult obesity" OR "excess weight" OR "body fat" OR "body weight" OR BMI OR "body mass index" OR "central obesity" OR adiposity OR "abdominal obesity" OR "skinfold thickness" OR "Body Fat percentage") | 587,961 |
| 2 | ("diet" OR "nutrition" OR "dietary intervention" OR "dietary modification" OR "healthy diet" OR "balanced diet" OR "meal planning" OR "nutrition education" OR "diet counseling" OR "food intake" OR "diet quality" OR "diet supplementation" OR "HFSS consumption" OR "physical activity" OR "fitness program" OR exercise OR "aerobic exercise" OR "resistance training" OR "strength training" OR yoga OR sports OR "motor activity" OR "movement-based intervention" OR "sedentary behavior" OR "screen time reduction" OR sleep OR "sleep duration" OR "sleep hygiene" OR "stress" OR "stress management" OR mindfulness OR "emotional wellbeing" OR "mental wellbeing" OR "lifestyle intervention" OR "behavioral intervention" OR "healthy lifestyle" OR "behavior change" OR "motivational interviewing" OR "counseling" OR "self-monitoring" OR "goal setting" OR "digital intervention" OR "mobile app" OR "telehealth" OR "health coaching" OR "environmental modification" OR "community-based intervention" OR "family-based intervention" OR "school-based intervention" OR "workplace intervention" OR "public health intervention" OR "health promotion" OR "policy intervention" OR "education intervention" OR "social marketing" OR "health communication") | 4,158,168 |
| 3 | ("obesity prevention" OR "obesity management" OR "weight management" OR "weight reduction" OR "BMI reduction" OR "body composition" OR "adiposity reduction" OR "Body Fat percentage reduction" OR "prevention" OR "management" OR "treatment") | 10,231,356 |
| 4 | ("systematic review" OR "meta-analysis" OR "umbrella review" OR "review of reviews" OR "evidence synthesis") | 611,304 |
| 5 | ((((#1) AND (#2)) AND (#3)) AND (#4))  Free full text, Meta-Analysis, Systematic Review, from 2015 - 2025 | 1,550 |

(as of December 2025)
