# Supplementary material for: Effectiveness of dietary, physical activity, and lifestyle interventions on weight and cardiometabolic outcomes in adults with overweight or obesity: an umbrella review of 112 systematic reviews and meta-analyses from 50 countries
Source: Obes Pillars. 2026 Sep 1;20:100324. doi: 10.1016/j.obpill.2026.100324 (PMC13571594; doi:10.1016/j.obpill.2026.100324)
Supplement: Supplementary file 3 [file mmc3.docx]

**Supplemental Table 2: Assessment of the methodological quality of included systematic reviews using the AMSTAR-2.**

| S.NO | STUDY | AMSTAR 2 ITEMS | | | | | | | | | | | | | | | | SUMMARY SCORE |
| --- | --- | --- | --- | --- | --- | --- | --- | --- | --- | --- | --- | --- | --- | --- | --- | --- | --- | --- |
|  |  | **PICO** | **Protocol** | **Explanation**  **Eligibility criteria** | **Comprehensive literature search** | **Study selection in duplicate** | **Data extraction in duplicate** | **List of excluded studies** | **Description of included studies** | **Satisfactory Risk of**  **Bias assessment** | **Report funding of included studies** | **Statistical combination of results** | **Effect of Risk of Bias on meta-analysis** | **Account for Risk of Bias** | **Explanation for heterogeneity** | **Publication bias** | **Conflict of Interest** |  |
| 1 | Ahmad Binmahfoz, 2025 |  |  |  |  |  |  |  |  |  |  |  |  |  |  |  |  | **LOW** |
| 2 | Ahmad Jayedi, PhD, 2024 |  |  |  |  |  |  |  |  |  |  |  |  |  |  |  |  | **HIGH** |
| 3 | Alline M Beleigoli, 2019 |  |  |  |  |  |  |  |  |  |  |  |  |  |  |  |  | **CRITICALLY LOW** |
| 4 | Ana Marenco Kramer, 2023 |  |  |  |  |  |  |  |  |  |  |  |  |  |  |  |  | **LOW** |
| 5 | Angeliki Papadaki, 2020 |  |  |  |  |  |  |  |  |  |  |  |  |  |  |  |  | **HIGH** |
| 6 | Anne Karen Jenum, 2019 |  |  |  |  |  |  |  |  |  |  |  |  |  |  |  |  | **HIGH** |
| 7 | Arezoo Sadat Emrani, 2023 |  |  |  |  |  |  |  |  |  |  |  |  |  |  |  |  | **HIGH** |
| 8 | Armin Ebrahimzadeh, 2024 |  |  |  |  |  |  |  |  |  |  |  |  |  |  |  |  | **LOW** |
| 9 | Bahar Kucuk, 2022 |  |  |  |  |  |  |  |  |  |  |  |  |  |  |  |  | **CRITICALLY LOW** |
| 10 | Bingjie Wang, 2025 |  |  |  |  |  |  |  |  |  |  |  |  |  |  |  |  | **LOW** |
| 11 | Carlos Gomez-Garcia, 2025 |  |  |  |  |  |  |  |  |  |  |  |  |  |  |  |  | **CRITICALLY LOW** |
| 12 | Carmelo Pujia, 2025 |  |  |  |  |  |  |  |  |  |  |  |  |  |  |  |  | **LOW** |
| 13 | Carmen Varela, 2021 |  |  |  |  |  |  |  |  |  |  |  |  |  |  |  |  | **LOW** |
| 14 | Cate Bulmer, 2025 |  |  |  |  |  |  |  |  |  |  |  |  |  |  |  |  | **CRITICALLY LOW** |
| 15 | Celeste E Naude, 2022 |  |  |  |  |  |  |  |  |  |  |  |  |  |  |  |  | **HIGH** |
| 16 | Chaitong Churuangsuk, 2021 |  |  |  |  |  |  |  |  |  |  |  |  |  |  |  |  | **LOW** |
| 17 | Chanthawat Patikorn, 2021 |  |  |  |  |  |  |  |  |  |  |  |  |  |  |  |  | **HIGH** |
| 18 | Chenhan Ma, 2017 |  |  |  |  |  |  |  |  |  |  |  |  |  |  |  |  | **LOW** |
| 19 | Chenlin Gao, 2019 |  |  |  |  |  |  |  |  |  |  |  |  |  |  |  |  | **LOW** |
| 20 | Chenxi Hu, 2024 |  |  |  |  |  |  |  |  |  |  |  |  |  |  |  |  | **LOW** |
| 21 | Claire D Madigan, 2022 |  |  |  |  |  |  |  |  |  |  |  |  |  |  |  |  | **LOW** |
| 22 | D. Power, 2025 |  |  |  |  |  |  |  |  |  |  |  |  |  |  |  |  | **MODERATE** |
| 23 | Dr Deirdre K Tobias ScD, 2015 |  |  |  |  |  |  |  |  |  |  |  |  |  |  |  |  | **MODERATE** |
| 24 | Duc Tran Quang, 2025 |  |  |  |  |  |  |  |  |  |  |  |  |  |  |  |  | **LOW** |
| 25 | Ehrika Vanessa Almeida de Menezes, 2019 |  |  |  |  |  |  |  |  |  |  |  |  |  |  |  |  | **CRITICALLY LOW** |
| 26 | Evangelia Malakou, 2018 |  |  |  |  |  |  |  |  |  |  |  |  |  |  |  |  | **LOW** |
| 27 | Felipe da Fonseca Silva Couto, 2025 |  |  |  |  |  |  |  |  |  |  |  |  |  |  |  |  | **MODERATE** |
| 28 | Gabrielle Maston, 2019 |  |  |  |  |  |  |  |  |  |  |  |  |  |  |  |  | **CRITICALLY LOW** |
| 29 | Gina M Wren, 2024 |  |  |  |  |  |  |  |  |  |  |  |  |  |  |  |  | **CRITICALLY LOW** |
| 30 | Giovanna Muscogiuri, 2021 |  |  |  |  |  |  |  |  |  |  |  |  |  |  |  |  | **CRITICALLY LOW** |
| 31 | [Grainne O'Donoghue, 2020](https://onlinelibrary.wiley.com/authored-by/O%27Donoghue/Grainne) |  |  |  |  |  |  |  |  |  |  |  |  |  |  |  |  | **LOW** |
| 32 | Haifeng Zhu, 2023 |  |  |  |  |  |  |  |  |  |  |  |  |  |  |  |  | **MODERATE** |
| 33 | Hamidreza Raeisi-Dehkordi, 2019 |  |  |  |  |  |  |  |  |  |  |  |  |  |  |  |  | **HIGH** |
| 34 | Hamish A. Fernando, 2019 |  |  |  |  |  |  |  |  |  |  |  |  |  |  |  |  | **CRITICALLY LOW** |
| 35 | Han Shi Jocelyn Chew, 2022 |  |  |  |  |  |  |  |  |  |  |  |  |  |  |  |  | **LOW** |
| 36 | Han-Na Kim, 2019 |  |  |  |  |  |  |  |  |  |  |  |  |  |  |  |  | **CRITICALLY LOW** |
| 37 | Hao Qiu, 2025 |  |  |  |  |  |  |  |  |  |  |  |  |  |  |  |  | **MODERATE** |
| 38 | Harris Leanne; 2018 |  |  |  |  |  |  |  |  |  |  |  |  |  |  |  |  | **LOW** |
| 39 | Hossein Bahari, 2023 |  |  |  |  |  |  |  |  |  |  |  |  |  |  |  |  | **MODERATE** |
| 40 | Iolanda Cioffi, 2018 |  |  |  |  |  |  |  |  |  |  |  |  |  |  |  |  | **LOW** |
| 41 | Iván Cavero-Redondo, 2020 |  |  |  |  |  |  |  |  |  |  |  |  |  |  |  |  | **LOW** |
| 42 | J Eales, 2015 |  |  |  |  |  |  |  |  |  |  |  |  |  |  |  |  | **HIGH** |
| 43 | J. Salse-Batán, 2022 |  |  |  |  |  |  |  |  |  |  |  |  |  |  |  |  | **CRITICALLY LOW** |
| 44 | Jakub Morze, 2021 |  |  |  |  |  |  |  |  |  |  |  |  |  |  |  |  | **HIGH** |
| 45 | Jamie Hartmann-Boyce, 2022 |  |  |  |  |  |  |  |  |  |  |  |  |  |  |  |  | **LOW** |
| 46 | Jana Jabbour, 2022 |  |  |  |  |  |  |  |  |  |  |  |  |  |  |  |  | **LOW** |
| 47 | Jie Men, 2025 |  |  |  |  |  |  |  |  |  |  |  |  |  |  |  |  | **LOW** |
| 48 | Jinming Huang, 2024 |  |  |  |  |  |  |  |  |  |  |  |  |  |  |  |  | **LOW** |
| 49 | Joana M. Correia, 2021 |  |  |  |  |  |  |  |  |  |  |  |  |  |  |  |  | **LOW** |
| 50 | Jonathan P. Bonnet, 2020 |  |  |  |  |  |  |  |  |  |  |  |  |  |  |  |  | **HIGH** |
| 51 | Jonathan Sackner-Bernstein, 2015 |  |  |  |  |  |  |  |  |  |  |  |  |  |  |  |  | **CRITICALLY LOW** |
| 52 | Jong Han Choi, 2022 |  |  |  |  |  |  |  |  |  |  |  |  |  |  |  |  | **CRITICALLY LOW** |
| 53 | Ke Yao, 2024 |  |  |  |  |  |  |  |  |  |  |  |  |  |  |  |  | **CRITICALLY LOW** |
| 54 | Kevin C Maki, 2019 |  |  |  |  |  |  |  |  |  |  |  |  |  |  |  |  | **CRITICALLY LOW** |
| 55 | Ke-Wen Wan, 2024 |  |  |  |  |  |  |  |  |  |  |  |  |  |  |  |  | **HIGH** |
| 56 | Kuo-Jen Hsu, 2019 |  |  |  |  |  |  |  |  |  |  |  |  |  |  |  |  | **CRITICALLY LOW** |
| 57 | Kyoung-Bae Kim, 2019 |  |  |  |  |  |  |  |  |  |  |  |  |  |  |  |  | **CRITICALLY LOW** |
| 58 | L. Harris, 2017 |  |  |  |  |  |  |  |  |  |  |  |  |  |  |  |  | **LOW** |
| 59 | Laura Chiavaroli, 2018 |  |  |  |  |  |  |  |  |  |  |  |  |  |  |  |  | **HIGH** |
| 60 | Lauren Ball, 2021 |  |  |  |  |  |  |  |  |  |  |  |  |  |  |  |  | **LOW** |
| 61 | Lee Hooper, 2020 |  |  |  |  |  |  |  |  |  |  |  |  |  |  |  |  | **HIGH** |
| 62 | [Leila Jahangiry, 2021](https://jhpn.biomedcentral.com/articles/10.1186/s41043-021-00240-3#auth-Leila-Jahangiry-Aff1) |  |  |  |  |  |  |  |  |  |  |  |  |  |  |  |  | **CRITICALLY LOW** |
| 63 | Leona Yuen-Ling Leung, 2025 |  |  |  |  |  |  |  |  |  |  |  |  |  |  |  |  | **LOW** |
| 64 | Liana L Guarneiri, 2021 |  |  |  |  |  |  |  |  |  |  |  |  |  |  |  |  | **LOW** |
| 65 | Lifu Lei, 2022 |  |  |  |  |  |  |  |  |  |  |  |  |  |  |  |  | **CRITICALLY LOW** |
| 66 | Long Ge, 2020 |  |  |  |  |  |  |  |  |  |  |  |  |  |  |  |  | **CRITICALLY LOW** |
| 67 | Maite M. Schroor, 2023 |  |  |  |  |  |  |  |  |  |  |  |  |  |  |  |  | **MODERATE** |
| 68 | Manuela Chiavarini, 2025 |  |  |  |  |  |  |  |  |  |  |  |  |  |  |  |  | **LOW** |
| 69 | Maria-Inti Metzendorf, 2024 |  |  |  |  |  |  |  |  |  |  |  |  |  |  |  |  | **HIGH** |
| 70 | Marlene A. van Baak, 2019 |  |  |  |  |  |  |  |  |  |  |  |  |  |  |  |  | **MODERATE** |
| 71 | Matthew Snelson, 2019 |  |  |  |  |  |  |  |  |  |  |  |  |  |  |  |  | **HIGH** |
| 72 | Michelle Headland, 2016 |  |  |  |  |  |  |  |  |  |  |  |  |  |  |  |  | **CRITICALLY LOW** |
| 73 | Min Zhuang, 2022 |  |  |  |  |  |  |  |  |  |  |  |  |  |  |  |  | **CRITICALLY LOW** |
| 74 | Mohamad Motevalli, 2025 |  |  |  |  |  |  |  |  |  |  |  |  |  |  |  |  | **CRITICALLY LOW** |
| 75 | Mohammadreza Moradi Baniasadi, 2025 |  |  |  |  |  |  |  |  |  |  |  |  |  |  |  |  | **HIGH** |
| 76 | Mona Kholdebarin, 2024 |  |  |  |  |  |  |  |  |  |  |  |  |  |  |  |  | **HIGH** |
| 77 | Mousa Khalafi, 2025 |  |  |  |  |  |  |  |  |  |  |  |  |  |  |  |  | **LOW** |
| 78 | Néma D. McGlynn, 2022 |  |  |  |  |  |  |  |  |  |  |  |  |  |  |  |  | **HIGH** |
| 79 | Nerys M. Astbury, 2019 |  |  |  |  |  |  |  |  |  |  |  |  |  |  |  |  | **HIGH** |
| 80 | Nia Coupe, 2019 |  |  |  |  |  |  |  |  |  |  |  |  |  |  |  |  | **CRITICALLY LOW** |
| 81 | Omid Sadeghi, 2020 |  |  |  |  |  |  |  |  |  |  |  |  |  |  |  |  | **LOW** |
| 82 | Paul Blazey, 2023 |  |  |  |  |  |  |  |  |  |  |  |  |  |  |  |  | **HIGH** |
| 83 | Qiao-Yi Chen, 2024 |  |  |  |  |  |  |  |  |  |  |  |  |  |  |  |  | **LOW** |
| 84 | Ran Li, 2025 |  |  |  |  |  |  |  |  |  |  |  |  |  |  |  |  | **LOW** |
| 85 | Rubén Fernández-Rodríguez, 2021 |  |  |  |  |  |  |  |  |  |  |  |  |  |  |  |  | **HIGH** |
| 86 | Shreya Chawla, 2020 |  |  |  |  |  |  |  |  |  |  |  |  |  |  |  |  | **LOW** |
| 87 | Silvia Sanca-Valeriano, 2023 |  |  |  |  |  |  |  |  |  |  |  |  |  |  |  |  | **LOW** |
| 88 | Siew Min Ang, 2021 |  |  |  |  |  |  |  |  |  |  |  |  |  |  |  |  | **LOW** |
| 89 | Simone Perna, 2020 |  |  |  |  |  |  |  |  |  |  |  |  |  |  |  |  | **LOW** |
| 90 | Stephen D. Anton, 2017 |  |  |  |  |  |  |  |  |  |  |  |  |  |  |  |  | **CRITICALLY LOW** |
| 91 | Taibah Almesbehi, 2022 |  |  |  |  |  |  |  |  |  |  |  |  |  |  |  |  | **HIGH** |
| 92 | Tainah O. P. Arisi, 2024 |  |  |  |  |  |  |  |  |  |  |  |  |  |  |  |  | **LOW** |
| 93 | Tanja Črešnovar, 2023 |  |  |  |  |  |  |  |  |  |  |  |  |  |  |  |  | **LOW** |
| 94 | Thea Toft Hansen, 2021 |  |  |  |  |  |  |  |  |  |  |  |  |  |  |  |  | **MODERATE** |
| 95 | Theogene Habumugisha, 2023 |  |  |  |  |  |  |  |  |  |  |  |  |  |  |  |  | **LOW** |
| 96 | Thomas Semlitsch, 2021 |  |  |  |  |  |  |  |  |  |  |  |  |  |  |  |  | **HIGH** |
| 97 | Thompson Sharon V, 2017 |  |  |  |  |  |  |  |  |  |  |  |  |  |  |  |  | **LOW** |
| 98 | Víctor Siles-Guerrero,2024 |  |  |  |  |  |  |  |  |  |  |  |  |  |  |  |  | **CRITICALLY LOW** |
| 99 | Weihao Wang, 2020 |  |  |  |  |  |  |  |  |  |  |  |  |  |  |  |  | **LOW** |
| 100 | Welma Stonehouse, 2016 |  |  |  |  |  |  |  |  |  |  |  |  |  |  |  |  | **LOW** |
| 101 | Yeo Jin Choi, 2020 |  |  |  |  |  |  |  |  |  |  |  |  |  |  |  |  | **CRITICALLY LOW** |
| 102 | Yi Wang, 2021 |  |  |  |  |  |  |  |  |  |  |  |  |  |  |  |  | **LOW** |
| 103 | Yixun Xie, 2024 |  |  |  |  |  |  |  |  |  |  |  |  |  |  |  |  | **MODERATE** |
| 104 | Yongchao Xie, 2024 |  |  |  |  |  |  |  |  |  |  |  |  |  |  |  |  | **LOW** |
| 105 | Yue Lun Loh, 2023 |  |  |  |  |  |  |  |  |  |  |  |  |  |  |  |  | **MODERATE** |
| 106 | Zhicheng Guo, 2022 |  |  |  |  |  |  |  |  |  |  |  |  |  |  |  |  | **LOW** |
| 107 | Zhila Semnani-Azad, 2025 |  |  |  |  |  |  |  |  |  |  |  |  |  |  |  |  | **HIGH** |
| 108 | [Zhongxu Hu, 2025](https://loop.frontiersin.org/people/3031138) |  |  |  |  |  |  |  |  |  |  |  |  |  |  |  |  | **CRITICALLY LOW** |
| 109 | Zi-Han Dai,2025 |  |  |  |  |  |  |  |  |  |  |  |  |  |  |  |  | **LOW** |
| 110 | Zihan Dai, 2024 |  |  |  |  |  |  |  |  |  |  |  |  |  |  |  |  | **LOW** |
| 111 | Ziyan Ding, 2025 |  |  |  |  |  |  |  |  |  |  |  |  |  |  |  |  | **LOW** |
| 112 | Jong Han Choi, 2022 |  |  |  |  |  |  |  |  |  |  |  |  |  |  |  |  | **CRITICALLY LOW** |

**NOTE 1:** = YES = NO = PARTIAL YES

**NOTE 2:** selected AMSTAR 2 critical domain, (1) Protocol registered before commencement of the review, (2) Adequacy of the literature search,

(3) Justification for excluding individual studies, (4) Risk of bias assessment of included studies, (5) Appropriateness of meta-

Analytical methods, (6) Consideration of risk of bias when interpreting results, (7) Assessment of publication bias.

**NOTE 3:** Reviews were rated as **high confidence** when they had no or only one non-critical weakness, Reviews with **moderate confidence** had more than one non-critical weakness but no critical flaws. Reviews were rated as **low confidence** when at least one critical flaw was identified, with or without additional non-critical weaknesses, **critically low confidence** contained more than one critical flaw, irrespective of non-critical weaknesses.

**NOTE 4 For AMSTAR-2:**

| Item | Yes | Partial Yes | No |
| --- | --- | --- | --- |
| 1. PICO components | Population, intervention, comparator, and outcome clearly defined | – | One or more PICO elements missing |
| 2. Protocol registered | Protocol registered (e.g., PROSPERO) + deviations justified | Written protocol mentioned but not registered | No protocol reported |
| 3. Study design justification | Clear justification for included study designs | – | No justification provided |
| 4. Literature search | ≥2 databases + grey literature/registries + references checked | ≥2 databases + search strategy reported | Inadequate or unclear search |
| 5. Study selection in duplicate | Two reviewers independently screened | – | Single reviewer or unclear |
| 6. Data extraction in duplicate | Two reviewers independently extracted data | – | Single reviewer or unclear |
| 7. Excluded studies list | Full list + reasons for exclusion | List provided without reasons | No list provided |
| 8. Description of studies | Detailed population, intervention, comparator, outcomes, setting, follow-up | Basic description of PICO and design | Inadequate description |
| 9. Risk of bias assessment | Appropriate RoB tool used with all key domains | Some RoB domains assessed | No RoB assessment |
| 10. Funding of included studies | Funding sources reported or searched for | – | Not reported |
| 11. Meta-analysis methods | Appropriate statistical methods + heterogeneity explored | – | Inappropriate or unclear methods |
| 12. Impact of RoB on synthesis | Sensitivity/subgroup analysis by RoB | – | Not assessed |
| 13. RoB considered in interpretation | Results discussed in light of RoB | – | RoB ignored in discussion |
| 14. Heterogeneity explained | Heterogeneity explored and discussed | – | Not explored |
| 15. Publication bias | Formal tests or funnel plot + discussion | – | Not assessed |
| 16. Conflict of interest | COI and funding reported | – | Not reported |
