# Supplementary material for: Effectiveness of dietary, physical activity, and lifestyle interventions on weight and cardiometabolic outcomes in adults with overweight or obesity: an umbrella review of 112 systematic reviews and meta-analyses from 50 countries
Source: Obes Pillars. 2026 Sep 1;20:100324. doi: 10.1016/j.obpill.2026.100324 (PMC13571594; doi:10.1016/j.obpill.2026.100324)
Supplement: Supplementary file 4 [file mmc4.docx]

**Supplemental Table 3: GRADE summary of evidence for all 11 outcome domains**

| Outcome | Participants | Risk of Bias | Inconsistency | Indirectness | Imprecision | Publication Bias | Certainty (GRADE) |
| --- | --- | --- | --- | --- | --- | --- | --- |
| Body weight (kg) | 159,007 | Low | 95.6 | Same population | CI NARROW, DOES NOT CROSS 0 | No | ⊕⊕⊕◯ Moderate |
| BMI (kg/m²) | 79,228 | Low | 95.3 | Same population | CI NARROW, DOES NOT CROSS 0 | No | ⊕⊕⊕◯ Moderate |
| HOMA-IR | 1,833 | Low | 64.2 | Same population | CI NARROW, DOES NOT CROSS 0 | No | ⊕⊕⊕◯ Moderate |
| HbA1c (%) | 7,988 | Low | 79.6 | Same population | CI NARROW, DOES NOT CROSS 0 | No | ⊕⊕⊕◯ Moderate |
| Fasting glucose (mg/dL) | 1,183 | Low | 88.3 | Same population | CI NARROW, DOES NOT CROSS 0 | No | ⊕⊕⊕◯ Moderate |
| Total cholesterol (mg/dL) | 5,193 | Low | 92 | Same population | CI NARROW, DOES NOT CROSS 0 | No | ⊕⊕⊕◯ Moderate |
| Triglycerides (mg/dL) | 10,031 | Low | 78.5 | Same population | CROSSES 0 | No | ⊕⊕◯◯ Low |
| HDL cholesterol (mg/dL) | 9,478 | Low | 83.4 | Same population | CROSSES 0 | No | ⊕⊕◯◯ Low |
| LDL cholesterol (mg/dL) | 7,610 | Low | 85.2 | Same population | FAIRLY WIDE | No | ⊕⊕◯◯ Low |
| Systolic BP (mmHg) | 9,711 | Low | 94 | Same population | CI NARROW, DOES NOT CROSS 0 | No | ⊕⊕⊕◯ Moderate |
| Diastolic BP (mmHg) | 9,465 | Low | 84 | Same population | CI NARROW, DOES NOT CROSS 0 | No | ⊕⊕⊕◯ Moderate |

*⊕⊕⊕⊕ High certainty; ⊕⊕⊕◯ Moderate certainty; ⊕⊕◯◯ Low certainty. Moderate certainty is colored as lighter yellow and Low certainty in light Pink. BP = blood pressure; CI = confidence interval; GRADE = Grading of Recommendations, Assessment, Development and Evaluation.*
