# Supplementary material for: Effectiveness of dietary, physical activity, and lifestyle interventions on weight and cardiometabolic outcomes in adults with overweight or obesity: an umbrella review of 112 systematic reviews and meta-analyses from 50 countries
Source: Obes Pillars. 2026 Sep 1;20:100324. doi: 10.1016/j.obpill.2026.100324 (PMC13571594; doi:10.1016/j.obpill.2026.100324)
Supplement: Supplementary file 5 [file mmc5.docx]

**Supplemental Table 4: Characteristics of included systematic reviews**

| **S.no** | **Authors & Year** | **Number of RCTs Included in the systematic review** | **No. of Participants** | **Intervention Duration** | **Intervention** | **Control** | **Country** | **Amstar grading** | **Study setting** |
| --- | --- | --- | --- | --- | --- | --- | --- | --- | --- |
| 1 | Ahmad Binmahfoz, 2025  <https://doi.org/10.1136/bmjsem-2024-002363> | 25 | 1608 | 9 weeks to 78 weeks | Dietary weight loss intervention PLUS resistance exercise. * Diet: Caloric restriction, meal replacements, and very low-calorie diets (VLCDs). * Resistance Exercise (RE): Free weights, machines, resistance bands, body weight, gym, lab or home-based, | Diet-only weight loss interventions. | USA | Low | Community |
| 2 | Ahmad Jayedi,   PhD, 2024  <https://doi.org/10.1001/jamanetworkopen.2024.52185> | 116 | 6680 | 8 weeks to 24 weeks | Supervised aerobic training program (e.g., walking, running). The duration of aerobic exercise per week ranged from 55 to 300 minutes | Control groups received no intervention, continued a sedentary lifestyle, or maintained their usual activity | North America, Asia, Europe, Australia, South America, and Africa | High | Community |
| 3 | Alline M Beleigoli, 2019  <https://doi.org/10.2196/jmir.9609> | 11 | 1525 | ≥ 26 weeks | Web-based digital health interventions. | Offline or in-person (face-to-face) interventions, or non-active interventions | USA | Critically low | community |
| 4 | Ana Marenco Kramer, 2023  <https://doi.org/10.1016/j.jesf.2023.09.002> | 13 | 335 | 5 weeks to 16 weeks | High-intensity interval training (HIIT). | Continuous aerobic training (CAT). | USA | Low | community |
| 5 | Angeliki Papadaki, 2020  <https://doi.org/10.3390/nu12113342> | 84 | 36,983 | 1.4 weeks to 365 weeks | Mediterranean-style diet | Usual care | Mediterranean regions; | High | Community |
| 6 | Anne Karen Jenum, 2019  <https://doi.org/10.1007/s00125-019-4905-2> | 6 | 1816 | 22–156 weeks | lifestyle modification program incorporating a healthy diet and/or physical activity | usual care | India, Norway, Netherlands, Scotland | High | Community |
| 7 | Arezoo Sadat Emrani, 2023  <https://doi.org/10.1186/s13643-023-02277-3> | 32 | 1117 | 3 weeks to 24 weeks | egg consumption | no egg consumption and bagel-based breakfast | USA. Australia, Thailand, UK, India, Denmark, Pakistan, Finland, and Mexico | High | Community |
| 8 | Armin Ebrahimzadeh, 2024  <https://doi.org/10.1016/j.ctim.2024.103025> | 21 | 1191 | 8 weeks to 12 weeks | Curcumin supplementation | Placebo | Iran | Low | Community |
| 9 | Bahar Kucuk, 2022  <https://doi.org/10.1017/jns.2022.90> | 8 | 456 | 8 weeks to 24 weeks | Alternate day fasting (ADF), which involves a 24-hour fast day where participants consume 0–25% of their daily energy needs, alternated with a 24-hour feast day with no caloric restriction or ad libitum intake | The control groups varied. In controlled studies, the comparator was either a continuous energy restriction (CER) diet or a group with no specified diet (ad libitum). | England, USA, China, | Critically low | Community |
| 10 | Bingjie Wang, 2025  <https://doi.org/10.1186/s12937-025-01178-6> | 15 | 758 | 6 weeks to 16 weeks | intermittent fasting (IF), including Intermittent Energy Restriction (IER), Alternate Day Fasting (ADF), the 5:2 diet, the 4:3 diet, and Time-Restricted Eating (TRE). | Control groups received no intervention (CON) or maintained their normal daily eating habits. | Denmark | Low | Community |
| 11 | Carlos Gomez-Garcia, 2025  <https://doi.org/10.3390/jfmk10030292> | 13 | 2100 | 13 weeks to 104 weeks | using mobile technology (mHealth), wearable activity tracker, mobile-based support or coaching, and training or awareness meetings about dietary habits, PA, or self-monitoring; | no intervention, usual care, waiting list control, or similar interventions without mHealth component; | Korea, Singapore, Czech Republic, USA, Mexico, China | Critically low | Community |
| 12 | Carmelo Pujia, 2025  <https://doi.org/10.2196/66887> | 11 | 1717 | 9 weeks to 52 weeks | Smartphone-only apps | Nutritional intervention, standard physical activity program, or no intervention (free diet) | UK, USA, Turkey | Low | Community |
| 13 | Carmen Varela, 2021  <https://doi.org/10.1186/s40337-021-00432-6> | 15 | 2426 | 12 weeks to 48 weeks | Web-based behavioral programs for overweight and obesity, delivered only by a website or a website combined with other technological devices. | Traditional behavioral treatment (e.g., face-to-face, group therapy, primary care) | Australia, USA | Low | Community |
| 14 | Cate Bulmer, 2025  <https://doi.org/10.1111/obr.13910> | 12 | 2805 | 52 weeks | The interventions were based on calorie restriction, with none restricting intake to less than 1100 kcal/day. | Standard care or exercise-only groups. | USA, UK, Italy, Finland, Spain | Critically low | community |
| 15 | Celeste E Naude, 2022  <https://doi.org/10.1002/14651858.CD013334.pub2> | 58 | 6925 | 13 weeks to 104 weeks | Low-carbohydrate weight-reducing diets | balanced-carbohydrate (45% to 65% of total energy) | China, USA, UK, Australia, Spain, Sweden, Norway, Germany, Japan, Canada, Israel, Singapore, New zealand | High | community |
| 16 | Chaitong Churuangsuk, 2021  <https://doi.org/10.1007/s00125-021-05577-2> | 21 | 1587 | 13 weeks to 156 weeks | dietary approaches like very low-energy diets (VLEDs), formula meal replacements, low-carbohydrate diets (LCDs), Mediterranean diets, | generally included other diet types like, low-carbohydrate diets were often compared against higher-carbohydrate/low-fat diets. | Barbados, India, Italy, Qatar, South Africa, Spain, Thailand, the UK and the USA. | Low | community |
| 17 | Chanthawat Patikorn, 2021  <https://doi.org/10.1001/jamanetworkopen.2021.39558> | 11 | 6883 | 9 weeks to 13 weeks | Intermittent Fasting (IF), categorized into four types: Zero-calorie Alternate-Day Fasting (ADF): Alternating days of zero caloric intake and days of ad libitum eating. * Modified Alternate-Day Fasting (MADF): Alternating days of ad libitum eating and days of fasting 0 to 600 kcal per day, for 3 to 5 days per week. * 5:2 Diet: Fasting for 1 to 2 days per week (TRE): Fasting for 12 to 24 hours per day. | Regular diet | Australia | High | Community |
| 18 | Chenhan Ma, 2017  <https://doi.org/10.1136/bmj.j4849> | 54 | 30206 | 52 weeks | low-fat, weight-reducing diets | The interventions were compared against a control intervention. | USA, Australia, Brazil, UK, Netherlands, Poland, Finland, China, Canada, Japan, | Low | community |
| 19 | Chenlin Gao, 2019  <https://doi.org/10.1186/s12944-019-1127-z> | 9 | 329 | 4 weeks to 52 weeks. | Dietary supplementation with Resistant Starch (RS). The dose of RS varied across studies. | Control groups were supplemented with a type of digestible carbohydrate that is not fermentable (non-RS). | China | Low | community |
| 20 | Chenxi Hu, 2024  <https://doi.org/10.1038/s41598-024-59386-9> | 15 | 756 | 8 weeks to 56 weeks | The mode of intervention in the study had to be resistance circuit training. (RCT) | The control group’s intervention was no exercise. | China | Low | Community |
| 21 | Claire D Madigan, 2022  <https://doi.org/10.1136/bmj-2021-069719> | 34 | 8000 | 52 weeks to 104 weeks | Behavioral weight management interventions delivered in primary care, focused on weight loss | no treatment or minimal intervention or attention control | USA, UK, Spain | Low | primary care |
| 22 | D. Power, 2025  <https://doi.org/10.1111/jhn.13410> | 47 | 6729 | 99 weeks | Psychological interventions that had an Emotional Eating (EE) component. Intervention types included standard behavioral interventions, | usual care | Netherlands, Finland, Italy, Portugal, Belgium, Spain, Switzerland, Brazil, Turkey, Russia, UK, Taiwan | Moderate | Community |
| 23 | Dr Deirdre K Tobias ScD, 2015  <https://doi.org/10.1016/S2213-8587(15)00367-8> | 53 | 68,128 | 52 weeks | Low-fat diets ranging from ≤10% to ≤30% of calories from fat. | Other higher-fat dietary interventions, low-carbohydrate diets, other moderate-to-high-fat diets, or usual diet. | USA, Australia, Canada, Germany, Iran, Israel, New Zealand, Spain, Sweden, and the UK. | Moderate | Community |
| 24 | Duc Tran Quang, 2025  <https://doi.org/10.1371/journal.pone.0314685> | 36 | 914 | 2 weeks to 48 weeks | Time-restricted eating (TRE), delivered through various formats (e.g., 12:12, 14:10, 16:8). Interventions combining TRE with other dietary or exercise approaches were also included. | The meta-analysis utilized pre- and post-intervention analyses, | Australia, Brazil, Canada, China, Germany, Iran, Italy, Kenya, South Korea, the Netherlands, Switzerland, Taiwan, Turkey, the United Kingdom, and the United States. | Low | Community |
| 25 | Ehrika Vanessa Almeida de Menezes, 2019  <https://doi.org/10.1186/s12937-019-0457-z> | 24 | 868 | 2 weeks to 104 weeks | Paleolithic Diet. This diet is generally based on fish, lean meats, eggs, vegetables, fruits, and nuts, and excludes cereals, dairy products, and sugar | Other control diets based on recommendations | Sweden | Critically low | Community |
| 26 | Evangelia Malakou, 2018  <https://doi.org/10.3390/nu10111577> | 11 | 1684 | 12 weeks to 313 weeks | combination of the Mediterranean Diet (MD) and Physical Activity (PA). | No treatment, MD alone, PA alone, or a different dietary and/or PA treatment (e.g., low-fat diet and/or different volume/intensity of PA). | Italy, Spain, Greece, Croatia, Germany, Australia | Low | clinical/ health setting |
| 27 | Felipe da Fonseca Silva Couto, 2025  <https://doi.org/10.3390/ijerph22071152> | 261 | 62,407 | 13 weeks to 104 weeks | eHealth interventions, specifically mobile applications (mHealth) and web-based programs, designed for weight loss. | Conventional treatment for overweight or obesity, including diet, exercise, behavioral therapy, pharmacological interventions, or minimal/no intervention, excluding digital technologies. | USA, Europe, Oceania, Asia | Moderate | Community |
| 28 | Gabrielle Maston, 2019  <https://doi.org/10.3390/bs9120144> | 11 | 865 | 4 weeks to 142 weeks | Severely energy-restricted diets with a daily energy intake ≤5000 kJ (≤1200 kcal) for ≥4 weeks. | The comparison arms were classified based on diet duration: 4 weeks' duration and ≥6 weeks' duration. | USA | Critically low | Community |
| 29 | Gina M Wren, 2024  <https://doi.org/10.1002/oby.23976> | 9 | 796 | 8 weeks to 52 weeks | Planned-pause interventions, which involved periods of energy restriction interrupted by planned intervals of no less than one week. | Continuous energy restriction (CER), where participants maintained a continuous period of active weight loss effort without planned breaks. | Australia, USA, Canada, Turkey | Critically low | Community |
| 30 | Giovanna Muscogiuri, 2021  <https://doi.org/10.1159/000515381> | 15 | 835 | 52 weeks | A very low-calorie ketogenic diet (VLCKD). low carbohydrate content (<50 g/day), a specific amount of protein and fat, and a daily intake of about 500-800 calories. | a standard low-calorie diet (LCD). | Spain, Italy | Critically low | community |
| 31 | Grainne O'Donoghue, 2020  <https://doi.org/10.1111/obr.13137> | 45 | 3566 | 8 weeks to 52 weeks | categories of exercise interventions Aerobic exercise; vigorous intensity (AE-V)  Aerobic exercise; moderate intensity (AE-M) Resistance training; high load (R-HI)  Resistance training; low-to-moderate load (R-LM).  Combined high; vigorous intensity aerobic/high-load resistance  (COM-HI)  Combined low-moderate; moderate intensity aerobic/low-to moderate load resistance (COM-LM) | no exercise | USA, UK, Australia, South Africa | Low | community |
| 32 | Haifeng Zhu, 2023  <https://doi.org/10.1016/j.ctcp.2023.101766> | 20 | 565 | 2 weeks to 24 weeks. | Water-based exercise (WBE), including aerobic exercise (AE), resistance exercise (RE), combined exercise (CE), or aquatic high-intensity interval training (HIIT) | No-exercise control | Turkey, Iran, USA, China | Moderate | Community |
| 33 | Hamidreza Raeisi-Dehkordi, 2019  <https://doi.org/10.1093/advances/nmy108> | 25 | 1280 | 3 weeks to 28 weeks. | Oral ingestion of pure or conventional canola oil (CO). The amount of canola oil used was ≥10 g/d. | The control groups consumed other sources of dietary fats such as olive oil, sunflower oil, saturated fat, fish oil, and nuts. | Iran, Germany, India, Sweden, Finland, Australia, USA, Canada | High | community |
| 34 | Hamish A. Fernando, 2019  <https://doi.org/10.3390/nu11020478> | 70 | 2947 | 4 weeks | Ramadan fasting (abstinence from eating and drinking from sunrise to sunset) | Measurements taken before Ramadan (pre-R) and 2-5 weeks after the end of Ramadan (follow-up) were used for comparison. | Middle East, North Africa; South Asia; South East Asia; | Critically low | Community |
| 35 | Han Shi Jocelyn Chew, 2022  <https://doi.org/10.2196/40141> | 16 | 2870 | 12 weeks to 24 months. | smartphone apps for weight loss/management. | the control condition provided app-based food logging. | USA | Low | Community |
| 36 | Han-Na Kim, 2019  <https://doi.org/10.3390/ijerph17010015> | 5 | 1830 | 9 weeks to 26 weeks | Smartphone-based health programs using applications (apps) | hand book, text messages or printed materials | USA | Critically low | university setting and local community setting |
| 37 | Hao Qiu, 2025  <https://doi.org/10.3389/fnut.2025.1537291> | 8 | 642 | 8 weeks to 24 weeks. | Multicomponent Training (MCT) (integrates ≥3 types of training, e.g., strength, aerobic, balance, flexibility); | Educational or psychological intervention or no intervention | China | Moderate | community |
| 38 | Harris Leanne; 2018  <https://doi.org/10.11124/jbisrir-2016-003248> | 6 | 223 | 13 weeks to 52 weeks | Intermittent energy restriction, defined as consuming ≤800 kcal on one to six days per week. The specific regimens varied and included alternate-day fasting and fasting for two to four days per week. | The interventions were compared against two types of control groups: no treatment (ad libitum diet) or usual care (continuous energy restriction ∼25% of recommended energy intake) | USA, UK | Low | Community |
| 39 | Hossein Bahari, 2023  <https://doi.org/10.1002/fsn3.3739> | 22 | 1124 | 2 weeks to 24 weeks. | Pomegranate consumption (Juice, Extract, or Seed oil). | placebo, water, no juice, routine recommendations | Iran, USA, UK, Mexico, South Korea, Scotland, Serbia, Greece, and Bosnia and Herzegovina. | Moderate | Community |
| 40 | Iolanda Cioffi, 2018  <https://doi.org/10.1186/s12967-018-1748-4> | 11 | 630 | 8 weeks to 24 weeks. | The intervention was intermittent energy restriction (IER), which involved regimens with weekly "fast days" (providing < 25% of daily energy needs) and "feed days" (with either ad libitum or balanced energy consumption). | The control group was continuous energy restriction (CER), which involved a daily energy restriction of approximately 25% of daily energy requirements. | USA, UK, Australia, Norway | Low | Community |
| 41 | Iván Cavero-Redondo, 2020  <https://doi.org/10.3390/nu12071977> | 20 | 2196 | 6.5 weeks to 104 weeks | lifestyle (diet and physical activity) mHealth self-monitoring. The mHealth devices used were Personal Digital Assistants (PDAs), smartphones, or web-based approaches | usual care, paper record, and wait-lists | USA, UK, South Korea, New zealand, Finland, Australia | Low | Community |
| 42 | J Eales, 2015  <https://doi.org/10.1038/ijo.2015.202> | 3 | 139 | 2 weeks to 24 weeks | consumption of yoghurt (all flavors and fat contents were eligible) containing the standard symbiotic cultures. | compare yoghurt with at least one of the following: low or no yoghurt consumption; placebo (e.g. yoghurt-like products not including live bacteria); non-yoghurt substances (e.g. milk); non-yoghurt interventions (e.g. nutritional counselling). | USA, Korea, Malaysia, Spain, Australia, Canada, France, | High | Community |
| 43 | J. Salse-Batán, 2022  <http://dx.doi.org/10.1111/jir.12928> | 17 | 636 | 6 weeks to 52 weeks. | Physical exercise training programmes. The interventions included cardiovascular training, combined training (e.g., strength and endurance), whole-body vibration training, and balance training. | Control groups were not assigned to any exercise programmes during the intervention. They were either required to avoid exercise, continue with daily life activities, or perform social activities. | USA | Critically low | community |
| 44 | Jakub Morze, 2021  <https://doi.org/10.1111/obr.13218> | 32 | 4774 | 24 weeks | Different long-term training modalities: • Aerobic Training (AET), Resistance Training (RT), Combined Training (CT), High-Intensity Interval Training (HIIT), | Control (CONT) or Minimal Intervention (MI). | USA, Canada, Tunisia, Korea, China, Denmark, and Israel | High | Community |
| 45 | Jamie Hartmann-Boyce, 2022  <https://doi.org/10.1002/oby.23505> | 169 | 34228 | 52 weeks | Behavioral Weight Management Programs (BWMPs), which aim to achieve weight loss through diet, physical activity, or both. | minimal-intervention control treatments. | USA, Europe, Africa | Low | community, health care, home |
| 46 | Jana Jabbour, 2022  <https://doi.org/10.3389/fnut.2022.821096> | 36 | 4916 | ≥ 52 weeks | Dietary Interventions: Moderate Macronutrients (MMs), Low Fat/High Carbohydrate (LFHC), and High Fat/Low Carbohydrate (HFLC) | Usual Diet (UD) | USA, Europe, Australia | Low | Community |
| 47 | Jie Men, 2025  <https://doi.org/10.3389/fendo.2025.1526991> | 87 | 4213 | 2 weeks to 48 weeks, | High-intensity interval training (HIIT). | no exercise or low-intensity exercise control | Norway, Switzerland, Belgium, Denmark, Spain, UK, Sweden, Germany, Italy, France, Canada, the United States, Brazil, China, Iran, Japan, South Korea, Thailand, Indonesia, Australia, Egypt, | Low | community |
| 48 | Jinming Huang, 2024  <https://doi.org/10.1186/s12966-024-01657-9> | 47 | 3363 | 52 weeks | Four caloric restriction regimens were evaluated: Alternate Day Fasting (ADF), Short-Term Fasting (STF), Time-Restricted Eating (TRE), and Continuous Energy Restriction (CER). | The control group was a general diet without time or calorie restrictions. | China, Iran, Brazil, Poland, USA, Switzerland, Ireland, Germany, Australia, South Korea, Norway | Low | Community |
| 49 | Joana M. Correia, 2021  <https://doi.org/10.3389/fnut.2020.625240> | 66 | 332 | 1.4 weeks to 12 weeks. | Intermittent Fasting (IF), including any type: Ramadan IF, Time-Restricted Feeding (TRF), Alternate-Day Fasting (ADF), or Intermittent Energy Restriction (IER). | "Normal diet" to Continuous Energy Restriction (CER)). | USA | Low |  |
| 50 | Jonathan P. Bonnet, 2020  <https://doi.org/10.1002/oby.22791> | 7 | 425 | 4 weeks to 16 weeks. | Breakfast skipping. generally involved extending the overnight fast until between 10:00 h and 12:00 h the following morning. | Breakfast consumption. This typically involved a predefined breakfast meal routine. | USA, UK | High | Community |
| 51 | Jonathan Sackner-Bernstein, 2015  <https://doi.org/10.1371/journal.pone.0139817> | 17 | 1797 | 8 weeks to 104 weeks | A low-carbohydrate (LoCHO) diet was defined as a total carbohydrate intake of 120 g per day or less. | A low-fat (LoFAT) diet was defined as having less than 30% of daily energy from fat | USA | Critically low | community |
| 52 | Jong Han Choi, 2022  <https://doi.org/10.4093/dmj.2022.0038> | 24 | 3660 | 8 weeks to 24 weeks. | Moderately-low carbohydrate diet (mLCD): Carbohydrates are 26% to 45% of total caloric intake. Low carbohydrate diet (LCD): Carbohydrates are 10% to 25% of total caloric intake. Very-low carbohydrate diet (VLCD): Carbohydrates are less than 10% of total caloric intake. | Isocaloric control diets that were equivalent in total calories to the intervention diets. | United States, United Kingdom, Australia, Europe, Bahrain, Israel, China, Japan, and Taiwan | Critically low | Community |
| 53 | Ke Yao, 2024  <https://doi.org/10.1016/j.jnha.2024.100165> | 9 | 285 | 6 weeks to 12 weeks. | Various types of intermittent fasting (IF) interventions, including alternate-day fasting (ADF), 5:2 fasting (2DW 2 days a week), time-restricted eating (TRF), and Ramadan fasting (FCR). | A regular diet (RD), which involved no change in diet and no dietary intervention | Poland, Malaysia, USA, China, Germany | Critically low | Community |
| 54 | Kevin C Maki, 2019  <https://doi.org/10.3390/nu11061245> | 12 | 973 | 12 weeks to 16 weeks. | The intervention was a higher intake of whole-grain (WG) foods, such as oats, quinoa, brown rice, or foods made with WGs like whole-grain bread or cereal. The WG intake in the RCTs ranged from 32 g/day to 215 g/day | The control groups in the RCTs consumed a diet with lower or no whole-grain intake, which sometimes included refined-grain products. | USA, Spain, Sweden, Iran, | Critically low | Community |
| 55 | Ke-Wen Wan, 2024  <https://doi.org/10.1136/bmjsem-2023-001831> | 15 | 361 | 2 weeks to 8 weeks | Time-restricted feeding (TRF), a form of intermittent fasting, which involves limiting daily caloric intake to a consistent 6–12-hour window and fasting for the remaining hours | Normal diet (ND) | USA, Portugal, Italy, Canada | High | Community |
| 56 | Kuo-Jen Hsu, 2019  <https://doi.org/10.3390/nu11092163> | 15 | 748 | 8 weeks to 24 weeks. | The study investigated the effects of three types of interventions: Exercise (aerobic, resistance, and combined). Nutritional intervention (including low-calorie high-protein (LCHP) diets and protein supplementation). A combination of exercise and nutritional intervention. | Control groups in the included studies received no intervention | Canada, Usa, Tawain, Japan, Germany, Italy, Brazil, Korea | Critically low | Community |
| 57 | Kyoung-Bae Kim, 2019  <https://doi.org/10.7570/jomes.2019.28.4.278> | 35 | 906 | 2 weeks to 48 weeks, | Exercise (various modes: aerobic, resistance, combined, yoga, Pilates) | Routine care, diet therapy, no treatment, placebo, | South Korea | Critically low | community |
| 58 | L. Harris, 2017  <https://doi.org/10.1111/obr.12593> | 5 | 376 | 14 weeks to 48 weeks | Weekly Intermittent Energy Restriction (IER). This was defined as consuming a reduction in energy intake, intermittently for a period of ≥7 days, interspersed with periods of no prescribed energy restriction or a lesser degree of prescribed energy restriction. | Usual care | USA, Sweden, Canada | Low | face to face through weekly group sessions |
| 59 | Laura Chiavaroli, 2018  <https://doi.org/10.1136/bmjopen-2017-019438> | 32 | 2448 | 3 weeks to 12 weeks | The intervention involved consuming pasta in the context of low-glycaemia index (low-GI) dietary patterns. | The control group consumed higher-GI dietary patterns. | Sweden | High | community |
| 60 | Lauren Ball, 2021  <https://doi.org/10.1136/bmjnph-2020-000195> | 4 | 476 | 4 weeks to 13 weeks | exclusive provision of foods, ingredients, oral nutrition support, or food vouchers to participants in primary care settings, with the aim of weight loss. | Baseline, usual care, no care, or minimal care | USA, UK, Israel | Low | primary care |
| 61 | Lee Hooper, 2020  <https://doi.org/10.1002/14651858.cd013636> | 37 | 57,079 | 26 weeks | lower-fat diet, defined as aiming for a fat intake of ≤30% of total energy (≤30%E). | The control group consumed a higher-fat diet, defined as one that aimed to include >30% of total energy from fat. | North America, Europe, Australia, China | High | Community |
| 62 | Leila Jahangiry, 2021  <https://doi.org/10.1186/s41043-021-00240-3> | 8 | 779 | 12 weeks to 24 weeks | Web-based interventional programs/web-user intervention | Non-web user control arm. | USA, Germany, Australia, South Korea | Critically low | Community |
| 63 | Leona Yuen-Ling Leung, 2025  <https://doi.org/10.1016/j.clnu.2025.01.017> | 23 | 2821 | 2 weeks to 156 weeks | The intervention was a ketogenic diet (KD) or a low-carbohydrate diet (LCD), defined as a diet with a maximum carbohydrate intake of ≤100 g/d. | The control group followed a usual diet or dietary intake without restriction, such as a low-fat diet. | United States; Australi,Spain ,Brazil, China, and Norway, Korea , Jordan, Sweden, Italy, and Malaysia | Low | Community |
| 64 | Liana L Guarneiri, 2021  <https://doi.org/10.1093/advances/nmaa113> | 55 | 3976 | 3 weeks to 156 weeks | various nut diets ( mixed nut, walnut, almonds, Pistachio) | Habitual diet | New zealand, China, UK, Australia, USA, Spain, Iran, India, Turkey, Korea | Low | Community |
| 65 | Lifu Lei, 2022  <https://doi.org/10.3389/fnut.2022.935234> | 33 | 3939 | 26 weeks to 104 weeks | Low-Carbohydrate Diets (LCD). Defined as carbohydrate intake ≤40% of total energy intake | Low-Fat Diets (LFD). Defined as fat intake <30% of total energy intake. | USA, Australia, Spain, Germany, Israel, UK, Canada, New Zealand, Norway, Sweden, and Japan | Critically low | Community |
| 66 | Long Ge, 2020  <https://doi.org/10.1136/bmj.m696> | 121 | 21942 | 26 weeks | 14 popular named diets (e.g., Atkins, Zone, DASH, Mediterranean, Ornish) or alternative diets, categorized into three macronutrient patterns: low carbohydrate, low fat, and moderate macronutrient. | usual diet (reference diet) | Canada, USA, Switzerland, China, and Iran | Critically low | Community |
| 67 | Maite M. Schroor, 2023  <https://doi.org/10.1016/j.advnut.2023.10.003> | 28 | 2043 | 2 weeks to 52 weeks. | Three different types of Intermittent Energy Restriction (IER) diets were studied: Time-restricted eating (TRE): Eating is allowed only within a specific daily window, typically less than 12 hours. Alternate-day fasting (ADF): Individuals alternate between days of energy restriction and days of consuming food as desired 5:2 diet: Individuals have two energy-restricted days per week and five days of normal eating. | The control group followed a Continuous Energy Restriction (CER) diet. | Taiwan, the Netherlands, Australia, the UK, and Brazil | Moderate | Community |
| 68 | Manuela Chiavarini, 2025  <https://doi.org/10.3390/nu17132200> | 22 | 3565 | ≤ 26 weeks | Digital health interventions (e-Health and m-Health), either standalone or as part of mixed approaches. | No treatment or traditional management approaches | USA, Canada, Portugal, Spain, England, Belgium, Germany, Austria, India, South Korea, Korea, and Australia | Low | Community |
| 69 | Maria-Inti Metzendorf, 2024  <https://doi.org/10.1002/14651858.CD013591.pub2> | 18 | 2703 | 8 weeks to 104 weeks | Integrated smartphone applications using at least two behavior change techniques, designed to support healthy behaviors. The interventions targeted physical activity, cardiorespiratory fitness, weight loss, diet, or self-efficacy. | The interventions were compared against several types of comparators: No or minimal intervention (NMI) A different smartphone app. Personal coaching. Usual care. | Spain, USA, UK, Spain, Belgium, Switzerland, Japan, Germany, New zealand, | High | Community |
| 70 | Marlene A. van Baak, 2019  <https://doi.org/10.3390/nu11081916> | 21 | 2875 | 13 weeks to 52 weeks | The review examined dietary strategies for weight loss maintenance, focusing on Increased protein intake. | different ad libitum diets | Netherlands, Denmark, Poland, Australia, Germany, France, UK, USA | Moderate | community |
| 71 | Matthew Snelson, 2019  <https://doi.org/10.3390/nu11081833> | 20 | 670 | 1 weeks to 12 weeks. | Dietary supplementation with Resistant Starch Type 2 (RS2) | A placebo | USA | High | Community |
| 72 | Michelle Headland, 2016  <https://doi.org/10.3390/nu8060354> | 9 | 981 | interventions with a minimum 6 months duration. | intermittent energy restriction (IER), which involved various strategies such as alternate-day fasting (ADF) and the 5:2 regimen. | The control group followed a continuous energy restriction (CER), a traditional form of daily dietary restriction | USA | Critically low | Community |
| 73 | Min Zhuang, 2022  <https://doi.org/10.3389/fphys.2022.917525> | 12 | 614 | < 24 weeks | Aerobic Training (AT), Resistance Training (RT), and Aerobic combined with Resistance Training (MT). | No-PA control group | China, USA | Critically low | Community |
| 74 | Mohamad Motevalli, 2025  <https://doi.org/10.2196/73656> | 30 | 3960 | 8 weeks to 104 weeks | Guideline-Based Digital Physical Activity (PA) and Exercise Interventions. | usual care | United States, Australia, Korea, United Kingdom, Germany, Spain, Sweden, France, Canada, Belgium, Singapore, China, and Hong Kong. | Critically low | Community |
| 75 | Mohammadreza Moradi Baniasadi, 2025  <https://doi.org/10.1038/s41387-025-00386-7> | 20 | 1387 | 8 weeks to 36 weeks. | Turmeric and/or Curcumin supplementation. | Placebo ( e.g. Starch , Lactose , Corn Starch Flour , Polysorbate , Cooked Rice Flour) | Iran, Thailand, Mexico, Japan, Italy, Australia, and Brazil | High | Community |
| 76 | Mona Kholdebarin, 2024  <https://doi.org/10.1002/fsn3.4596> | 19 | 1601 | 4 weeks to 52 weeks | Chitosan supplementation | Placebo | Japan, United Kingdom, Singapore, Finland, Canada, New Zealand, USA, Taiwan, Mexico, Germany, India, Spain, Poland, and South Korea. | High | Community |
| 77 | Mousa Khalafi, 2025  <https://doi.org/10.3390/healthcare13070776> | 53 | 2873 | 6 weeks to 52 weeks | Concurrent Training (CT): A combination of Aerobic Training (AT) and Resistance Training (RT) in the same or separate sessions | Aerobic Training (AT) alone and/or Resistance Training (RT) alone. | Iran | Low | Community |
| 78 | Néma D. McGlynn, 2022  <https://doi.org/10.1001/jamanetworkopen.2022.2092> | 17 | 1733 | 3 weeks to 52 weeks | Low- and no-calorie sweetened beverages (LNCSBs) as a substitute for sugar-sweetened beverages (SSBs) or water. | Sugar-sweetened beverages (SSBs) or water. | USA, UK | High | Community |
| 79 | Nerys M. Astbury, 2019  <https://doi.org/10.1111/obr.12816> | 23 | 7,884 | 52 weeks to 209 weeks | Meal replacements (MR) incorporating a hypo energetic diet (daily energy intake restricted to≥3347 kJ (800 kcal)/day). Interventions were categorized by level of support: MR diet, MR diet + support, and MR diet + enhanced support. | Interventions that did not include MR for weight loss, categorized as: diet only, diet + support, or minimal intervention (e.g., printed leaflet or one-off lecture). | Australia, China, Malaysia, Singapore, Thailand, Germany, USA | High | Community |
| 80 | Nia Coupe, 2019  <https://doi.org/10.1186/s12889-019-7185-3> | 10 | 1320 | 4 weeks to 52 weeks | Commitment devices used alongside behavioral lifestyle interventions targeting diet and/or physical activity. The commitment devices were "soft" (non-financial), such as behavioral contracts, pledges, and public or verbal agreements. | The control groups varied and included no intervention, or similar interventions without the commitment device. Some studies compared different variations of commitment devices. | Denmark, USA, Japan, India | Critically low | Community |
| 81 | Omid Sadeghi, 2020  <https://doi.org/10.1093/advances/nmz076> | 21 | 1798 | 2 weeks to 24 weeks. | The intervention involved the consumption of whole-grain products or diets rich in whole-grain foods. | The control groups consumed a diet low in or without whole grains, such as refined-grain products or diets. | USA, Europe, Australia | Low | Community |
| 82 | Paul Blazey, 2023  <https://doi.org/10.1186/s12966-023-01532-z> | 17 | 516 | 2 weeks to 52 weeks | Restricted-frequency eating (≤ three meals per day). | Unrestricted-eating frequency (≥ four meals per day). | USA, Europe, Iran, Brazil | High | Community |
| 83 | Qiao-Yi Chen, 2024  <https://doi.org/10.3390/nu16070963> | 8 | 1060 | 13 weeks to 26 weeks | Water intake intervention. This involved: (1) Addition: promoting water intake (e.g., increasing water consumption) or  (2) Substitution: recommending replacing other beverages with water | Any other beverages or placebo | USA, Europe, Iran, Mexico | Low | Community |
| 84 | Ran Li, 2025  <https://doi.org/10.2196/59507> | 6 | 1590 | 10 weeks to 209 weeks | Wearable activity tracker-based interventions | usual care | Australia, England, United States, Japan, Germany, and Finland | Low | community |
| 85 | Rubén Fernández-Rodríguez, 2021  <https://doi.org/10.3390/nu13072251> | 105 | 6768 | 12 weeks to 18 weeks | nut consumption (i.e., almonds, cashews, hazelnuts, peanuts, pistachios, walnuts, and mixed nuts, | control groups with habitual diet, placebo or isocaloric snacks, low-fat diets or standard diets according to dietary guidelines, free of nut consumption; | Iran, USA, Spain, Australia, Brazil, Turkey, Taiwan, France, New zealand, Sweden, Pakistan, India, | High | Community |
| 86 | Shreya Chawla, 2020  <https://doi.org/10.3390/nu12123774> | 38 | 674 | 26 weeks to 52 weeks | Low-Carbohydrate Diet: Defined as having less than 40% carbohydrate content. Low-Fat Diet: Defined as having less than 30% total fat content. | The low-fat diet group was the control for the low-carbohydrate diet group, and vice versa. | Spain, USA, UK, Australia, New Zealand, Canada | Low | community |
| 87 | Siew Min Ang, 2021  <https://doi.org/10.2196/28185> | 21 | 20436 | 8 weeks to 52 weeks | Interventions that incorporate a mobile app. multicomponent intervention (apps used with other components, e.g., face-to-face consultation, phone calls). | usual care alone | China, Hong Kong, India, Japan, Singapore, South Korea, Taiwan, | Low | Community |
| 88 | Silvia Sanca-Valeriano, 2023  <https://doi.org/10.1016/j.heliyon.2023.e20402> | 22 | 532 | 4 weeks to 16 weeks.  . | High-Intensity Interval Training (HIIT). | Moderate-Intensity Continuous Training (MICT) | Italy | Low | Community |
| 89 | Simone Perna, 2020  <https://doi.org/10.3390/nu12082365> | 4 | 399 | 12 weeks to 52 weeks | Polyglucosamine supplementation, a chitosan derivative, in tablet or capsule form. This was used in conjunction with lifestyle behavioral therapies, such as caloric restriction and physical activity. | A placebo group that also received the same lifestyle coaching and behavioral interventions, including caloric restriction and physical activity. | Italy and other European nations | Low | community |
| 90 | Stephen D. Anton, 2017  <https://doi.org/10.3390/nu9080822> | 16 | 1091 | 17 weeks | 7 popular diets Atkins, DASH, Glycemic-Index, Mediterranean, Ornish, Paleolithic, and Zone | usual care | USA | Critically low | community |
| 91 | Taibah Almesbehi, 2022  <https://doi.org/10.1016/j.appet.2022.106424> | 9 | 707 | >4 weeks | Various exercise training. These included endurance training (such as walking, running, and cycling), resistance training, and combined training. | The control groups typically maintained their usual sedentary routine and were offered healthy lifestyle advice after the study. | Australia, USA, Denmark, Iran, South Korea | High | Community |
| 92 | Tainah O. P. Arisi, 2024  <https://doi.org/10.3390/nu16121919> | 31 | 1986 | 4 weeks to 24 weeks | intervention: cocoa-extract supplement or ≥70% cocoa dark chocolate for ≥4 weeks; | comparison: placebo or <70% cocoa white/milk chocolate; | USA | Low | community |
| 93 | Tanja Črešnovar, 2023  <https://doi.org/10.3390/nu15234911> | 9 | 526 | 8 weeks to 14 weeks. | Time-Restricted Eating (TRE) combined with caloric restriction (CR). The TRE regimens involved daily fasting periods of 14-18 hours and eating windows of 6-10 hours | A control group with caloric restriction (CR) alone. | Brazil, USA, UK, Taiwan, China, Turkey | Low | Community |
| 94 | Thea Toft Hansen, 2021  <https://doi.org/10.3390/nu13093193> | 43 | 4802 | 8 weeks to 104 weeks. | intervention involved dietary interventions with increased protein intake, ranging from 18% to 59% of total energy | Control groups received either a standard diet with a lower protein content (ranging from 2% to 20% | USA | Moderate | community |
| 95 | Theogene Habumugisha, 2023  <https://doi.org/10.1093/nutrit/nuad055> | 14 | 1475 | 4 weeks to 24 weeks | Meat- and/or dairy-reduced diet | Habitual (standard) diet rich in meat and/or dairy | Canada, USA, Australia, Germany, South Korea | Low | Community |
| 96 | Thomas Semlitsch, 2021  <https://doi.org/10.1002/14651858.CD008274.pub4> | 8 | 2100 | 26 weeks to 156 weeks | Weight-reducing diets (e.g., dietary counselling, caloric restrictions, reduction in fat intake) | No dietary intervention | USA, UK, Finland, Germany, Italy | High | Community |
| 97 | Thompson Sharon V, 2017  <https://doi.org/10.3945/ajcn.117.163246> | 12 | 609 | 2 weeks to 17 weeks. | Isolated soluble fiber supplementation. Supplements varied in type and dose (3 to 34 g/d) | Placebo treatments without soluble fiber. | USA | Low | community |
| 98 | Víctor Siles-Guerrero,2024  <https://doi.org/10.3390/nu16203533> | 10 | 623 | 4 weeks to 48 weeks | Fasting-based strategies (FBS), which include various forms such as time-restricted eating (TRE), intermittent fasting (IF), and alternate-day fasting. | Continuous caloric restriction (CCR), which involves a consistent daily reduction in calorie intake. | Italy | Critically low | Community |
| 99 | Weihao Wang, 2020  <https://doi.org/10.1186/s12872-020-01337-z> | 22 | 1865 | >2weeks | The intervention was the consumption of a whole grain food diet. The specific whole grains included barley, oat, wheat, rye, and quinoa. | The control groups received a diet that did not include whole grains (referred to as a non-whole grain or refined grain diet). | South Korea | Low | Community |
| 100 | Welma Stonehouse, 2016  <https://doi.org/10.3390/nu8070394> | 27 | 1278 | 8 weeks to 24 weeks | Increased consumption of dairy products (whole food or supplements) as part of an energy-restricted diet. | A low-dairy control diet. | USA, CANADA, Australia, Mexico, UK, Iran, Japan, Brazil | Low | Community |
| 101 | Yeo Jin Choi, 2020  <https://doi.org/10.3390/nu12072005> | 14 | 734 | 1 week to 104 weeks. | The intervention was a ketogenic diet. | control groups received various low-fat diets, which included low-calorie, low-glycemic index, or high-carbohydrate low-fat diets. | USA, Spain, Norway, Canada, Greece, China, Australia | Critically low | community |
| 102 | Yi Wang, 2021  <https://doi.org/10.3389/fphys.2021.643455> | 11 | 393 | 8 weeks to 24 weeks. | Pilates exercise. | Other physical exercises or without any intervention (normal lifestyle) | Korea, Turkey, Iran, the USA, India, and China. | Low | community |
| 103 | Yixun Xie, 2024  <https://doi.org/10.3390/nu16193390> | 20 | 1241 | 4 weeks to 12 weeks. | The study examined time-restricted eating (TRE) as a dietary intervention. | The interventions were compared to a non-intervention, or ad libitum diet, which served as a control. | USA, Norway, Brazil, Iran, Poland, China, Germany | Moderate | Community |
| 104 | Yongchao Xie, 2024  <https://doi.org/10.3390/nu16173007> | 78 | 5219 | 4 weeks to 52 weeks | Combinations of different exercise (aerobic, resistance training, and mixed exercise) and dietary interventions (Calorie Restriction (CR), 5/2 Intermittent Fasting (5/2F), Time-Restricted Feeding (TRF), and Ketogenic Diet (KD)) | no intervention, exercise only (EX), or dietary intervention only (CR, 5/2F, TRF, or KD). | USA | Low | community |
| 105 | Yue Lun Loh, 2023  <https://doi.org/10.1038/s41366-023-01304-6> | 28 | 13195 | 8 weeks to 104 weeks | Social media-based interventions | usual care | Australia, USA, UK, Malaysia | Moderate | Community |
| 106 | Zhicheng Guo, 2022  <https://doi.org/10.3390/ijerph19084638> | 9 | 124 | < 4 weeks | participants who received HIIT + fasting were included in this study. HIIT intervention measures were used: the exercise intensity at 80 to 100% HRmax/peak oxygen consumption (VO2peak) for 30 s to 4 min, passive recovery, or low-intensity aerobic exercise in the intermittent period for a maximum of 4 min. | control group included (HIIT alone, fasting alone, or normal intervention | USA, Canada, Denmark, China | Low | community |
| 107 | Zhila Semnani-Azad, 2025  <https://doi.org/10.1136/bmj-2024-082007> | 99 | 6582 | 3 weeks to 52 weeks | Intermittent Fasting (IF) Diets: Alternate Day Fasting (ADF), Time Restricted Eating (TRE), Whole Day Fasting (WDF), | unrestricted energy intake | Australia, Austria, Brazil, Canada, China, Czech Republic, Germany, Indonesia, Iran, Italy, Malaysia, Mexico, Netherlands, Norway, Poland, Portugal, South Korea, Spain, Sweden, Taiwan, Thailand, Turkey, UK, USA | High | Community |
| 108 | Zhongxu Hu, 2025  <https://doi.org/10.3389/fphys.2025.1609013> | 8 | 410 | 8 weeks to 52 weeks | Circuit-Based Resistance Training (CBRT) | No exercise intervention | China | Critically low | community |
| 109 | Zi-Han Dai,2025  <https://doi.org/10.1007/s13679-025-00645-9> | 12 | 616 | 4 weeks to 16 weeks. | intermittent fasting (IF) and exercise (IF+EX). The types of IF included time-restricted eating (TRE), alternate-day fasting (ADF), 5:2 diet, and Ramadan IF | The control group received intermittent fasting (IF) alone. | USA, Tunisia, Brazil, Korea, Thailand, Norway, Australia | Low | community |
| 110 | Zihan Dai, 2024  <https://doi.org/10.1016/j.advnut.2024.100262> | 19 | 568 | 4 weeks to 52 weeks | Time-Restricted Eating (TRE) combined with all types of exercise. TRE involves confining the eating window to a specified number of hours per day and fasting with zero-calorie beverages for the remaining hours | Control diet with the same exercise program. The control diet was described as a diet not following the TRE pattern (e.g., habitual diet, usual diet, non-TRE diet, normal dietary pattern) | Hong Kong, China; Japan; and the United Kingdom, Taiwan | Low | community |
| 111 | Ziyan Ding, 2025  <https://doi.org/10.1136/bmjopen-2024-091743> | 10 | 286 | 6 weeks to 12 weeks | Water Aerobics (WAs). The types of WAs included water aerobics, aqua Zumba, water yoga, and aqua jogging. The WAs generally occurred two to three times a week, 60 mins | Other exercise modalities or no exercise control | Malaysia, Brazil, India, the USA and the Netherlands | Low | Community |
| 112 | Jong Han Choi, 2022  <https://doi.org/10.4093/dmj.2022.0051> | 8 | 619 | 8 weeks to 24 weeks. | Moderately-low carbohydrate diet (mLCD): Carbohydrates are 26% to 45% of total caloric intake. Low carbohydrate diet (LCD): Carbohydrates are 10% to 25% of total caloric intake. Very-low carbohydrate diet (VLCD): Carbohydrates are less than 10% of total caloric intake. | Isocaloric control diets that were equivalent in total calories to the intervention diets. | United States, United Kingdom, Australia, Europe, Bahrain, Israel, China, Japan, and Taiwan | Critically low | Community |
