# Supplementary material for: Effectiveness of dietary, physical activity, and lifestyle interventions on weight and cardiometabolic outcomes in adults with overweight or obesity: an umbrella review of 112 systematic reviews and meta-analyses from 50 countries
Source: Obes Pillars. 2026 Sep 1;20:100324. doi: 10.1016/j.obpill.2026.100324 (PMC13571594; doi:10.1016/j.obpill.2026.100324)
Supplement: Supplementary file 6 [file mmc6.docx]

**Supplementary Table : list of excluded study with the reason for exclusion**

| **S.no** | **Author name** | **Reason for exclusion** | **S.no** | **Author name** | **Reason for exclusion** |
| --- | --- | --- | --- | --- | --- |
| 1 | Abbott, 2023 | not relevant to scope | 721 | Reynolds, 2020 | not relevant to scope |
| 2 | Abd Elqader, 2025 | not relevant to scope | 722 | Rezende, 2022 | irrelevant outcome |
| 3 | Abdallah, 2024 | ineligible population | 723 | Riaz, 2021 | not relevant to scope |
| 4 | Abdel Rahman, 2018 | ineligible population | 724 | Rico-González, 2024 | ineligible population |
| 5 | Abdelhamid, 2018 | not relevant to scope | 725 | Rico-González, 2025 | ineligible population |
| 6 | Abdelhamid, 2018 | not relevant to scope | 726 | Riediger, 2018 | not relevant to scope |
| 7 | Abdelhamid, 2020 | not relevant to scope | 727 | Rivera Regalado, 2024 | not relevant to scope |
| 8 | Abdollahi, 2020 | ineligible intervention | 728 | Rizk, 2020 | not relevant to scope |
| 9 | Abdulan, 2023 | irrelevant outcome | 729 | Robbins, 2015 | ineligible population |
| 10 | Abiri, 2025 | ineligible intervention | 730 | Robertson, 2024 | not relevant to scope |
| 11 | Aceves-Martins, 2016 | ineligible population | 731 | Robinson, 2022 | not relevant to scope |
| 12 | Acuff, 2019 | not relevant to scope | 732 | Rocha, 2025 | ineligible population |
| 13 | Adams, 2016 | not relevant to scope | 733 | Rodrigues-Krause, 2018 | not relevant to scope |
| 14 | Adom, 2019 | ineligible population | 734 | Rodriguez-Lopez, 2025 | ineligible population |
| 15 | Afshin, 2016 | not relevant to scope | 735 | Rog, 2024 | ineligible intervention |
| 16 | Agarwal, 2025 | not relevant to scope | 736 | Ronca, 2025 | not relevant to scope |
| 17 | Agrawal, 2023 | not relevant to scope | 737 | Rondanelli, 2024 | irrelevant outcome |
| 18 | Ahmadi, 2024 | not relevant to scope | 738 | Rondanelli, 2021 | not relevant to scope |
| 19 | Aiello, 2016 | not relevant to scope | 739 | Roomi, 2019 | not relevant to scope |
| 20 | Ait Hammou Taleb, 2023 | not relevant to scope | 740 | Ross, 2018 | not relevant to scope |
| 21 | Akingbuwa, 2020 | not relevant to scope | 741 | Roth, 2019 | not relevant to scope |
| 22 | Al Alawi, 2020 | not relevant to scope | 742 | Rousham, 2022 | ineligible population |
| 23 | Al-Khudairy, 2017 | ineligible population | 743 | Rubio-Arias, 2021 | not relevant to scope |
| 24 | Al-Mhanna, 2024 | not relevant to scope | 744 | Sara Paola Mambrini, 2023 | not relevant to scope |
| 25 | Al-Mhanna,2025 | not relevant to scope | 745 | Luke Wolfenden, 2018 | not relevant to scope |
| 26 | Albert Pérez, 2018 | ineligible population | 746 | [Tianrong Liao, 2024](https://pubmed.ncbi.nlm.nih.gov/?term=Miller+CT&cauthor_id=24409219) | not relevant to scope |
| 27 | Albulushi,2024 | not relevant to scope | 747 | Mohammed Alkharaiji, 2018 | not relevant to scope |
| 28 | Aldawsari, 2023 | ineligible intervention | 748 | Mansour Alblaji, 2025 | not relevant to scope |
| 29 | Aldekhail, 2015 | ineligible intervention | 749 | Han Shi Jocelyn Chew, 2023 | not relevant to scope |
| 30 | Aleid, 2024 | ineligible population | 750 | Ruiz-González, 2024 | ineligible intervention |
| 31 | Alenzi, 2023 | not relevant to scope | 751 | Russell, 2016 | ineligible population |
| 32 | Alfatafta, 2022 | not relevant to scope | 752 | Saad, 2022 | not relevant to scope |
| 33 | Alhindi, 2022 | not relevant to scope | 753 | Saeed, 2019 | not relevant to scope |
| 34 | Alhumaid, 2021 | not relevant to scope | 754 | Sagastume, 2024 | not relevant to scope |
| 35 | Aljawadi, 2024 | not relevant to scope | 755 | Salam, 2019 | not relevant to scope |
| 36 | Alkhathami, 2024 | not relevant to scope | 756 | Samdal, 2017 | not relevant to scope |
| 37 | Alkhunein, 2024 | irrelevant outcome | 757 | Sampath Kumar, 2019 | not relevant to scope |
| 38 | Allaf, 2021 | irrelevant outcome | 758 | Sanchez, 2022 | not relevant to scope |
| 39 | Allman-Farinelli, 2023 | not relevant to scope | 759 | Sánchez-de-la-Torre, 2023 | not relevant to scope |
| 40 | Alrubasy, 2024 | irrelevant outcome | 760 | Sánchez-Martínez, 2021 | not relevant to scope |
| 41 | Alshaarawy, 2015 | not relevant to scope | 761 | Sanders, 2021 | irrelevant outcome |
| 42 | Alshahrani, 2021 | ineligible population | 762 | Santa Cruz, 2025 | not relevant to scope |
| 43 | Alston, 2016 | not relevant to scope | 763 | Sarker, 2022 | not relevant to scope |
| 44 | Alves Junior, 2017 | ineligible population | 764 | Saunders, 2016 | ineligible population |
| 45 | Alyafei, 2025 | ineligible intervention | 765 | Sawicka, 2020 | not relevant to scope |
| 46 | Amadou, 2021 | not relevant to scope | 766 | Schaap, 2018 | ineligible population |
| 47 | Amerkamp, 2025 | not relevant to scope | 767 | Schlesinger, 2017 | not relevant to scope |
| 48 | Ananthapavan, 2022 | irrelevant outcome | 768 | Schmidt, 2020 | not relevant to scope |
| 49 | Anderson, 2021 | not relevant to scope | 769 | Schmucker, 2022 | not relevant to scope |
| 50 | Andersson, 2025 | not relevant to scope | 770 | Schoeppe, 2016 | not relevant to scope |
| 51 | Angawi, 2021 | ineligible population | 771 | Schöneburg, 2025 | not relevant to scope |
| 52 | Anselma, 2020 | ineligible population | 772 | Schönenberger, 2021 | not relevant to scope |
| 53 | Anusruti, 2020 | not relevant to scope | 773 | Schroor, 2024 | not relevant to scope |
| 54 | Appleton, 2018 | ineligible population | 774 | Scott-Sheldon, 2020 | ineligible population |
| 55 | Aqeel, 2020 | irrelevant outcome | 775 | Seffah, 2023 | not relevant to scope |
| 56 | Carukshi, 2016 | irrelevant outcome | 776 | Seid, 2024 | not relevant to scope |
| 57 | Chai, 2023 | irrelevant outcome | 777 | Seid, 2023 | not relevant to scope |
| 58 | Chami, 20-16 | irrelevant outcome | 778 | Semlitsch, 2019 | ineligible intervention |
| 59 | Chan, 2022 | ineligible population | 779 | Semnani-Azad, 2024 | not relevant to scope |
| 60 | Chaput, 2016 | ineligible population | 780 | Senkus, 2024 | not relevant to scope |
| 61 | Chaput, 2017 | ineligible population | 781 | Serralde-Zúñiga, 2019 | ineligible intervention |
| 62 | Chastin, 2015 | irrelevant outcome | 782 | Serrano, 2019 | ineligible population |
| 63 | Chastin, 2019 | not relevant to scope | 783 | Seyyedi, 2019 | ineligible population |
| 64 | Chatzi, 2024 | not relevant to scope | 784 | Shalimar, 2022 | not relevant to scope |
| 65 | Chau, 2018 | ineligible population | 785 | Shannon, 2020 | not relevant to scope |
| 66 | Chaudhary, 2020 | irrelevant outcome | 786 | Sharifi, 2021 | not relevant to scope |
| 67 | Chaudhry, 2016 | irrelevant outcome | 787 | Shariful Is, 2019 | not relevant to scope |
| 68 | Chavez, 2020 | ineligible population | 788 | Shen, 2022 | not relevant to scope |
| 69 | Chen, 2023 | not relevant to scope | 789 | Shepherd, 2017 | not relevant to scope |
| 70 | Chen, 2025 | not relevant to scope | 790 | Shiraseb, 2022 | not relevant to scope |
| 71 | Chen, 2025 | not relevant to scope | 791 | Shirvani, 2021 | not relevant to scope |
| 72 | Chen, 2025 | not relevant to scope | 792 | Shortland, 2023 | not relevant to scope |
| 73 | Chen, 2016 | ineligible population | 793 | Shrestha, 2025 | not relevant to scope |
| 74 | Chen, 2025 | not relevant to scope | 794 | Si, 2015 | not relevant to scope |
| 75 | Chen, 2021 | not relevant to scope | 795 | Siebenhofer, 2021 | irrelevant outcome |
| 76 | Chen, 2019 | not relevant to scope | 796 | Silva, 2019 | not relevant to scope |
| 77 | Cheng, 2025 | ineligible population | 797 | Simancas-Racines, 2025 | not relevant to scope |
| 78 | Cheng, 2023 | irrelevant outcome | 798 | Singh, 2020 | not relevant to scope |
| 79 | Cheng, 2024 | irrelevant outcome | 799 | Singh, 2024 | not relevant to scope |
| 80 | Cheung, 2024 | not relevant to scope | 800 | Sirico, 2018 | not relevant to scope |
| 81 | Chiavaroli, 2021 | not relevant to scope | 801 | Skea, 2019 | not relevant to scope |
| 82 | Chinvararak, 2021 | not relevant to scope | 802 | Smart, 2018 | not relevant to scope |
| 83 | Choi, 2022 | not relevant to scope | 803 | Smith, 2018 | ineligible intervention |
| 84 | Choi, 2020 | not relevant to scope | 804 | Smith, 2024 | ineligible population |
| 85 | Choi, 2025 | not relevant to scope | 805 | Smith, 2016 | not relevant to scope |
| 86 | Choi, 2020 | not relevant to scope | 806 | Smyth, 2017 | not relevant to scope |
| 87 | Choy, 2023 | not relevant to scope | 807 | Sochol, 2019 | not relevant to scope |
| 88 | Anne Hy, 2025 | not relevant to scope | 808 | Solmi, 2021 | not relevant to scope |
| 89 | Chu, 2021 | irrelevant outcome | 809 | Soltani, 2023 | not relevant to scope |
| 90 | Chua, 2024 | not relevant to scope | 810 | Soltani, 2025 | not relevant to scope |
| 91 | Churuangsuk, 2022 | not relevant to scope | 811 | Sones, 2025 | not relevant to scope |
| 92 | Clark, 2015 | full text not available | 812 | Soto-Mota, 2024 | not relevant to scope |
| 93 | Cloostermans, 2015 | irrelevant outcome | 813 | Spaans, 2025 | not relevant to scope |
| 94 | Coenen, 2024 | not relevant to scope | 814 | Spadaccini, 2022 | ineligible intervention |
| 95 | Coffey, 2020 | not relevant to scope | 815 | Spaulding, 2021 | not relevant to scope |
| 96 | Cohen, 2016 | not relevant to scope | 816 | Spanos, 2020 | ineligible population |
| 97 | Colak, 2025 | not relevant to scope | 817 | Sperkowska, 2021 | not relevant to scope |
| 98 | Coletta, 2019 | not relevant to scope | 818 | Spiga, 2024 | ineligible population |
| 99 | Collado-Soler, 2023 | ineligible population | 819 | Spiga, 2024 | ineligible population |
| 100 | Colquitt, 2016 | ineligible population | 820 | Spill, 2019 | ineligible population |
| 101 | Comeche, 2019 | not relevant to scope | 821 | Spracklen, 2017 | not relevant to scope |
| 102 | Conley, 2021 | not relevant to scope | 822 | Spreckley, 2021 | not relevant to scope |
| 103 | Cordella, 2021 | irrelevant outcome | 823 | St George, 2022 | ineligible population |
| 104 | Corder, 2020 | not relevant to scope | 824 | St Pierre, 2021 | ineligible population |
| 105 | Cormick, 2024 | not relevant to scope | 825 | Stanescu, 2019 | not relevant to scope |
| 106 | Correia, 2020 | not relevant to scope | 826 | Stelmach-Mardas, 2016 | not relevant to scope |
| 107 | Cortijo-Alfonso, 2024 | not relevant to scope | 827 | Stephan, 2024 | not relevant to scope |
| 108 | Costa, 2025 | ineligible population | 828 | Stringhini, 2017 | not relevant to scope |
| 109 | Couto Alves, 2019 | ineligible population | 829 | Strom, 2025 | not relevant to scope |
| 110 | Cradock, 2017 | not relevant to scope | 830 | Stubbs, 2016 | not relevant to scope |
| 111 | Craike, 2018 | not relevant to scope | 831 | Su, 2019 | not relevant to scope |
| 112 | Crider, 2022 | not relevant to scope | 832 | Su, 2024 | ineligible population |
| 113 | Cristiani, 2023 | not relevant to scope | 833 | Carlos Rodrigo Nascimento de Lira, 2023 | not relevant to scope |
| 114 | Cross, 2025 | not relevant to scope | 834 | Zhicheng Guo, 2023 | not relevant to scope |
| 115 | Crowder, 2022 | ineligible population | 835 | Zikang Hao , 2023 | not relevant to scope |
| 116 | Cruz-Cobo, 2020 | not relevant to scope | 836 | Harry M. Hays, 2025 | not relevant to scope |
| 117 | Cuijpers, 2023 | not relevant to scope | 837 | Beatrice Herzog, 2016 | not relevant to scope |
| 118 | Cunha, 2023 | not relevant to scope | 838 | Suchdev, 2020 | ineligible population |
| 119 | da Silva, 2022 | not relevant to scope | 839 | Suen, 2020 | not relevant to scope |
| 120 | da Silva Cunha de Medeiros, 2021 | ineligible population | 840 | Sun, 2021 | not relevant to scope |
| 121 | da Silva Gonçalves, 2023 | irrelevant outcome | 841 | Sushames, 2016 | not relevant to scope |
| 122 | Dabravolskaj, 2020 | not relevant to scope | 842 | Suzuki, 2021 | not relevant to scope |
| 123 | Dai, 2024 | not relevant to scope | 843 | Sylivris, 2022 | ineligible intervention |
| 124 | Dalrymple, 2018 | ineligible population | 844 | Tadayon Najafabadi, 2023 | not relevant to scope |
| 125 | De Amicis, 2022 | not relevant to scope | 845 | Tallamraju, 2021 | not relevant to scope |
| 126 | De Amicis, 2022 | ineligible population | 846 | Talukdar, 2024 | not relevant to scope |
| 127 | De Luca, 2025 | ineligible population | 847 | Tan, 2022 | not relevant to scope |
| 128 | de Mello, 2024 | ineligible population | 848 | Tangvik, 2021 | not relevant to scope |
| 129 | De Pasquale, 2023 | not relevant to scope | 849 | Tankeu, 2023 | irrelevant outcome |
| 130 | de Vries, 2019 | not relevant to scope | 850 | Tanous, 2025 | ineligible population |
| 131 | Dewey, 2021 | ineligible population | 851 | Tarp, 2022 | not relevant to scope |
| 132 | Dharmayani, 2021 | not relevant to scope | 852 | Tarp, 2019 | not relevant to scope |
| 133 | Díaz-García, 2021 | not relevant to scope | 853 | Taylor, 2021 | not relevant to scope |
| 134 | Díaz-López, 2023 | not relevant to scope | 854 | Tenorio-Jiménez, 2020 | not relevant to scope |
| 135 | Dicken, 2024 | irrelevant outcome | 855 | Ter Beek, 2021 | not relevant to scope |
| 136 | Dicks, 2022 | not relevant to scope | 856 | Teumer, 2016 | not relevant to scope |
| 137 | Ding, 2022 | not relevant to scope | 857 | Thaker, 2018 | not relevant to scope |
| 138 | Dipietro, 2019 | not relevant to scope | 858 | Theodoulou, 2023 | not relevant to scope |
| 139 | Dira, 2025 | ineligible population | 859 | Thiboonboon, 2024 | not relevant to scope |
| 140 | Dobrow, 2021 | not relevant to scope | 860 | Thinakaran, 2025 | not relevant to scope |
| 141 | Dominguez-Antuña, 2023 | not relevant to scope | 861 | Thind, 2017 | not relevant to scope |
| 142 | Donazar-Ezcurra, 2017 | not relevant to scope | 862 | Thu, 2023 | not relevant to scope |
| 143 | Dong, 2023 | not relevant to scope | 863 | Tieland, 2017 | not relevant to scope |
| 144 | Donini, 2020 | not relevant to scope | 864 | Tieu, 2017 | not relevant to scope |
| 145 | Dos Santos, 2020 | not relevant to scope | 865 | Tieu, 2024 | not relevant to scope |
| 146 | Duarte, 2022 | not relevant to scope | 866 | Timmermans, 2020 | not relevant to scope |
| 147 | Dudzik, 2023 | not relevant to scope | 867 | Tobias, 2023 | not relevant to scope |
| 148 | Duerinckx, 2016 | not relevant to scope | 868 | Tobias, 2025 | not relevant to scope |
| 149 | Durao, 2020 | not relevant to scope | 869 | Tomayko, 2021 | not relevant to scope |
| 150 | Durao, 2020 | not relevant to scope | 870 | Torabi, 2024 | not relevant to scope |
| 151 | Dziegielewska-Gesiak, 2021 | not relevant to scope | 871 | Tornese, 2025 | ineligible population |
| 152 | Ebireri, 2016 | not relevant to scope | 872 | Torossian, 2021 | not relevant to scope |
| 153 | Carolyn C., 2025 | not relevant to scope | 873 | Trabelsi, 2020 | ineligible population |
| 154 | Ehret, 2016 | not relevant to scope | 874 | Trezia, 2025 | ineligible population |
| 155 | Ejtahed, 2024 | not relevant to scope | 875 | Trieu, 2021 | not relevant to scope |
| 156 | Ekambareshwar, 2021 | ineligible population | 876 | Trivedi, 2024 | not relevant to scope |
| 157 | El Archi, 2020 | not relevant to scope | 877 | Tsai, 2020 | not relevant to scope |
| 158 | Elfar, 2024 | not relevant to scope | 878 | Tsega, 20256 | not relevant to scope |
| 159 | Elmajnoun, 2023 | not relevant to scope | 879 | Tukanov, 2024 | not relevant to scope |
| 160 | Elmesmari, 2018 | ineligible population | 880 | Tully, 2021 | ineligible population |
| 161 | Emadian, 2015 | not relevant to scope | 881 | Turicchi, 2019 | not relevant to scope |
| 162 | Laural K., 2019 | ineligible population | 882 | Turton, 2018 | not relevant to scope |
| 163 | Enricho Nkhoma, 2021 | not relevant to scope | 883 | Ukke, 2023 | not relevant to scope |
| 164 | Enright, 2020 | ineligible population | 884 | Utz-Melere, 2018 | ineligible population |
| 165 | Erlich, 2024 | not relevant to scope | 885 | Vajdi, 2024 | not relevant to scope |
| 166 | Escribano-Ardura, 2020 | not relevant to scope | 886 | Vajdi, 2024 | not relevant to scope |
| 167 | Esmaeilinezhad, 2025 | not relevant to scope | 887 | Valentín-Gudiol, 2017 | ineligible population |
| 168 | Espinosa, 2024 | ineligible population | 888 | Vallabhan, 2018 | ineligible population |
| 169 | Fakhraei, 2022 | not relevant to scope | 889 | van Abswoude, 2023 | irrelevant outcome |
| 170 | Falcone, 2024 | not relevant to scope | 890 | van den Heuvel, 2024 | ineligible intervention |
| 171 | Fang, 2016 | not relevant to scope | 891 | van Gils, 2024 | irrelevant outcome |
| 172 | Farooq, 2020 | ineligible population | 892 | Van Roessel, 2024 | irrelevant outcome |
| 173 | Fatima, 2024 | not relevant to scope | 893 | van Zutphen, 2025 | irrelevant outcome |
| 174 | Feinberg, 2017 | not relevant to scope | 894 | Vander Wyst, 2021 | ineligible population |
| 175 | Feitosa, 2018 | not relevant to scope | 895 | Vanderhout, 2020 | ineligible population |
| 176 | Feng, 2025 | not relevant to scope | 896 | Varela-López, 2018 | ineligible intervention |
| 177 | Feng, 2025 | irrelevant outcome | 897 | Vargas, 2018 | irrelevant outcome |
| 178 | Fernández-Lázaro, 2024 | ineligible population | 898 | Varhlunchungi, 2023 | ineligible population |
| 179 | Fisher, 2018 | not relevant to scope | 899 | Varkevisser, 2019 | inappropriate study design |
| 180 | Caroline A., 2019 | not relevant to scope | 900 | Vassou, 2020 | inappropriate study design |
| 181 | Fitch, 2021 | ineligible intervention | 901 | Vaughan, 2024 | ineligible population |
| 182 | Fong, 2023 | not relevant to scope | 902 | Vedder, 2019 | irrelevant outcome |
| 183 | Fontvieille, 2023 | not relevant to scope | 903 | Vedrenne-Gutiérrez, 2024 | inappropriate study design |
| 184 | Foong, 2020 | ineligible intervention | 904 | Vehmeijer, 2020 | ineligible population |
| 185 | Frączek, 2021 | not relevant to scope | 905 | Vescio, 2021 | inappropriate study design |
| 186 | Franchi, 2025 | not relevant to scope | 906 | Vieira, 2022 | irrelevant outcome |
| 187 | Freak-Poli, 2020 | not relevant to scope | 907 | Visser, 2018 | ineligible intervention |
| 188 | Frerichs, 2016 | ineligible population | 908 | Vogelezang, 2018 | inappropriate study design |
| 189 | Fretts, 2015 | not relevant to scope | 909 | Voorn, 2022 | irrelevant outcome |
| 190 | Fu, 2020 | not relevant to scope | 910 | Vrieling, 2018 | irrelevant outcome |
| 191 | Fu, 2021 | not relevant to scope | 911 | Waite, 2024 | irrelevant outcome |
| 192 | Fühner, 2021 | ineligible population | 912 | Wan, 2024 | irrelevant outcome |
| 193 | Fuhrman, 2021 | not relevant to scope | 913 | Wang and Boros, 2025 | irrelevant outcome |
| 194 | Furthner, 2021 | not relevant to scope | 914 | Wang, 2023 | irrelevant outcome |
| 195 | Gallego Sobrino, 2021 | ineligible population | 915 | Wang, 2022 | irrelevant outcome |
| 196 | Gao, 2019 | ineligible intervention | 916 | Wang, 2025 | irrelevant outcome |
| 197 | Garbers, 2018 | not relevant to scope | 917 | Wang, 2020 | irrelevant outcome |
| 198 | Garcia, 2024 | not relevant to scope | 918 | Wang, 2020 | irrelevant outcome |
| 199 | Garcia-Arellano, 2019 | not relevant to scope | 919 | Wang, 2022 | ineligible population |
| 200 | García-Hermoso, 2020 | not relevant to scope | 920 | Wang, 2022 | irrelevant outcome |
| 201 | Garrido-Miguel, 2019 | ineligible population | 921 | Wang, 2018 | irrelevant outcome |
| 202 | Garrido-Miguel, 2019 | ineligible population | 922 | Wang, 2019 | irrelevant outcome |
| 203 | Garshick, 2019 | not relevant to scope | 923 | Wang-Chen, 2025 | inappropriate study design |
| 204 | Gasparri, 2019 | not relevant to scope | 924 | Watson, 2017 | ineligible population |
| 205 | Geerling, 2022 | not relevant to scope | 925 | Watson, 2020 | irrelevant outcome |
| 206 | George, 2022 | not relevant to scope | 926 | Watson, 2023 | irrelevant outcome |
| 207 | Ghazzawi, 2023 | ineligible population | 927 | Webb, 2021 | irrelevant outcome |
| 208 | Ghiotto, 2022 | ineligible intervention | 928 | Wei, 2025 | irrelevant outcome |
| 209 | Ghomrawi, 2020 | not relevant to scope | 929 | Wei, 2025 | irrelevant outcome |
| 210 | Gibbs, 2017 | not relevant to scope | 930 | Wei, 2025 | irrelevant outcome |
| 211 | Gielen, 2021 | not relevant to scope | 931 | Weihrauch-Blüher, 2018 | inappropriate study design |
| 212 | Gindri, 2024 | not relevant to scope | 932 | Werneck, 2020 | ineligible population |
| 213 | Golubovic, 2022 | not relevant to scope | 933 | Wharton, 2019 | irrelevant outcome |
| 214 | Gomes, 2021 | not relevant to scope | 934 | Wherry, 2021 | irrelevant outcome |
| 215 | González-Gómez, 2025 | not relevant to scope | 935 | Whitehall, 2025 | irrelevant outcome |
| 216 | Goodyear, 2021 | not relevant to scope | 936 | Whittaker, 2022 | irrelevant outcome |
| 217 | Gori, 2017 | ineligible population | 937 | Wick, 2017 | ineligible population |
| 218 | Grabia, 2021 | ineligible population | 938 | Wierzbicka, 2022 | irrelevant outcome |
| 219 | Grace, 2017 | not relevant to scope | 939 | Wiggins, 2020 | irrelevant outcome |
| 220 | Gracia-Sánchez, 2025 | not relevant to scope | 940 | Wilkins, 2021 | irrelevant outcome |
| 221 | Grady, 2025 | ineligible population | 941 | Williams, 2019 | irrelevant outcome |
| 222 | Greaney, 2023 | not relevant to scope | 942 | Willmott, 2019 | inappropriate study design |
| 223 | Griffin, 2021 | ineligible intervention | 943 | Wimalawansa, 2023 | irrelevant outcome |
| 224 | Groenendijk, 2019 | not relevant to scope | 944 | Wimalawansa, 2025 | irrelevant outcome |
| 225 | Grummon, 2020 | not relevant to scope | 945 | Winkler, 2015 | irrelevant outcome |
| 226 | Guest, 2024 | not relevant to scope | 946 | Winkley, 2020 | irrelevant outcome |
| 227 | Guillem, 2020 | not relevant to scope | 947 | Wirth, 2020 | irrelevant outcome |
| 228 | Guo, 2021 | not relevant to scope | 948 | Wolf, 2020 | ineligible population |
| 229 | Kapil gupta, 2018 | not relevant to scope | 949 | Wolfenden, 2018 | irrelevant outcome |
| 230 | Gutermuth, 2018 | not relevant to scope | 950 | Wong, 2022 | irrelevant outcome |
| 231 | Gutierrez, 2021 | not relevant to scope | 951 | Woodruff, 2018 | irrelevant outcome |
| 232 | Habaybeh, 2021 | not relevant to scope | 952 | Woods, 2022 | ineligible population |
| 233 | Deirdre, 2018 | not relevant to scope | 953 | Woolhiser, 2024 | irrelevant outcome |
| 234 | Deirdre, 2020 | not relevant to scope | 954 | Woolley, 2025 | irrelevant outcome |
| 235 | Halilagic, 2021 | ineligible population | 955 | Nan Lv, 2017 | not relevant to scope |
| 236 | Hall, 2020 | not relevant to scope | 956 | Abbas Mohtashamian, 2025 | not relevant to scope |
| 237 | Hammersley, 2016 | ineligible population | 957 | Marie H. Murphy, 2019 | not relevant to scope |
| 238 | Hamsho, 2025 | not relevant to scope | 958 | Michael Nunns, 2025 | not relevant to scope |
| 239 | Harman, 2017 | not relevant to scope | 959 | Eric Tsz-Chun Poon, 2024 | not relevant to scope |
| 240 | Harrison, 2023 | ineligible population | 960 | Victoria Sandercock, 2018 | not relevant to scope |
| 241 | Hartmann-Boyce, 2018 | not relevant to scope | 961 | Thomas Semlitsch, 2016 | not relevant to scope |
| 242 | Hartmann-Boyce, 2023 | not relevant to scope | 962 | A. Sherrington, 2016 | not relevant to scope |
| 243 | Harvey, 2021 | not relevant to scope | 963 | Gaofeng Wang, 2025 | not relevant to scope |
| 244 | Hasan, 2018 | not relevant to scope | 964 | Yue-Heng Yin, 2021 | not relevant to scope |
| 245 | Hassanein, 2023 | not relevant to scope | 965 | Raquel Canuto, 2017 | not relevant to scope |
| 246 | Hassapidou, 2023 | not relevant to scope | 966 | Han-Na Kim, 2019 | not relevant to scope |
| 247 | Hayba, 2020 | ineligible population | 967 | Wrench, 2022 | irrelevant outcome |
| 248 | Hayden, 2020 | not relevant to scope | 968 | Wright, 2019 | irrelevant outcome |
| 249 | Mingqian, 2024 | not relevant to scope | 969 | Wu, 2017 | irrelevant outcome |
| 250 | Heenan, 2020 | not relevant to scope | 970 | Wu, 2016 | irrelevant outcome |
| 251 | Hengeveld, 2020 | not relevant to scope | 971 | Wu, 2025 | irrelevant outcome |
| 252 | Hengeveld, 2022 | not relevant to scope | 972 | Wu, 2023 | ineligible intervention |
| 253 | Heron, 2017 | not relevant to scope | 973 | Xie, 2022 | not relevant to scope |
| 254 | Herrmann, 2024 | not relevant to scope | 974 | Xie, 2023 | not relevant to scope |
| 255 | Hilgenkam, 2025 | not relevant to scope | 975 | Xie, 2022 | not relevant to scope |
| 256 | Hincapié, 2025 | not relevant to scope | 976 | Xie, 2023 | not relevant to scope |
| 257 | Hironaka, 2024 | not relevant to scope | 977 | Xiong, 2016 | not relevant to scope |
| 258 | Hobbins, 2017 | not relevant to scope | 978 | Xu, 2018 | not relevant to scope |
| 259 | Hodder, 2022 | ineligible population | 979 | Xu, 2025 | not relevant to scope |
| 260 | Hodkinson, 2021 | not relevant to scope | 980 | Xu, 2025 | not relevant to scope |
| 261 | Hodkinson, 2025 | not relevant to scope | 981 | Yamazaki, 2016 | not relevant to scope |
| 262 | Holtjer, 2023 | not relevant to scope | 982 | Yan, 2018 | not relevant to scope |
| 263 | Hong, 2018 | not relevant to scope | 983 | Yan, 2025 | not relevant to scope |
| 264 | Hong, 2018 | not relevant to scope | 984 | Yan, 2025 | not relevant to scope |
| 265 | Hooper, 2018 | not relevant to scope | 985 | Yan, 2016 | not relevant to scope |
| 266 | Hooper, 2018 | not relevant to scope | 986 | Yang, 2021 | not relevant to scope |
| 267 | Hooper, 2020 | not relevant to scope | 987 | Yang, 2023 | not relevant to scope |
| 268 | Hooper, 2020 | not relevant to scope | 988 | Yang, 2022 | ineligible population |
| 269 | Hope, 2019 | not relevant to scope | 989 | Yang, 2019 | not relevant to scope |
| 270 | Horikoshi, 2016 | not relevant to scope | 990 | Yang, 2022 | not relevant to scope |
| 271 | Hsu, 2020 | not relevant to scope | 991 | Yang, 2022 | not relevant to scope |
| 272 | Hsu, 2025 | not relevant to scope | 992 | Yau, 2022 | ineligible population |
| 273 | Donglin, 2021 | ineligible population | 993 | Ye, 2021 | not relevant to scope |
| 274 | Huang, 2016 | not relevant to scope | 994 | Beatrice Herzog, 2016 | not relevant to scope |
| 275 | Huang, 2024 | not relevant to scope | 995 | Karen Jiang, 2022 | not relevant to scope |
| 276 | Huang, 2024 | not relevant to scope | 996 | Joseph MS, 2019 | not relevant to scope |
| 277 | Hudda, 2019 | ineligible population | 997 | Zhijian He, 2023 | not relevant to scope |
| 278 | Hudda, 2022 | ineligible population | 998 | Kworweinski Lafontant, 2025 | not relevant to scope |
| 279 | Hudson, 2023 | ineligible population | 999 | Yerrakalva, 2015 | not relevant to scope |
| 280 | Hughes, 2017 | ineligible population | 1000 | Yeung, 2025 | not relevant to scope |
| 281 | Hung, 2022 | not relevant to scope | 1001 | Yin, 2023 | not relevant to scope |
| 282 | Hurst, 2024 | not relevant to scope | 1002 | Yip, 2016 | ineligible population |
| 283 | Hurwitz, 2022 | not relevant to scope | 1003 | Yiu, 2023 | not relevant to scope |
| 284 | huynh, 2024 | not relevant to scope | 1004 | Yokote, 2024 | ineligible intervention |
| 285 | Hwang, 2022 | not relevant to scope | 1005 | Yoon, 2020 | not relevant to scope |
| 286 | Ibeneme, 2024 | not relevant to scope | 1006 | Yoong, 2020 | ineligible population |
| 287 | Imamura, 2018 | not relevant to scope | 1007 | Yoong, 2023 | ineligible population |
| 288 | Imamura, 2016 | not relevant to scope | 1008 | Yu, 2017 | not relevant to scope |
| 289 | Imani, 2021 | not relevant to scope | 1009 | Yuan, 2021 | not relevant to scope |
| 290 | Imani, 2022 | not relevant to scope | 1010 | Zanker, 2023 | not relevant to scope |
| 291 | Inolopú, 2019 | not relevant to scope | 1011 | Zarnowska, 2025 | not relevant to scope |
| 292 | Ipema, 2016 | not relevant to scope | 1012 | Zeleke, 2025 | not relevant to scope |
| 293 | Iqbal, 2021 | not relevant to scope | 1013 | Zhang, 2023 | ineligible intervention |
| 294 | M. Horne, 2020 | ineligible intervention | 1014 | Zhang, 2020 | not relevant to scope |
| 295 | Huang, Tongjiang MM, 2025 | ineligible intervention | 1015 | Zhang, 2023 | not relevant to scope |
| 296 | Yaltafit Abror Jeem, 2022 | ineligible intervention | 1016 | Zhang, 2021 | not relevant to scope |
| 297 | Karen Jiang, 2022 | ineligible intervention | 1017 | Zhao, 2016 | not relevant to scope |
| 298 | Irfan, 2020 | not relevant to scope | 1018 | Zheng, 2024 | ineligible population |
| 299 | Jackson, 2021 | ineligible population | 1019 | Zheng, 2023 | not relevant to scope |
| 300 | Jacob, 2021 | ineligible population | 1020 | Zhou, 2019 | not relevant to scope |
| 301 | Jafari, 2025 | not relevant to scope | 1021 | Zhu, 2016 | not relevant to scope |
| 302 | James-Martin, 2024 | not relevant to scope | 1022 | Zhuang, 2020 | not relevant to scope |
| 303 | Jamka, 2015 | not relevant to scope | 1023 | Ziegeldorf, 2020 | not relevant to scope |
| 304 | Janković, 2021 | not relevant to scope | 1024 | Zika, 2021 | not relevant to scope |
| 305 | Jansen, 2018 | not relevant to scope | 1025 | Ziora-Jakutowicz, 2021 | not relevant to scope |
| 306 | Jansen, 2019 | not relevant to scope | 1026 | Zohara, 2023 | not relevant to scope |
| 307 | Jarvis, 2016 | not relevant to scope | 1027 | Zupo, 2022 | not relevant to scope |
| 308 | Jawad, 2019 | not relevant to scope | 1028 | Aline Andres , 2024 | not relevant to scope |
| 309 | Jebeile, 2019 | ineligible population | 1029 | Araújo, 2024 | not relevant to scope |
| 310 | Jebeile, 2023 | irrelevant outcome | 1030 | Arisi, 2024 | not relevant to scope |
| 311 | Jenum, 2019 | not relevant to scope | 1031 | Armitage, 2024 | not relevant to scope |
| 312 | Jibril, 2022 | ineligible intervention | 1032 | Arno, 2016 | not relevant to scope |
| 313 | Jiménez-Orenga, 2025 | not relevant to scope | 1033 | Arnold, 2016 | not relevant to scope |
| 314 | Jimenez-Pavon, 2019 | not relevant to scope | 1034 | Arora, 2019 | not relevant to scope |
| 315 | Johnson, 2018 | not relevant to scope | 1035 | Arrona-Cardoza, 2023 | not relevant to scope |
| 316 | Johnson-Mann, 2023 | ineligible intervention | 1036 | Arsenis, 2017 | not relevant to scope |
| 317 | Johnston, 2022 | not relevant to scope | 1037 | Ash, 2017 | ineligible population |
| 318 | Johnston, 2017 | not relevant to scope | 1038 | Asher, 2025 | not relevant to scope |
| 319 | Jones, 2021 | irrelevant outcome | 1039 | Ashgar, 2025 | not relevant to scope |
| 320 | Jonkman, 2016 | not relevant to scope | 1040 | Aubertin-Leheudre, 2020 | not relevant to scope |
| 321 | Joubert, 2016 | not relevant to scope | 1041 | Aune, 2020 | not relevant to scope |
| 322 | Jurek, 2025 | not relevant to scope | 1042 | Austin, 2021 | not relevant to scope |
| 323 | Justice, 2019 | not relevant to scope | 1043 | Avenell, 2018 | ineligible intervention |
| 324 | Justice, 2017 | ineligible intervention | 1044 | Ayalew, 2023 | not relevant to scope |
| 325 | Kadalayil, 2023 | not relevant to scope | 1045 | Baguley, 2025 | not relevant to scope |
| 326 | Kaewdech, 2022 | not relevant to scope | 1046 | Bai, 2021 | not relevant to scope |
| 327 | Kalamajski, 2022 | not relevant to scope | 1047 | Baillot, 2021 | not relevant to scope |
| 328 | Kampmeijer, 2016 | not relevant to scope | 1048 | Baker, 2025 | not relevant to scope |
| 329 | Kang, 2019 | ineligible population | 1049 | Banany, 2024 | ineligible population |
| 330 | Kapała, 2025 | ineligible intervention | 1050 | Banna, 2018 | not relevant to scope |
| 331 | Karimi, 2025 | not relevant to scope | 1051 | Baradaran Mahdavi, 2021 | not relevant to scope |
| 332 | Karimi, 2024 | not relevant to scope | 1052 | Barata, 2017 | not relevant to scope |
| 333 | Karimijashni, 2025 | not relevant to scope | 1053 | Barnett, 2016 | not relevant to scope |
| 334 | Karyotaki, 2017 | not relevant to scope | 1054 | Baroncini, 2022 | not relevant to scope |
| 335 | Kashyap, 2022 | not relevant to scope | 1055 | Baroncini, 2024 | not relevant to scope |
| 336 | Kazeminasab, 2025 | irrelevant outcome | 1056 | Barrett, 2018 | not relevant to scope |
| 337 | Keller, 2020 | not relevant to scope | 1057 | Barrow, 2019 | irrelevant outcome |
| 338 | Kelly, 2016 | not relevant to scope | 1058 | Bartels, 2016 | not relevant to scope |
| 339 | Kemmler, 2021 | not relevant to scope | 1059 | Batsis, 2021 | irrelevant outcome |
| 340 | M. Horne, 2020 | not relevant to scope | 1060 | Battista, 2021 | not relevant to scope |
| 341 | Fatemeh Sadat Hashemi Javaheri, 2025 | not relevant to scope | 1061 | Bavuma, 2019 | not relevant to scope |
| 342 | JAKICIC, 2019 | not relevant to scope | 1062 | Baygi, 2023 | ineligible population |
| 343 | Karen Jiang, 2022 | not relevant to scope | 1063 | Beaulieu, 2021 | not relevant to scope |
| 344 | Joseph MS, 2019 | not relevant to scope | 1064 | Beavers, 2025 | not relevant to scope |
| 345 | Yaltafit Abror Jeem, 2022 | not relevant to scope | 1065 | Bei, 2016 | not relevant to scope |
| 346 | Wei Liang, 2024 | not relevant to scope | 1066 | Beishuizen, 2016 | not relevant to scope |
| 347 | Liao Chun-De, 2017 | not relevant to scope | 1067 | Bellicha, 2021 | not relevant to scope |
| 348 | Ken-Dror, 2024 | not relevant to scope | 1068 | Bellicha, 2021 | ineligible intervention |
| 349 | Kenkhuis, 2025 | not relevant to scope | 1069 | Bellissimo, 2018 | not relevant to scope |
| 350 | Kenneally, 2017 | not relevant to scope | 1070 | Bengtsson, 2025 | not relevant to scope |
| 351 | Kennedy, 2019 | not relevant to scope | 1071 | Bennett, 2018 | duplicate |
| 352 | Kent, 2021 | not relevant to scope | 1072 | Bentley, 2019 | not relevant to scope |
| 353 | Kent, 2021 | not relevant to scope | 1073 | Bergamini, 2022 | not relevant to scope |
| 354 | Kermansaravi, 2023 | ineligible intervention | 1074 | Berkley-Patton, 2020 | not relevant to scope |
| 355 | Kerrison, 2017 | not relevant to scope | 1075 | Bertoia, 2016 | not relevant to scope |
| 356 | Khadra, 2019 | not relevant to scope | 1076 | Bibby, 2024 | not relevant to scope |
| 357 | Khatib, 2018 | not relevant to scope | 1077 | Birch, 2022 | irrelevant outcome |
| 358 | Khodarahmi, 2025 | not relevant to scope | 1078 | Birch, 2019 | ineligible population |
| 359 | Kibret, 2025 | irrelevant outcome | 1079 | Bischof, 2024 | not relevant to scope |
| 360 | Kienlein, 2025 | not relevant to scope | 1080 | Bishnoi, 2022 | not relevant to scope |
| 361 | Kiesswetter, 2023 | not relevant to scope | 1081 | Bleich, 2017 | not relevant to scope |
| 362 | Kim, 2021 | not relevant to scope | 1082 | Boden, 2024 | not relevant to scope |
| 363 | Kirchin, 2017 | not relevant to scope | 1083 | Bohn-Goldbaum, 2022 | not relevant to scope |
| 364 | Kite, 2019 | not relevant to scope | 1084 | Bohr, 2016 | not relevant to scope |
| 365 | Klassen, 2018 | not relevant to scope | 1085 | Bonvicini, 2022 | ineligible population |
| 366 | Klimas, 2018 | not relevant to scope | 1086 | Bora, 2024 | irrelevant outcome |
| 367 | Klingman, 2017 | not relevant to scope | 1087 | Bösch, 2024 | not relevant to scope |
| 368 | Klonizakis, 2021 | not relevant to scope | 1088 | Bosu, 2019 | not relevant to scope |
| 369 | Klos, 2023 | ineligible population | 1089 | Boushey, 2020 | not relevant to scope |
| 370 | Shu-Hua, 2019 | not relevant to scope | 1090 | Bouzas, 2019 | not relevant to scope |
| 371 | Kocarnik, 2022 | not relevant to scope | 1091 | Bowers, 2023 | not relevant to scope |
| 372 | Koch, 2024 | not relevant to scope | 1092 | Braam, 2016 | not relevant to scope |
| 373 | Kong, 2024 | not relevant to scope | 1093 | Bradfield, 2019 | ineligible population |
| 374 | Kourounis, 2020 | ineligible intervention | 1094 | Brauer, 2021 | not relevant to scope |
| 375 | Koutaki, 2025 | ineligible population | 1095 | Bredin, 2021 | not relevant to scope |
| 376 | kouvari, 2022 | ineligible population | 1096 | Breuil-Marsal, 2024 | not relevant to scope |
| 377 | Kovács, 2022 | ineligible population | 1097 | Bridger Staatz, 2021 | ineligible population |
| 378 | Kozuki, 2015 | not relevant to scope | 1098 | Bridgwood, 2018 | not relevant to scope |
| 379 | Kraus, 2019 | not relevant to scope | 1099 | Brigden, 2020 | ineligible population |
| 380 | Kraus, 2019 | not relevant to scope | 1100 | Bril, 2023 | not relevant to scope |
| 381 | Krebs, 2022 | not relevant to scope | 1101 | Brown, 2017 | not relevant to scope |
| 382 | Krijger, 2022 | ineligible population | 1102 | Brown, 2024 | not relevant to scope |
| 383 | Kudlek, 2025 | not relevant to scope | 1103 | Brown, 2019 | ineligible population |
| 384 | Kuhle, 2020 | not relevant to scope | 1104 | Brownley, 2016 | not relevant to scope |
| 385 | Kumbargere Nagraj, 2017 | not relevant to scope | 1105 | Buckland, 2018 | irrelevant outcome |
| 386 | Kuo, 2022 | not relevant to scope | 1106 | Buffart, 2017 | not relevant to scope |
| 387 | Küpers, 2019 | not relevant to scope | 1107 | Bulmer, 2025 | not relevant to scope |
| 388 | Kuznia, 2023 | not relevant to scope | 1108 | Burden, 2024 | not relevant to scope |
| 389 | Kyrgiou, 2017 | not relevant to scope | 1109 | Burden, 2019 | not relevant to scope |
| 390 | Lafortune, 2016 | not relevant to scope | 1110 | Bushey, 2024 | not relevant to scope |
| 391 | Lagou, 2021 | not relevant to scope | 1111 | Butscher, 2024 | ineligible population |
| 392 | Rizwana, 2017 | not relevant to scope | 1112 | Byun, 2022 | not relevant to scope |
| 393 | Lam, 2022 | ineligible population | 1113 | Caldwell, 2024 | not relevant to scope |
| 394 | Lane, 2016 | ineligible population | 1114 | Camañes-Gonzalvo, 2022 | not relevant to scope |
| 395 | Lange, 2023 | not relevant to scope | 1115 | Cammaroto, 2025 | not relevant to scope |
| 396 | Larsen, 2019 | not relevant to scope | 1116 | Campbell, 2019 | not relevant to scope |
| 397 | Laskar, 2024 | not relevant to scope | 1117 | Canfell, 2018 | not relevant to scope |
| 398 | Wa'ed Shiyab, 2023 | ineligible intervention | 1118 | Caponnetto, 2021 | not relevant to scope |
| 399 | Elena Sosa-Cordobés, 2022 | ineligible intervention | 1119 | Caputo, 2023 | irrelevant outcome |
| 400 | Lawrence, 2016 | not relevant to scope | 1120 | Carabotti, 2025 | not relevant to scope |
| 401 | Le, 2025 | not relevant to scope | 1121 | Carino, 2025 | not relevant to scope |
| 402 | Leavy, 2018 | not relevant to scope | 1122 | Carino, 2025 | not relevant to scope |
| 403 | Lee, 2019 | not relevant to scope | 1123 | Caristia, 2020 | not relevant to scope |
| 404 | Lee, 2025 | ineligible population | 1124 | Carlin, 2016 | ineligible population |
| 405 | Lee, 2024 | not relevant to scope | 1125 | Caron, 2022 | ineligible population |
| 406 | Lee-Bravatti, 2019 | not relevant to scope | 1126 | Carraça, 2021 | not relevant to scope |
| 407 | Leermakers, 2016 | not relevant to scope | 1127 | Carraça, 2021 | irrelevant outcome |
| 408 | Lemstra, 2021 | not relevant to scope | 1128 | Carrasco-Barrios, 2020 | not relevant to scope |
| 409 | Lentejas, 2024 | not relevant to scope | 1129 | Carson, 2016 | not relevant to scope |
| 410 | Leslie, 2023 | not relevant to scope | 1130 | Carter, 2024 | not relevant to scope |
| 411 | Letarouilly, 2020 | not relevant to scope | 1131 | Ken-Dror, 2024 | not relevant to scope |
| 412 | Li, 2022 | ineligible population | 1132 | Kenkhuis, 2025 | not relevant to scope |
| 413 | Li, 2017 | not relevant to scope | 1133 | Kenneally, 2017 | not relevant to scope |
| 414 | Li, 2023 | not relevant to scope | 1134 | Keller, 2020 | not relevant to scope |
| 415 | Li, 2025 | not relevant to scope | 1135 | Patricia Gálvez Espinoza, 2019 | non-english language |
| 416 | Li, 2021 | not relevant to scope | 1136 | Gielen Marij, 2018 | not relevant to scope |
| 417 | Li, 2024 | not relevant to scope | 1137 | Mariaelisa Graff , 2020 | ineligible intervention |
| 418 | Li, 2021 | not relevant to scope | 1138 | Kingsley O. Ozojide, 2025 | ineligible intervention |
| 419 | Li, 2024 | ineligible population | 1139 | Alberto Verrotti, 2014 | irrelevant outcome |
| 420 | Li, 2025 | not relevant to scope | 1140 | Lisa R Staimez, 2013 | inappropriate study design |
| 421 | Li, 2023 | not relevant to scope | 1141 | Yunhwan Lee, 2010 | irrelevant outcome |
| 422 | Li, 2024 | not relevant to scope | 1142 | E Loveman, 2011 | irrelevant outcome |
| 423 | Li, 2025 | not relevant to scope | 1143 | C D Summerbell, 2009 | full text not available |
| 424 | Li, 2021 | not relevant to scope | 1144 | Michael D. Jensen, 2024 | inappropriate study design |
| 425 | Li, 2022 | not relevant to scope | 1145 | Guadalupe X Ayala,2008 | irrelevant outcome |
| 426 | Liang, 2024 | not relevant to scope | 1146 | Robert P Heaney, 2013 | irrelevant outcome |
| 427 | Liang, 2022 | not relevant to scope | 1147 | Désirée C Wilks, 2011 | ineligible population |
| 428 | Licitra, 2024 | not relevant to scope | 1148 | Duong Duc Pham, 2012 | irrelevant outcome |
| 429 | Liechti, 2025 | not relevant to scope | 1149 | Jose Lara, 2014 | irrelevant outcome |
| 430 | Ligthart, 2018 | not relevant to scope | 1150 | Elissa Finkler, 2012 | irrelevant outcome |
| 431 | Luis Polo-Ferrero, 2025 | ineligible intervention | 1151 | Maryam Kazemi, 2022 | irrelevant outcome |
| 432 | Rodrigues-Santana, 2023 | ineligible intervention | 1152 | Xiaojie Yuan, 2020 | irrelevant outcome |
| 433 | Lee Ling, 2018 | not relevant to scope | 1153 | Sina Naghshi, 2020 | irrelevant outcome |
| 434 | Lim, 2019 | not relevant to scope | 1154 | Mirko Marino, 2021 | irrelevant outcome |
| 435 | Lim, 2022 | irrelevant outcome | 1155 | Marios Anemoulis, 2023 | not relevant to scope |
| 436 | Limauro, 2020 | ineligible population | 1156 | Kristin M Nieman | not relevant to scope |
| 437 | Lin, 2020 | not relevant to scope | 1157 | Emma J Boyland | not relevant to scope |
| 438 | Lin, 2017 | not relevant to scope | 1158 | Noura Alosaimi, 2023 | irrelevant outcome |
| 439 | Lin, 2022 | not relevant to scope | 1159 | Marco Antonio Hernández-Lepe, 2023 | irrelevant outcome |
| 440 | Lioret, 2023 | ineligible population | 1160 | H C Cheung, 2023 | ineligible intervention |
| 441 | Lipnicki, 2019 | not relevant to scope | 1161 | Yi-Hsuan Liu, 2021 | irrelevant outcome |
| 442 | Liska, 2019 | not relevant to scope | 1162 | Lem Ngongalah, 2018 | irrelevant outcome |
| 443 | Littlewood, 2020 | ineligible population | 1163 | Berilany Dos Santos Sena, 2020 | ineligible population |
| 444 | Liu, 2024 | not relevant to scope | 1164 | Gabrielle Viscardi, 2025 | irrelevant outcome |
| 445 | Liu, 2024 | not relevant to scope | 1165 | Yu Qi Lee, 2018 | irrelevant outcome |
| 446 | Liu, 2022 | not relevant to scope | 1166 | Edith Visser, 2024 | irrelevant outcome |
| 447 | Liu, 2023 | not relevant to scope | 1167 | Jessica Zibellini, 2015 | irrelevant outcome |
| 448 | Liu, 2025 | not relevant to scope | 1168 | Mengxuan Zou, 2022 | ineligible population |
| 449 | Liu, 2022 | irrelevant outcome | 1169 | Fan Zhong, 2024 | irrelevant outcome |
| 450 | Liu, 2016 | not relevant to scope | 1170 | Liana L Guarneiri, 2024 | irrelevant outcome |
| 451 | Liu, 2022 | not relevant to scope | 1171 | Renate L Hall, 2023 | irrelevant outcome |
| 452 | Liu, 2021 | not relevant to scope | 1172 | Solomon A Sowah, 2019 | irrelevant outcome |
| 453 | Livingstone, 2016 | not relevant to scope | 1173 | Liangyu Zhao, 2022 | ineligible population |
| 454 | Llanaj, 2022 | not relevant to scope | 1174 | Michele Barone, 2023 | ineligible population |
| 455 | Loaiza-Betancur, 2020 | not relevant to scope | 1175 | Annalisa Di Nucci, 2025 | ineligible population |
| 456 | Loef, 2025 | irrelevant outcome | 1176 | Karim Parastouei, 2020 | irrelevant outcome |
| 457 | Loh, 2020 | not relevant to scope | 1177 | Natalie D Riediger, 2018 | irrelevant outcome |
| 458 | Loperfido, 2025 | not relevant to scope | 1178 | Kiley B Vander Wyst, 2021 | ineligible population |
| 459 | Lopes, 2022 | not relevant to scope | 1179 | Nicholas Freudenberg, 2015 | irrelevant outcome |
| 460 | Kyoung-Bae Kim, 2019 | ineligible intervention | 1180 | Hadiza Abdullahi Abubakar, 2024 | ineligible population |
| 461 | Chun-De Liao, 2018 | ineligible intervention | 1181 | Alessandra Violi, 2018 | irrelevant outcome |
| 462 | Michael Nunns, 2025 | ineligible intervention | 1182 | Theresa Drabsch, 2018 | irrelevant outcome |
| 463 | Lopez, 2016 | ineligible intervention | 1183 | J Salse-Batán, 2022 | not relevant to scope |
| 464 | Lopez, 2023 | not relevant to scope | 1184 | Marta Arroyo-Huidobro, 2025 | not relevant to scope |
| 465 | Lopez, 2021 | not relevant to scope | 1185 | Mohammad Mustafa, 2025 | irrelevant outcome |
| 466 | López-Gil, 2023 | ineligible population | 1186 | Luc Tappy, 2018 | irrelevant outcome |
| 467 | López-Laguna, 2025 | not relevant to scope | 1187 | Anne-Ditte Termannsen, 2024 | irrelevant outcome |
| 468 | Lotito, 2017 | not relevant to scope | 1188 | Mi-Young Park, 2020 | irrelevant outcome |
| 469 | Lou, 2025 | irrelevant outcome | 1189 | Angelica Quatela, 2016 | not relevant to scope |
| 470 | Lovell, 2025 | not relevant to scope | 1190 | Emily K Rousham, 2020 | not relevant to scope |
| 471 | Lozano, 2016 | not relevant to scope | 1191 | Ella Moore, 2022 | irrelevant outcome |
| 472 | Lucas, 2022 | not relevant to scope | 1192 | Xue Feng Hu, 2024 | not relevant to scope |
| 473 | Luchetti, 2016 | not relevant to scope | 1193 | Sydney G O'Connor, 2024 | not relevant to scope |
| 474 | Luengo-Pérez, 2024 | not relevant to scope | 1194 | Christian S Wright, 2019 | not relevant to scope |
| 475 | Lunde, 2018 | not relevant to scope | 1195 | Arsheen Imran Sajwani, 2024 | not relevant to scope |
| 476 | Lutaud, 2023 | ineligible population | 1196 | María J Soto-Méndez, 2019 | not relevant to scope |
| 477 | Chenhan, 2017 | irrelevant outcome | 1197 | Geoffrey Livesey, 2019 | not relevant to scope |
| 478 | Xiaojun, 2024 | not relevant to scope | 1198 | Sanaz Benisi-Kohansal, 2016 | not relevant to scope |
| 479 | MacDonald, 2016 | not relevant to scope | 1199 | Carolina Pérez-Ferrer, 2019 | not relevant to scope |
| 480 | Madden, 2024 | not relevant to scope | 1200 | Rashmi Agarwal, 2025 | not relevant to scope |
| 481 | Mäder, 2025 | not relevant to scope | 1201 | Ivan Cavero-Redondo, 2019 | not relevant to scope |
| 482 | Magnussen, 2025 | not relevant to scope | 1202 | Siobhan O'Halloran,2020 | not relevant to scope |
| 483 | Daniel Borch, 2016 | ineligible intervention | 1203 | Mohamad Jawhara, 2019 | not relevant to scope |
| 484 | Nicholas Freudenberg, 2015 | ineligible intervention | 1204 | Lu Ding, 2023 | not relevant to scope |
| 485 | Jung Eun Kim, 2016 | ineligible intervention | 1205 | Lotte Hallez, 2020 | ineligible population |
| 486 | M Aceves-Martins, 2020 | ineligible intervention | 1206 | Demetrios Bitsanis, 2022 | irrelevant outcome |
| 487 | Mahboobi, 2025 | not relevant to scope | 1207 | Annalena Thimm, 2024 | not relevant to scope |
| 488 | Mahdi, 2022 | ineligible population | 1208 | Atefeh Tavakoli, 2025 | irrelevant outcome |
| 489 | Mahmood, 2020 | not relevant to scope | 1209 | Laurent Turner,2024 | not relevant to scope |
| 490 | Maicas-Pérez, 2024 | ineligible population | 1210 | Dresshti Garg, 2025 | not relevant to scope |
| 491 | Tanya J., 2018 | not relevant to scope | 1211 | Xiaoliang Wang, 2018 | not relevant to scope |
| 492 | Malambo, 2016 | not relevant to scope | 1212 | Marguerite M Conley, 2021 | not relevant to scope |
| 493 | Malińska, 2017 | non-english language | 1213 | Reeta Lamminpää, 2018 | ineligible population |
| 494 | Mallard, 2016 | irrelevant outcome | 1214 | Emily J Webb, 2021 | not relevant to scope |
| 495 | Mansouri, 2024 | not relevant to scope | 1215 | Xiao-Ni Chai, 2023 | not relevant to scope |
| 496 | Marin-Jimenez, 2022 | not relevant to scope | 1216 | Chris Kite, 2019 | not relevant to scope |
| 497 | Marklund, 2017 | not relevant to scope | 1217 | Folasade B Adeniyi, 2012 | ineligible intervention |
| 498 | Marklund, 2019 | not relevant to scope | 1218 | Jennifer S Lin, 2014 | not relevant to scope |
| 499 | Marshall, 2022 | ineligible population | 1219 | M A Plow, 2014 | not relevant to scope |
| 500 | Martin, 2018 | ineligible population | 1220 | Dimitrios Spanos, 2013 | not relevant to scope |
| 501 | Martin, 2018 | ineligible population | 1221 | Claudia Jiménez-Ten Hoevel, 2025 | not relevant to scope |
| 502 | Martin-Moraleda, 2022 | ineligible population | 1222 | Michelle M Y Wong, 2017 | not relevant to scope |
| 503 | Martineau, 2019 | not relevant to scope | 1223 | Estelle D Watson, 2017 | ineligible population |
| 504 | Martinez Guevara, 2024 | not relevant to scope | 1224 | Joanna Michalina Jurek, 2025 | not relevant to scope |
| 505 | Martínez-Ortega, 2020 | ineligible intervention | 1225 | Mohammed Hamsho, 2025 | not relevant to scope |
| 506 | Martínez-Vizcaíno, 2024 | not relevant to scope | 1226 | Parvane Saneei , 2016 | not relevant to scope |
| 507 | Mascaró, 2021 | not relevant to scope | 1227 | Seo Yun Kim, 2020 | not relevant to scope |
| 508 | Mast, 2023 | ineligible population | 1228 | Dalal Qanaq, 2024 | ineligible intervention |
| 509 | Matsuyama, 2017 | ineligible population | 1229 | Shu-Fen Chu, 2021 | not relevant to scope |
| 510 | Matwiejczyk, 2018 | ineligible population | 1230 | Natalie Pavlovic, 2021 | ineligible intervention |
| 511 | Maugeri, 2019 | not relevant to scope | 1231 | Hui Lin, 2022 | not relevant to scope |
| 512 | Mayer-Davis, 2020 | full text not available | 1232 | Catalina M Mascaró, 2021 | not relevant to scope |
| 513 | Mazarello Paes, 2015 | ineligible population | 1233 | Elizabeth K. Pritchard, 2022 | ineligible population |
| 514 | Mazidi, 2017 | not relevant to scope | 1234 | Christos Theodorakopoulos, 2017 | ineligible population |
| 515 | Mousa Khalafi, 2024 | ineligible intervention | 1235 | Asma S Alrushud, 2017 | not relevant to scope |
| 516 | Seo-Young Kim, 2022 | ineligible intervention | 1236 | Josefin Kataoka,2017 | not relevant to scope |
| 517 | Eliana V. Carraça, 2018 | ineligible intervention | 1237 | Paul N Morison, 2018 | ineligible population |
| 518 | Mamaru Ayenew Awoke, 2022 | ineligible intervention | 1238 | Aurel T. Tankeu, 2023 | not relevant to scope |
| 519 | McCartan, 2024 | not relevant to scope | 1239 | Hongli Yu, 2024 | ineligible population |
| 520 | McDaniel, 2022 | not relevant to scope | 1240 | Cristian Del Bo’ , 2023 | not relevant to scope |
| 521 | McDiarmid, 2025 | ineligible population | 1241 | Chanthawat Patikorn, 2023 | not relevant to scope |
| 522 | McLarnon, 2021 | not relevant to scope | 1242 | Heidi E. Johnston, 2022 | not relevant to scope |
| 523 | Mcluskie, 2025 | not relevant to scope | 1243 | Marianna Pellegrini, 2020 | not relevant to scope |
| 524 | McMahon, 2021 | not relevant to scope | 1244 | Eleanor M. Winpenny, 2020 | ineligible population |
| 525 | Mead, 2017 | ineligible population | 1245 | Petya Atanasova, 2022 | ineligible population |
| 526 | Meher, 2023 | not relevant to scope | 1246 | Tian Hu, 2012 | not relevant to scope |
| 527 | Mehrholz, 2017 | not relevant to scope | 1247 | Jocelyne R Benatar, 2013 | not relevant to scope |
| 528 | Meléndez-Oliva, 2025 | not relevant to scope | 1248 | Emma Boyland, 2025 | ineligible population |
| 529 | Melián-Fleitas, 2021 | not relevant to scope | 1249 | Eric Robinson, 2022 | ineligible population |
| 530 | Melville, 2025 | not relevant to scope | 1250 | Oliver T Mytton, 2014 | ineligible population |
| 531 | Melvin, 2017 | not relevant to scope | 1251 | Sara Paola Mambrini, 2023 | not relevant to scope |
| 532 | Memon, 2024 | ineligible intervention | 1252 | Oliver T Mytton, 2014 | ineligible population |
| 533 | Mendonça, 2021 | not relevant to scope | 1253 | Kirsten Corder, 2020 | ineligible population |
| 534 | Mendoza-Sarmiento, 2023 | not relevant to scope | 1254 | R.G.S. Andrade, 2024 | not relevant to scope |
| 535 | Meneu, 2024 | not relevant to scope | 1255 | David Chelmow, MD, 2022 | full text not available |
| 536 | Meng, 2023 | not relevant to scope | 1256 | C. Churuangsuk, 2018 | full text not available |
| 537 | Merino, 2019 | not relevant to scope | 1257 | Eric Conrad, 2018 | not relevant to scope |
| 538 | Mertens, 2023 | not relevant to scope | 1258 | Ehrika Vanessa Almeida de Menezes, 2019 | not relevant to scope |
| 539 | Micai, 2023 | not relevant to scope | 1259 | Marine Dupuit, 2020 | not relevant to scope |
| 540 | Michalopoulou, 2024 | ineligible population | 1260 | Eberl, 2019 | ineligible population |
| 541 | Middleton, 2018 | not relevant to scope | 1261 | Fatemeh Sadat Hashemi Javaheri, 2025 | ineligible population |
| 542 | Migliorini, 2022 | not relevant to scope | 1262 | M. Horne, 2020 | ineligible population |
| 543 | Migliorini, 2020 | not relevant to scope | 1263 | Karen Jiang, 2022 | ineligible population |
| 544 | Migliorini, 2022 | not relevant to scope | 1264 | Armin Ebrahimzadeh, 2024 | not relevant to scope |
| 545 | Migliorini, 2025 | not relevant to scope | 1265 | Ahmed Elhakeem, PhD, 2024 | ineligible population |
| 546 | Migliorini, 2023 | not relevant to scope | 1266 | Justin M. Guagliano, 2020 | ineligible population |
| 547 | Migliorini, 2024 | not relevant to scope | 1267 | Leanne Harris, 2018 | not relevant to scope |
| 548 | Migliorini, 2023 | not relevant to scope | 1268 | Xiujie He, 2015 | not relevant to scope |
| 549 | Migliorini, 2025 | not relevant to scope | 1269 | Meeke Hoedjes, 2017 | not relevant to scope |
| 550 | Migliorini, 2023 | not relevant to scope | 1270 | Annemieke Hoek M.D., Ph.D. , 2022 | ineligible population |
| 551 | Miguel, 2020 | not relevant to scope | 1271 | J Hartmann-Boyce, 2014 | not relevant to scope |
| 552 | Mikkonen, 2024 | not relevant to scope | 1272 | Mu Chen, 2012 | not relevant to scope |
| 553 | Milluzzo, 2025 | not relevant to scope | 1273 | A Kong, 2014 | not relevant to scope |
| 554 | Minoia, 2021 | not relevant to scope | 1274 | Clint T Miller, 2013 | not relevant to scope |
| 555 | Miralles, 2020 | not relevant to scope | 1275 | Adanna Ijoma, 2024 | ineligible population |
| 556 | Mitchell, 2017 | ineligible population | 1276 | Md.Mohaimenul Islam, 2020 | ineligible population |
| 557 | Moeini, 2021 | ineligible population | 1277 | Stephen Keenan, 2020 | ineligible population |
| 558 | Moghetti, 2020 | not relevant to scope | 1278 | Lisa A. Kilpatrick, 2023 | not relevant to scope |
| 559 | Moghimi, 2021 | not relevant to scope | 1279 | Adam P. Knowlden, 2022 | full text not available |
| 560 | Mohamed, 2025 | not relevant to scope | 1280 | Blossom Lake, 2022 | not relevant to scope |
| 561 | Ali Kohanmoo, 2024 | ineligible population | 1281 | L.L Leach, 2015 | not relevant to scope |
| 562 | Mohammadian Khonsari, 2022 | not relevant to scope | 1282 | Vivian Y.J. Lee, 2024 | not relevant to scope |
| 563 | Mohanannair Geethadevi, 2023 | not relevant to scope | 1283 | Fatemeh Kazeminasab, 2025 | not relevant to scope |
| 564 | Molina-Montes, 2021 | not relevant to scope | 1284 | Lisa A. Kilpatrick, 2023 | not relevant to scope |
| 565 | Montana, 2024 | not relevant to scope | 1285 | Adam P. Knowlden, 2022 | full text not available |
| 566 | Monteleone, 2022 | not relevant to scope | 1286 | Hana Koutnikova, 2019 | ineligible intervention |
| 567 | Montes-Ib, 2023 | not relevant to scope | 1287 | Blossom Lake, 2022 | not relevant to scope |
| 568 | Morgan, 2020 | ineligible population | 1288 | L.L Leach,2015 | not relevant to scope |
| 569 | Mori, 2021 | not relevant to scope | 1289 | Vivian Y.J. Lee, 2024 | not relevant to scope |
| 570 | Morison, 2018 | full text not available | 1290 | Li Ping, 2016 | ineligible intervention |
| 571 | Morris, 2019 | not relevant to scope | 1291 | Fjorida Llaha, 2020 | ineligible intervention |
| 572 | Mosdøl, 2017 | not relevant to scope | 1292 | Ava Lorenc, 2020 | ineligible population |
| 573 | Moțățăianu, 2023 | not relevant to scope | 1293 | Claire D. Madigan, 2024 | not relevant to scope |
| 574 | Mousavi, 2023 | not relevant to scope | 1294 | Amin Mirrafiei, 2024 | ineligible intervention |
| 575 | Mozaffarian, 2017 | not relevant to scope | 1295 | Ella Moore, 2022 | not relevant to scope |
| 576 | muha, 2024 | ineligible population | 1296 | John A. Naslund, 2017 | not relevant to scope |
| 577 | Muilwijk, 2018 | not relevant to scope | 1297 | William B. Nicolson, 2022 | not relevant to scope |
| 578 | Muka, 2016 | not relevant to scope | 1298 | Sabrina M Nielsen, 2017 | not relevant to scope |
| 579 | Munawaroh, 2025 | not relevant to scope | 1299 | Kelly K. O’Brien, 2016 | not relevant to scope |
| 580 | Muñoz-Urtubia, 2025 | ineligible population | 1300 | William Okoniewski, 2019 | ineligible population |
| 581 | Muriuki, 2025 | not relevant to scope | 1301 | H. M. Parretti, 2016 | full text not available |
| 582 | Musa, 2024 | ineligible population | 1302 | Michele L. Patel, 2019 | not relevant to scope |
| 583 | Musazadeh, 2024 | not relevant to scope | 1303 | Dimitrios Patoulias, 2023 | ineligible intervention |
| 584 | Musbahi, 2021 | not relevant to scope | 1304 | Peckmezian T, 2022 | ineligible intervention |
| 585 | Musilanga, 2024 | not relevant to scope | 1305 | Gwenndolyn C. Porter, 2019 | full text not available |
| 586 | Nagrani, 2020 | ineligible population | 1306 | Margaret Raber , 2021 | not relevant to scope |
| 587 | Nahaskida, 2025 | not relevant to scope | 1307 | Yu Fan, 2025 | not relevant to scope |
| 588 | Najafi, 2022 | not relevant to scope | 1308 | Yue-Qiao Wang, 2016 | not relevant to scope |
| 589 | Narciso, 2019 | ineligible population | 1309 | David J Johns 2014 | not relevant to scope |
| 590 | Narmkul, 2024 | ineligible population | 1310 | Nikolaos Mastellos, 2014 | not relevant to scope |
| 591 | Narzisi, 2021 | ineligible population | 1311 | Tiffany L Carson, 2014 | not relevant to scope |
| 592 | Naselli, 2023 | not relevant to scope | 1312 | Roshan Ravindran, 2025 | ineligible intervention |
| 593 | Navarro-Marroco, 2024 | ineligible intervention | 1313 | Anna L. Roberts, 2017 | not relevant to scope |
| 594 | Navidad, 2021 | ineligible population | 1314 | P J Rogers, 2015 | not relevant to scope |
| 595 | Ne, 2017 | irrelevant outcome | 1315 | J. Salse-Batán, 2022 | not relevant to scope |
| 596 | Negesse, 2018 | not relevant to scope | 1316 | Arashi Shahid , 2025 | not relevant to scope |
| 597 | Neil-Sztramko, 2021 | ineligible population | 1317 | Thomas Sharkey, 2020 | ineligible population |
| 598 | Neira, 2021 | not relevant to scope | 1318 | Diana Sherifali, 2017 | ineligible population |
| 599 | Neri, 2024 | not relevant to scope | 1319 | C. Shieh, 2018 | full text not available |
| 600 | Ness-Jensen, 2016 | not relevant to scope | 1320 | Jihyun Min, 2021 | ineligible population |
| 601 | Krystal Lu Shin, 2025 | not relevant to scope | 1321 | Xuling Chang, 2018 | ineligible population |
| 602 | Maggie C. Y., 2017 | not relevant to scope | 1322 | Chen, 2025 | ineligible population |
| 603 | Nguyen, 2025 | not relevant to scope | 1323 | Nastaran Mahmoudi Shirkoohi, 2025 | ineligible intervention |
| 604 | Nicholas, 2021 | not relevant to scope | 1324 | Surbhi Sood, 2025 | not relevant to scope |
| 605 | Nicholson, 2022 | not relevant to scope | 1325 |  | full text not available |
| 606 | Nielsen, 2022 | not relevant to scope | 1326 | James Steele, 2021 | ineligible population |
| 607 | Nigra, 2016 | not relevant to scope | 1327 | Stine, Jonathan G, 2023 | not relevant to scope |
| 608 | Nikooyeh, 2025 | ineligible population | 1328 | Lie Sun, 2022 | ineligible intervention |
| 609 | Nikpay, 2025 | not relevant to scope | 1329 | Derrick R Tanous, 2025 | ineligible population |
| 610 | Nilsen, 2025 | not relevant to scope | 1330 | Rafael Timon, 2025 | ineligible intervention |
| 611 | Nonguierma, 2022 | ineligible population | 1331 | Erik A Willis, 2017 | full text not available |
| 612 | Nonterah, 2021 | not relevant to scope | 1332 | Jia-ming Yang, 2022 | not relevant to scope |
| 613 | Nordlund, 2021 | ineligible population | 1333 | Yao, Junpeng BM, 2019 | ineligible intervention |
| 614 | Nti, 2024 | ineligible population | 1334 | Matteo Zago, 2018 | not relevant to scope |
| 615 | Nury, 2022 | ineligible population | 1335 | Liangyu Zhao, 2022 | ineligible population |
| 616 | O'Brien, 2022 | ineligible population | 1336 | Eliza Gaweł, 2024 | ineligible intervention |
| 617 | O’Connor, 2020 | not relevant to scope | 1337 | Lizzette Gómez-de-Regil, 2020 | ineligible intervention |
| 618 | O'Connor, 2020 | not relevant to scope | 1338 | Haire-Joshu, 2020 | ineligible intervention |
| 619 | O'Donoghue, 2021 | not relevant to scope | 1339 | M Angeles Vargas-Alvarez, 2021 | ineligible population |
| 620 | O'Neill, 2023 | not relevant to scope | 1340 | Yong Yan, 2016 | ineligible intervention |
| 621 | O'Sullivan, 2020 | ineligible population | 1341 | Mohammad Rahman Rahimi, 2025 | ineligible intervention |
| 622 | Obita, 2023 | ineligible population | 1342 | K Shaw, 2006 | full text not available |
| 623 | Ohadike, 2016 | irrelevant outcome | 1343 | E J Dhurandhar, 2015 | full text not available |
| 624 | Oja, 2024 | not relevant to scope | 1344 | Brian Hutton, 2004 | ineligible intervention |
| 625 | Ojo, 2016 | not relevant to scope | 1345 | Cynthia D Mulrow, 2008 | full text not available |
| 626 | Okeke, 2025 | not relevant to scope | 1346 | Virginia Paul-Ebhohimhen, 2009 | full text not available |
| 627 | Okesene-Gafa, 2020 | not relevant to scope | 1347 | Andrea Siebenhofer, 2021 | ineligible intervention |
| 628 | Olivas-León, 2025 | not relevant to scope | 1348 | Eric Conrad, 2018 | full text not available |
| 629 | Olsen, 2024 | not relevant to scope | 1349 | Fabricio de Souza, 2020 | ineligible population |
| 630 | Omidvar, 2019 | full text not available | 1350 | Wesam A. Debes, 2024 | inappropriate study design |
| 631 | Órla, 2020 | not relevant to scope | 1351 | Ahmed Elhakeem, PhD, 2018 | inappropriate study design |
| 632 | Orsini, 2023 | ineligible population | 1352 | Ahmed Elhakeem, PhD, 2024 | ineligible population |
| 633 | Orsso, 2024 | not relevant to scope | 1353 | Aleš Gába, 2024 | ineligible population |
| 634 | Osabi, 2023 | not relevant to scope | 1354 | Nicolás Farid Hamed Riveros, 2024 | ineligible intervention |
| 635 | Osei-Kwasi, 2020 | not relevant to scope | 1355 | J. Hartmann-Boyce, 2017 | irrelevant outcome |
| 636 | Osinga, 2024 | not relevant to scope | 1356 | Josephine S. Jakubowski, 2020 | ineligible intervention |
| 637 | Osman Mohamed, 2025 | not relevant to scope | 1357 | Robel Hussen Kabthymer, 2021 | irrelevant outcome |
| 638 | Othman, 2025 | not relevant to scope | 1358 | Ayelign Mengesha Kassie, 2020 | inappropriate study design |
| 639 | Oustric, 2018 | not relevant to scope | 1359 | George A. Kelley, 2023 | inappropriate study design |
| 640 | Owen, 2020 | not relevant to scope | 1360 | Ezgi Kolay, 2021 | inappropriate study design |
| 641 | Oye-Somefun, 2021 | not relevant to scope | 1361 | Melissa Eaton, 2024 | ineligible intervention |
| 642 | Ozcan, 2024 | not relevant to scope | 1362 | Doris Eglseer, 2023 | ineligible intervention |
| 643 | Paixão, 2020 | not relevant to scope | 1363 | Mahdieh Abbasalizad Farhangi, 2020 | ineligible intervention |
| 644 | Palmer, 2021 | not relevant to scope | 1364 | Emily Fawcett, 2020 | ineligible intervention |
| 645 | Palomo-Llinares, 2023 | not relevant to scope | 1365 | Hana Koutnikova, 2019 | ineligible intervention |
| 646 | Paluch, 2023 | not relevant to scope | 1366 | Nick S.R. Lan, 2025 | [ineligible intervention](https://www.sciencedirect.com/author/57204564807/nick-si-rui-lan) |
| 647 | Paluch, 2022 | not relevant to scope | 1367 | Kesztyüs, 2018 | ineligible intervention |
| 648 | Pan, 2021 | ineligible population | 1368 | Mona Kholdebarin, 2024 | ineligible intervention |
| 649 | Panchal, 2024 | ineligible population | 1369 | Ali Kohanmoo, 2024 | duplicate |
| 650 | Paris, 2024 | not relevant to scope | 1370 | Ezgi Kolay, 2021 | duplicate |
| 651 | Park, 2024 | ineligible population | 1371 | Valentina Álvarez-Arraño, 2021 | ineligible intervention |
| 652 | Park, 2025 | not relevant to scope | 1372 | Mohsen Aliakbarian, 2024 | ineligible intervention |
| 653 | Parsons, 2023 | not relevant to scope | 1373 | Shuning Chen, 2022 | ineligible intervention |
| 654 | Partha Sarathi, 2021 | not relevant to scope | 1374 | Lisa Dowling PhD, 2024 | ineligible intervention |
| 655 | Patel, 2018 | ineligible population | 1375 | Ana Marenco Kramer, 2023 | duplicate |
| 656 | Patil, 2018 | not relevant to scope | 1376 | Laura Kudlek, 2025 | duplicate |
| 657 | Patnode, 2017 | not relevant to scope | 1377 | Laura Kudlek, 2025 | duplicate |
| 658 | Patnode, 2022 | not relevant to scope | 1378 | Kworweinski Lafontant, 2025 | duplicate |
| 659 | Patro-Gołąb, 2016 | ineligible population | 1379 | Nick S.R. Lan, 2025 | duplicate |
| 660 | Patro-Gołąb, 2016 | not relevant to scope | 1380 | Lifu Lei, 2022 | duplicate |
| 661 | Pattaro, 2016 | not relevant to scope | 1381 | Stefan K Lhachimi, 2020 | ineligible population |
| 662 | Paulo Dos Anjos Souza Barbosa, 2019 | not relevant to scope | 1382 | Yuhong Luo, 2024 | inappropriate study design |
| 663 | Pearce, 2021 | not relevant to scope | 1383 | Rebecca Mellor, 2022 | inappropriate study design |
| 664 | Pearl, 2021 | not relevant to scope | 1384 | Richard Myers-Ingram, 2023 | inappropriate study design |
| 665 | Pearson, 2025 | ineligible population | 1385 | Chunxiao Ni, 2022 | ineligible population |
| 666 | Pechlivanidou, 2022 | ineligible population | 1386 | Wen Peng, 2022 | inappropriate study design |
| 667 | Peddle-McIntyre, 2019 | not relevant to scope | 1387 | Simone Perna, 2021 | ineligible intervention |
| 668 | Pedersen, 2025 | ineligible intervention | 1388 | Manuela Pfinder, 2020 | irrelevant outcome |
| 669 | Pellicori, 2021 | not relevant to scope | 1389 | Julia Pfisterer, 2020 | irrelevant outcome |
| 670 | Pemau, 2024 | not relevant to scope | 1390 | Sirinya Phulkerd, 2016 | inappropriate study design |
| 671 | Peng, 2021 | not relevant to scope | 1391 | Anja Roth, 2022 | irrelevant outcome |
| 672 | Peng, 2024 | not relevant to scope | 1392 | Wendi Rotunda,2024 | full text not available |
| 673 | Pérez Bedoya, 2023 | not relevant to scope | 1393 | Elizabeth Jacob, 2024 | ineligible population |
| 674 | Pérez, 2019 | not relevant to scope | 1394 | Canaan Negash Seifu, 2021 | ineligible population |
| 675 | Perge, 2024 | not relevant to scope | 1395 | Victoria Salem, 2022 | ineligible intervention |
| 676 | Phillips, 2025 | ineligible population | 1396 | Gauri Shankar, 2025 | irrelevant outcome |
| 677 | Pitchan Velammal, 2024 | not relevant to scope | 1397 | Tayebeh Shirvani, 2021 | irrelevant outcome |
| 678 | Pitt, 2017 | not relevant to scope | 1398 | Piumika SOORIYAARACHCHI , 2024 | full text not available |
| 679 | Pivetta, 2021 | not relevant to scope | 1399 | Rachael Summers, 2024 | irrelevant outcome |
| 680 | Poitras, 2017 | ineligible population | 1400 | Clara Talens, 2025 | ineligible population |
| 681 | Poitras, 2016 | ineligible population | 1401 | Chanita Unhapipatpong, 2023 | irrelevant outcome |
| 682 | Polański, 2023 | not relevant to scope | 1402 | Marleen A van Baak, 2022 | irrelevant outcome |
| 683 | Pongutta, 2022 | ineligible population | 1403 | Habib Yarizadeh, 2021 | irrelevant outcome |
| 684 | Pool, 2021 | not relevant to scope | 1404 | Francisca Zamora Zamora, 2018 | non-english language |
| 685 | Porri, 2024 | ineligible population | 1405 | Meysam Zarezadeh, 2021 | ineligible intervention |
| 686 | Potcovaru, 2023 | not relevant to scope | 1406 | Eirini-Iro Arvanitidou, 2024 | ineligible intervention |
| 687 | Pourmotahari, 2025 | not relevant to scope | 1407 | Aysha, Tuba, 2024 | ineligible intervention |
| 688 | Presta, 2024 | ineligible population | 1408 | John A. Batsis MD, 2016 | full text not available |
| 689 | Prince, 2019 | not relevant to scope | 1409 | Jeannette M. Beasley, 2020 | ineligible population |
| 690 | Probyn, 2017 | not relevant to scope | 1410 | Charis Bridger Staatz, 2021 | not relevant to scope |
| 691 | Prokopidis, 2025 | not relevant to scope | 1411 | Kate Campbell BSc, 2025 | inappropriate study design |
| 692 | Pullar, 2018 | not relevant to scope | 1412 | Mousa Khalafi, 2025 | duplicate |
| 693 | Pullar, 2019 | not relevant to scope | 1413 | Mohammed Alkharaiji, 2018 | duplicate |
| 694 | Puzzono, 2021 | not relevant to scope | 1414 | Valentina Álvarez-Arraño, 2021 | duplicate |
| 695 | Qanaq, 2024 | ineligible intervention | 1415 | Zhijian He, 2023 | duplicate |
| 696 | Qiu, 2024 | not relevant to scope | 1416 | Nicholas Freudenberg, 2015 | duplicate |
| 697 | Qu, 2025 | not relevant to scope | 1417 | Laura Kudlek, 2025 | duplicate |
| 698 | Quarmby, 2023 | not relevant to scope | 1418 | Heli Lahtio, 2022 | duplicate |
| 699 | Quirk, 2017 | irrelevant outcome | 1419 | Stephen Keenan, 2020 | duplicate |
| 700 | Raab, 2021 | ineligible population | 1420 | Clarissa Dakin, 2023 | ineligible intervention |
| 701 | Rabheru, 2025 | not relevant to scope | 1421 | Lukas Schwingshackl, 2016 | ineligible intervention |
| 702 | Racey, 2022 | not relevant to scope | 1422 | Amanda V Sardeli, 2018 | ineligible intervention |
| 703 | Rafiee, 2024 | not relevant to scope | 1423 | Dariush Mozaffarian, 2011 | ineligible intervention |
| 704 | Raghavan, 2019 | not relevant to scope | 1424 | Mansour Alblaji, 2025 | ineligible intervention |
| 705 | Raghavan, 2019 | not relevant to scope | 1425 | Mohsen Aliakbarian, 2024 | ineligible intervention |
| 706 | Raghavan, 2019 | not relevant to scope | 1426 | Esther N Muriuki, 2025 | ineligible intervention |
| 707 | Rahimi, 2025 | ineligible intervention | 1427 | B. Abiri, 2023 | not relevant to scope |
| 708 | Rahmani, 2020 | not relevant to scope | 1428 | Isma'il Kadam, 2021 | not relevant to scope |
| 709 | Rakab, 2025 | ineligible intervention | 1429 | I. Santos, 2017 | not relevant to scope |
| 710 | Ramazzina, 2017 | not relevant to scope | 1430 | Herman J. de Vries, 2016 | not relevant to scope |
| 711 | Ramírez-Vélez, 2021 | not relevant to scope | 1431 | Tianrong Liao, MM, 2024 | not relevant to scope |
| 712 | Ramsey Buchanan, 2016 | not relevant to scope | 1432 | Lisa M. Sanders, 2023 | not relevant to scope |
| 713 | Rashidbeygi, 2025 | not relevant to scope | 1433 | Han Shi Jocelyn Chew,2022 | not relevant to scope |
| 714 | Rassy, 2021 | not relevant to scope | 1434 | Xueting Wei, 2022 | not relevant to scope |
| 715 | Rawal, 2021 | not relevant to scope | 1435 | Keyhan Lotfi, 2022 | not relevant to scope |
| 716 | Raynor, 2024 | full text not available | 1436 | Kelly A. Higgins, 2022 | not relevant to scope |
| 717 | Raynor, 2024 | full text not available | 1437 | Mahdieh Abbasalizad Farhangi, , 2019 | not relevant to scope |
| 718 | Raynor, 2024 | full text not available | 1438 | Carissa van den Berk-Clark, 2018 | not relevant to scope |
| 719 | Redondo, 2018 | not relevant to scope | 1439 | Sarah E. Jarvis, 2022 | not relevant to scope |
| 720 | Ren, 2016 | not relevant to scope | 1440 | Hadeel Zaghloul, 2020 | not relevant to scope |
