# Supplementary material for: Effectiveness of dietary, physical activity, and lifestyle interventions on weight and cardiometabolic outcomes in adults with overweight or obesity: an umbrella review of 112 systematic reviews and meta-analyses from 50 countries
Source: Obes Pillars. 2026 Sep 1;20:100324. doi: 10.1016/j.obpill.2026.100324 (PMC13571594; doi:10.1016/j.obpill.2026.100324)
Supplement: Supplementary file 7 [file mmc7.docx]

**Supplemental Table 6. PRISMA 2020 checklist**

*Locations refer to the numbered sections of the manuscript as formatted for Obesity Pillars (1. Introduction; 2. Methods, with subsections 2.1–2.5; 3. Results; 4. Discussion, with subsections 4.1–4.2; 5. Conclusion; and the declaration sections that follow the Conclusion). Page and line numbers should be inserted from the final line-numbered submission PDF before uploading. AMSTAR 2, A MeaSurement Tool to Assess systematic Reviews version 2; BMI, body mass index; CI, confidence interval; GRADE, Grading of Recommendations Assessment, Development and Evaluation; PROSPERO, International Prospective Register of Systematic Reviews; RCT, randomized controlled trial; REML, restricted maximum likelihood.*

| Section and topic | Item # | Checklist item | Location where item is reported |
| --- | --- | --- | --- |
| **TITLE** |  |  |  |
| Title | 1 | Identify the report as a systematic review. | Title page (title identifies the report as an umbrella review of systematic reviews and meta-analyses) |
| **ABSTRACT** |  |  |  |
| Abstract | 2 | See the PRISMA 2020 for Abstracts checklist. | Abstract (structured: Background, Objective, Methods, Results, Conclusions); Highlights |
| **INTRODUCTION** |  |  |  |
| Rationale | 3 | Describe the rationale for the review in the context of existing knowledge. | Section 1 (Introduction), paragraphs 1–3 |
| Objectives | 4 | Provide an explicit statement of the objective(s) or question(s) the review addresses. | Section 1 (Introduction), final paragraph; Abstract (Objective) |
| **METHODS** |  |  |  |
| Eligibility criteria | 5 | Specify the inclusion and exclusion criteria for the review and how studies were grouped for the syntheses. | Section 2.1 (Search strategy and selection criteria); grouping into 5 intervention clusters and 4 follow-up strata in Section 2.3 (Intervention classification) |
| Information sources | 6 | Specify all databases, registers, websites, organisations, reference lists and other sources searched or consulted to identify studies. Specify the date when each source was last searched or consulted. | Section 2.1 PubMed as index database, restricted to English-language reports published 2015–2025; backward reference-list checking and forward citation tracking via Google Scholar. All sources last searched December 2025. |
| Search strategy | 7 | Present the full search strategies for all databases, registers and websites, including any filters and limits used. | Supplemental Table 1 (full PubMed search string, MeSH and free-text blocks, filters and limits); cited in Section 2.1 |
| Selection process | 8 | Specify the methods used to decide whether a study met the inclusion criteria of the review, including how many reviewers screened each record and each report retrieved, whether they worked independently, and if applicable, details of automation tools used in the process. | Section 2.1 records managed in Rayyan; 2 reviewers screened independently; third reviewer arbitrated disagreements |
| Data collection process | 9 | Specify the methods used to collect data from reports, including how many reviewers collected data from each report, whether they worked independently, any processes for obtaining or confirming data from study investigators, and if applicable, details of automation tools used in the process. | Section 2.1 standardized, pilot-tested extraction form; no contact with original investigators was required |
| Data items | 10a | List and define all outcomes for which data were sought. Specify whether all results that were compatible with each outcome domain in each study were sought (e.g. for all measures, time points, analyses), and if not, the methods used to decide which results to collect. | Section 2.1 primary: body weight and BMI; secondary: glycemic, lipid and blood pressure parameters. All reported time points were sought, then assigned to 4 follow-up strata (Section 2.3) |
|  | 10b | List and define all other variables for which data were sought (e.g. participant and intervention characteristics, funding sources). Describe any assumptions made about any missing or unclear information. | Section 2.1 lead author, year, number of primary studies, sample size, age, intervention components/duration/frequency/mode of delivery, effect estimates, countries and databases represented |
| Study risk of bias assessment | 11 | Specify the methods used to assess risk of bias in the included studies, including details of the tool(s) used, how many reviewers assessed each study and whether they worked independently, and if applicable, details of automation tools used in the process. | Section 2.2 (Quality appraisal and certainty of evidence) AMSTAR 2, all 16 domains, assessed independently by 2 reviewers; full appraisals in Supplemental Table 2 |
| Effect measures | 12 | Specify for each outcome the effect measure(s) (e.g. risk ratio, mean difference) used in the synthesis or presentation of results. | Section 2.4 (Data synthesis and statistical analysis) mean difference with 95% CI for weight and BMI; for fasting blood glucose and the 4 lipid outcomes, contributing reviews reported standardized as well as unstandardized mean differences, and these pooled estimates are flagged as exploratory |
| Synthesis methods | 13a | Describe the processes used to decide which studies were eligible for each synthesis (e.g. tabulating the study intervention characteristics and comparing against the planned groups for each synthesis). | Section 2.3 5 prespecified intervention clusters, with dietary interventions further sub-classified; 4 follow-up strata |
|  | 13b | Describe any methods required to prepare the data for presentation or synthesis, such as handling of missing summary statistics, or data conversions. | Section 2.4 estimates analysed in the metric reported by each contributing review; no cross-scale conversion attempted, and mixed-scale pooled estimates are reported as exploratory only |
|  | 13c | Describe any methods used to tabulate or visually display results of individual studies and syntheses. | Section 2.4 forest plots stratified by intervention type and follow-up duration; Figures 2–6 and Tables 1–2; full stratum-level plots in Supplemental Figure 1 |
|  | 13d | Describe any methods used to synthesize results and provide a rationale for the choice(s). If meta-analysis was performed, describe the model(s), method(s) to identify the presence and extent of statistical heterogeneity, and software package(s) used. | Section 2.4 random-effects model with REML estimation of between-study variance; heterogeneity quantified with I² (low <25%, moderate 25–75%, high >75%); Stata 18.0 |
|  | 13e | Describe any methods used to explore possible causes of heterogeneity among study results (e.g. subgroup analysis, meta-regression). | Section 2.4 subgroup analysis by intervention type and follow-up duration; meta-regression undertaken only where ≥10 estimates were available |
|  | 13f | Describe any sensitivity analyses conducted to assess robustness of the synthesized results. | Section 2.4 3 prespecified analyses: exclusion of small reviews (≤100 participants); leave-one-out; re-estimation under a fixed-effects model |
| Reporting bias assessment | 14 | Describe any methods used to assess risk of bias due to missing results in a synthesis (arising from reporting biases). | Section 2.4 Egger’s regression test and trim-and-fill, applied only where ≥10 estimates contributed to an outcome |
| Certainty assessment | 15 | Describe any methods used to assess certainty (or confidence) in the body of evidence for an outcome. | Section 2.2 GRADE, starting at high certainty given the RCT basis of all included reviews and downgraded for risk of bias, inconsistency, indirectness, imprecision or publication bias; full profiles in Supplemental Table 3 |
| **RESULTS** |  |  |  |
| Study selection | 16a | Describe the results of the search and selection process, from the number of records identified in the search to the number of studies included in the review, ideally using a flow diagram. | Section 3 (Results), paragraph 1 1,725 records screened → 1,552 full texts → 112 reviews included; Figure 1 (PRISMA 2020 flow diagram) |
|  | 16b | Cite studies that might appear to meet the inclusion criteria, but which were excluded, and explain why they were excluded. | Supplemental Table 5 (full citation list of all 1,440 reports excluded at full text, each with its reason for exclusion); aggregate counts by reason in Section 3, paragraph 1 |
| Study characteristics | 17 | Cite each included study and present its characteristics. | Supplemental Table 4 (all 112 included reviews: intervention, comparator, number of primary RCTs, sample size, countries, databases, AMSTAR 2 rating); cited in Section 3, paragraph 1 |
| Risk of bias in studies | 18 | Present assessments of risk of bias for each included study. | Section 3, paragraph 2 AMSTAR 2: 24 reviews (21%) high, 11 (10%) moderate, 48 (43%) low, 29 (26%) critically low confidence; per-review, per-domain assessments in Supplemental Table 2 |
| Results of individual studies | 19 | For all outcomes, present, for each study: (a) summary statistics for each group (where appropriate) and (b) an effect estimate and its precision (e.g. confidence/credible interval), ideally using structured tables or plots. | Figures 2–6 (forest plots of mean difference with 95% CI, by intervention type and follow-up duration); review-level estimates in Supplemental Figure 1 |
| Results of syntheses | 20a | For each synthesis, briefly summarise the characteristics and risk of bias among contributing studies. | Section 3, paragraphs 2–4; characteristics of contributing reviews in Supplemental Table 4 and AMSTAR 2 ratings in Supplemental Table 2 |
|  | 20b | Present results of all statistical syntheses conducted. If meta-analysis was done, present for each the summary estimate and its precision (e.g. confidence/credible interval) and measures of statistical heterogeneity. | Table 1 (weight, BMI) and Table 2 (glycemic, lipid, blood pressure), each giving pooled mean difference, 95% CI, number of contributing reviews and I²; Figures 2–6; Section 3, paragraphs 5–9 |
|  | 20c | Present results of all investigations of possible causes of heterogeneity among study results. | Section 3, penultimate paragraph (I² reported for every stratum in Tables 1–2; sources of heterogeneity discussed narratively). Subgroup analysis by intervention type and follow-up duration is presented in full in Tables 1–2 and Figures 2–6. meta-regression was prespecified where ≥10 estimates were available (Section 2.4) and 10 weight strata meet this threshold either report the meta-regression results here and in Section 3. |
|  | 20d | Present results of all sensitivity analyses conducted to assess the robustness of the synthesized results. | Section 3, final paragraph across all 3 prespecified sensitivity analyses, the direction and significance of the primary findings were unchanged |
| Reporting biases | 21 | Present assessments of risk of bias due to missing results (arising from reporting biases) for each synthesis assessed. | Egger’s regression test and trim-and-fill were prespecified for outcomes informed by ≥10 estimates (Section 2.4); 10 weight strata in Table 1 meet this threshold (low-calorie diet 3–6 and 6–12 mo; intermittent fasting <3 and 6–12 mo; other dietary <3 and 3–6 mo; physical activity 3–6 mo; lifestyle 3–6, 6–12 and >12 mo. Egger’s p values and trim-and-fill adjusted estimates here and in Section 3. |
| Certainty of evidence | 22 | Present assessments of certainty (or confidence) in the body of evidence for each outcome assessed. | Supplemental Table 3 (GRADE certainty rating for every outcome, with reasons for downgrading); referenced in Section 2.2 |
| **DISCUSSION** |  |  |  |
| Discussion | 23a | Provide a general interpretation of the results in the context of other evidence. | Section 4 (Discussion), paragraphs 1–7 |
|  | 23b | Discuss any limitations of the evidence included in the review. | Section 4.1 (Strengths and limitations) variable review quality (48 [43%] low and 29 [26%] critically low AMSTAR 2 confidence); geographic clustering in high- and upper-middle-income settings; high within-category heterogeneity |
|  | 23c | Discuss any limitations of the review processes used. | Section 4.1 single-database primary search; mixed-scale pooling for fasting blood glucose and the lipid outcomes, reported as exploratory only |
|  | 23d | Discuss implications of the results for practice, policy, and future research. | Section 4.2 (Clinical and public health implications); Section 5 (Conclusion); Highlights |
| **OTHER INFORMATION** |  |  |  |
| Registration and protocol | 24a | Provide registration information for the review, including register name and registration number, or state that the review was not registered. | Section 2.1; Title page (Registration); end of Abstract PROSPERO CRD420251245029 |
|  | 24b | Indicate where the review protocol can be accessed, or state that a protocol was not prepared. | Title page (Registration) protocol accessible via the PROSPERO record CRD420251245029 |
|  | 24c | Describe and explain any amendments to information provided at registration or in the protocol. | No amendments were made to the registered protocol. |
| Support | 25 | Describe sources of financial or non-financial support for the review, and the role of the funders or sponsors in the review. | Funding statement (after Conclusion) this study received no specific grant from any funding agency |
| Competing interests | 26 | Declare any competing interests of review authors. | Declaration of competing interest (after Conclusion) the authors declare no competing interests |
| Availability of data, code and other materials | 27 | Report which of the following are publicly available and where they can be found: template data collection form; data extracted from included studies; data used for all analyses; analytic code; any other materials used in the review. | Data availability statement (after Conclusion) the extraction dataset, the corrected covered area matrix, the code book and the analysis code are available from the corresponding author on reasonable request from the date of publication. All data analysed were extracted from the published reviews listed in the reference list. |

Reference: Page MJ, McKenzie JE, Bossuyt PM, Boutron I, Hoffmann TC, Mulrow CD, et al. The PRISMA 2020 statement: an updated guideline for reporting systematic reviews. BMJ. 2021;372:n71. doi:10.1136/bmj.n71
